# Epidemiologic and Clinical Features of Lassa Fever Outbreak in Nigeria, January 1–May 6, 2018

**DOI:** 10.3201/eid2506.181035

**Published:** 2019-06

**Authors:** Elsie A. Ilori, Yuki Furuse, Oladipupo B. Ipadeola, Chioma C. Dan-Nwafor, Anwar Abubakar, Oboma E. Womi-Eteng, Ephraim Ogbaini-Emovon, Sylvanus Okogbenin, Uche Unigwe, Emeka Ogah, Olufemi Ayodeji, Chukwuyem Abejegah, Ahmed A. Liasu, Emmanuel O. Musa, Solomon F. Woldetsadik, Clement L.P. Lasuba, Wondimagegnehu Alemu, Chikwe Ihekweazu, Nigeria Lassa Fever National Response Team

**Affiliations:** Nigeria Centre for Disease Control, Abuja, Nigeria (E.A. Ilori, O.B. Ipadeola, C.C. Dan-Nwafor, A. Abubakar, O.E. Womi-Eteng, C. Ihekweazu);; Kyoto University, Kyoto, Japan (Y. Furuse);; World Health Organization, Abuja (Y. Furuse, E.O. Musa, S.F. Woldetsadik, C.L.P. Lasuba, W. Alemu);; US Centers for Disease Control and Prevention, Abuja (O.B. Ipadeola);; African Field Epidemiology Network, Abuja (C.C. Dan-Nwafor);; Irrua Specialist Teaching Hospital, Irrua, Nigeria (E. Ogbaini-Emovon, S. Okogbenin);; Federal Teaching Hospital, Abakaliki, Nigeria (U. Unigwe, E. Ogah);; Federal Medical Centre, Owo, Nigeria (O. Ayodeji, C. Abejegah, A.A. Liasu)

**Keywords:** Lassa fever, Lassa virus, Nigeria, outbreak, epidemiology, ribavirin, viruses

## Abstract

Lassa fever (LF) is endemic to Nigeria, where the disease causes substantial rates of illness and death. In this article, we report an analysis of the epidemiologic and clinical aspects of the LF outbreak that occurred in Nigeria during January 1–May 6, 2018. A total of 1,893 cases were reported; 423 were laboratory-confirmed cases, among which 106 deaths were recorded (case-fatality rate 25.1%). Among all confirmed cases, 37 occurred in healthcare workers. The secondary attack rate among 5,001 contacts was 0.56%. Most (80.6%) confirmed cases were reported from 3 states (Edo, Ondo, and Ebonyi). Fatal outcomes were significantly associated with being elderly; no administration of ribavirin; and the presence of a cough, hemorrhaging, and unconsciousness. The findings in this study should lead to further LF research and provide guidance to those preparing to respond to future outbreaks.

## Abstract 2

The outbreak, which resulted in 423 confirmed cases and 106 deaths, was the largest recorded Lassa fever outbreak.

Lassa fever (LF) is a febrile infectious disease caused by Lassa virus. The disease was first described in Nigeria in 1969 (*1*). Rodents, particularly *Mastomys natalensis*, are considered the natural hosts of the virus (*2*). The disease is mainly spread to humans through contamination with the urine or feces of infected rats (*3*,*4*). Human-to-human transmission can occur through contact with the body fluids of infected persons; therefore, healthcare workers (HCWs) are at high risk for infection when the standard precautions for infection prevention and control are inadequate (*5*,*6*). The incubation period of the disease is 3–21 days. The clinical manifestation of the disease is nonspecific and includes fever, fatigue, hemorrhaging, gastrointestinal symptoms (vomiting, diarrhea, and stomachache), respiratory symptoms (cough, chest pain, and dyspnea), and neurologic symptoms (disorientation, seizures, and unconsciousness) (*3*). The observed case-fatality rate (CFR) among patients hospitalized for severe LF is 15%–50% (*7*,*8*). However, ≈80% of infections are considered to cause mild or no symptoms in humans and are undiagnosed (*8*). 

In Nigeria, laboratory-confirmed LF patients are treated in isolation units, according to national guidelines, to prevent community and nosocomial human-to-human infections (*9*). The country has 3 main LF treatment centers: the Irrua Specialist Teaching Hospital (Edo State), the Federal Medical Centre Owo (Ondo State), and the Federal Teaching Hospital Abakaliki (Ebonyi State) (*10*). Isolation units are also located in tertiary-care centers in other states. Ribavirin has been shown to reduce the CFR for LF (*11*); Nigeria national guidelines recommend that parenteral ribavirin be administered over a 10-day period for patients with confirmed LF (*9*).

Lassa fever is endemic to the West Africa countries of Benin, Ghana, Guinea, Liberia, Mali, Sierra Leone, and Nigeria (*7*); an estimated 300,000 LF cases occur each year in this region, resulting in ≈5,000 deaths (*8*). The annual peak of LF cases in Nigeria is observed in the dry season (December–April), and the number decreases around May (*3*). The increased likelihood of humans encountering *Mastomys* rodents and their excreta inside houses during the dry season (when these animals are seeking food) is thought to play a role in the seasonality of disease incidence (*12*). Transmission risk might be exacerbated by enhanced survival of the virus at decreased relative humidity (*13*). 

During 2014–2016, around 100 laboratory-confirmed LF cases were reported annually in Nigeria (*14*,*15*). From the end of 2017 through May 2018, the country experienced its largest recorded LF outbreak. On January 22, 2018, the Nigeria Centre for Disease Control (NCDC) activated its Emergency Operations Centre to coordinate the outbreak response (*16*). During January 1–May 6, 2018, a total of 423 laboratory-confirmed cases were reported (*17*). On May 10, 2018, NCDC announced the end of the emergency phase of the outbreak because the LF case count had consistently declined in the preceding 6 weeks and had dropped below levels considered to be a national emergency, based on historical trends in LF incidence (*10*). Here we describe the epidemiologic and clinical aspects of this LF outbreak.

## Methods

### Ethics Considerations

This investigation was performed as a part of the LF public health response in Nigeria in 2018. The investigation was not considered to be research on human subjects, as per the US Department of Health and Human Services’ Federal Policy for the Protection of Human Subjects (*18*).

### Case Definition and Laboratory Confirmation

A suspected case of LF was defined as illness meeting 1 of the following criteria: 1) >1 signs/symptoms (e.g., malaise, fever, headache, sore throat, cough, nausea, vomiting, diarrhea, myalgia, central chest pain or retrosternal pain, and hearing loss) and a history of contact with excreta or urine of rodents; 2) >1 signs/symptoms and a history of contact with a person with probable or confirmed LF within 21 days of symptom onset; or 3) inexplicable bleeding or hemorrhaging (*9*). Probable LF cases were defined as any suspected case in a patient who died without the collection of a specimen for laboratory testing (*9*). Confirmed LF cases were defined as any suspected case with a laboratory confirmation (positive for IgM antibody, reverse transcription PCR [RT-PCR], or virus isolation) (*9*). 

During the study period, blood samples from patients with suspected cases were sent to 1 of 4 laboratories: the Central Research Laboratory at College of Medicine of the University of Lagos–Lagos University Teaching Hospital (Lagos State), the Federal Teaching Hospital Abakaliki (Ebonyi State), the Institute of Lassa Fever Research and Control at the Irrua Specialist Teaching Hospital (Edo State), or the National Reference Laboratory (Federal Capital Territory). Laboratory confirmation was performed by using RT-PCR by means of the RealStar Lassa Virus RT-PCR Kit (Altona Diagnostics, https://www.altona-diagnostics.com), the LF diagnosis protocol developed by Nikisins et al. (*19*), or both. More than 95% of samples were tested by using both protocols to ensure greater sensitivity for the heterogeneous Lassa virus in Nigeria (*20*). A positive result in either or both of the protocols was regarded as positive for LF.

### Contact Tracing

Persons who had contact with patients with confirmed LF were recorded and followed up daily for 21 days by Disease Surveillance and Notification Officers (DSNOs). If contacts had symptoms, blood samples were collected and tested for LF as described.

### Data Collection and Report

All suspected LF cases were immediately reported to the DSNO for each Local Government Area and the State Epidemiologist for each state by using a surveillance reporting form developed for integrated disease surveillance and response in Nigeria (*21*). Samples were collected and tested for all suspected cases as long as the case-patient was alive (*9*). If the test was positive, detailed demographic (age, sex, and residential address), clinical (symptoms, outcome, and administration of ribavirin), and epidemiologic (occupation, onset date, and exposure history) information were collected by using a national case investigation form (CIF). All suspected, probable, and confirmed cases were line listed, and the information in the CIFs was submitted weekly by the state epidemiologists to NCDC. A summary of the figures was published weekly in a situation report (*17*); the compiled reports of the outbreak are provided in the Appendix. The projected population figures of each state were obtained from a report based on data from the National Population Commission of Nigeria and the National Bureau of Statistics (*22*). Anonymized clinical and epidemiologic data of case-patients are available by request, contingent on the recipient agreeing to appropriate guidelines for their use.

### Statistical Analyses

We conducted binomial logistic regression analyses to determine the age- and sex-adjusted odds ratio (aOR) among survived and deceased patients with laboratory-confirmed LF. Likewise, we used aORs to compare the presence of each symptom and the administration of ribavirin among these cases, adjusted for age and sex. We conducted the Mantel-Haenszel test to see the statistical trend of CFR through the outbreak. We conducted chi-square tests to detect the statistical difference in exposure history between HCWs and non-HCWs.

We performed statistical tests using SPSS version 24 (IBM, https://www.ibm.com). We computed 95% CIs and p values to test statistical significance and adjusted p values by the Bonferroni correction for multiple comparisons. A p value of <0.05 was considered statistically significant.

## Results

**Figure 1:**
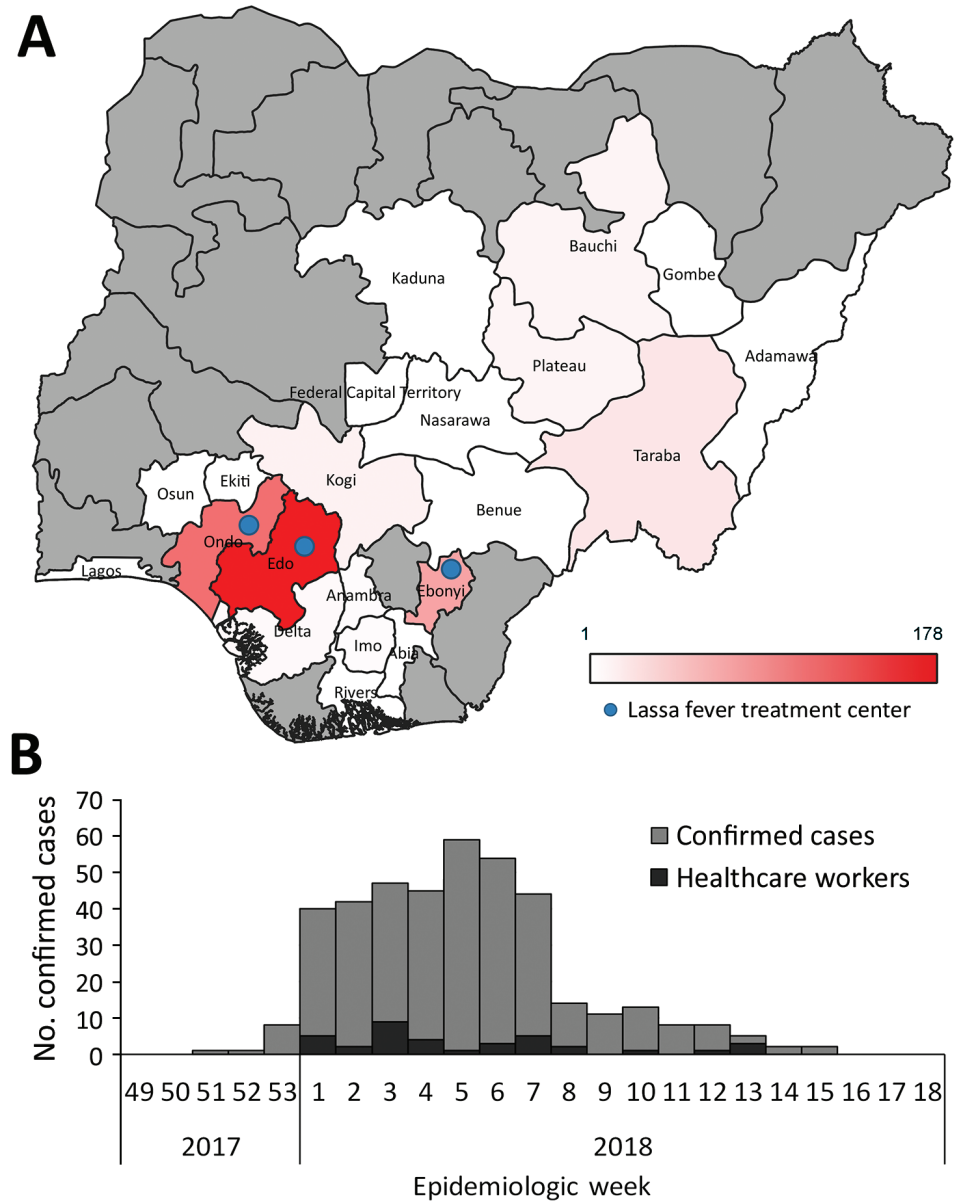
Geographic and temporal distribution of laboratory-confirmed Lassa fever cases, Nigeria, January 1–May 6, 2018. A) Geographic distribution of laboratory-confirmed cases by state. Gray shading indicates states reporting no laboratory-confirmed cases. Locations of Lassa fever treatment centers are indicated. B) Epidemic curve of laboratory-confirmed Lassa fever cases. Epidemiologic week numbers are based on the date of symptom onset.

During the study period (January 1–May 6, 2018), a total of 1,893 suspected LF cases were reported, including 10 probable cases and 423 laboratory-confirmed cases. The laboratory-confirmed cases were reported from 20 states and the Federal Capital Territory. Most (80.6%) of the laboratory-confirmed cases were reported from the 3 states with a dedicated LF treatment center: Edo (178 cases), Ondo (99 cases), and Ebonyi (64 cases) (Figure 1, panel A). LF incidence was highest in these 3 states (Table 1). Edo and Ondo also had the largest number of laboratory-negative suspected cases. The positive rate (i.e., the proportion of the number of laboratory-confirmed cases among all persons with suspected cases tested) was 22.5% (423/1,883) nationally, ranging from 3.4% (Lagos) to 70.0% (Delta). Among the 3 states with the highest number of cases, the positive rates were 16.5% (Edo), 31.6% (Ondo), and 69.6% (Ebonyi).

**Table 1:** Number and incidence of Lassa fever cases, by state or territory, Nigeria, January 1–May 6, 2018

State or territory	Population, ×1,000*	No. confirmed cases (deaths)	Case-fatality rate, %	No. confirmed cases/100,000 population	No. laboratory-negative suspected cases	No. probable cases	Positive rate, %
Abia	3,727	1 (1)	100.0	0.027	11	1	8.3
Adamawa	4,248	1 (1)	100.0	0.024	2	1	33.3
Anambra	5,528	4 (2)	50.0	0.072	3	0	57.1
Bauchi	6,537	10 (5)	50.0	0.153	50	0	16.7
Benue	5,742	1 (1)	100.0	0.017	6	1	14.3
Delta	5,663	7 (3)	42.9	0.124	3	0	70.0
Ebonyi	2,880	64 (15)	23.4	2.222	28	4	69.6
Edo	4,236	178 (26)	14.6	4.202	901	0	16.5
Ekiti	3,271	2 (1)	50.0	0.061	10	0	16.7
Federal Capital Territory	3,564	3 (2)	66.7	0.084	38	0	7.3
Gombe	3,257	2 (2)	100.0	0.061	13	0	13.3
Imo	5,409	4 (1)	25.0	0.074	11	0	26.7
Kaduna	8,252	1 (1)	100.0	0.012	4	0	20.0
Kogi	4,474	11 (4)	36.4	0.246	15	2	42.3
Lagos	12,551	1 (1)	100.0	0.008	28	0	3.4
Nasarawa	2,523	3 (2)	66.7	0.119	34	0	8.1
Ondo	4,672	99 (24)	24.2	2.119	214	1	31.6
Osun	4,706	2 (1)	50.0	0.043	2	0	50.0
Plateau	4,200	9 (7)	77.8	0.214	39	0	18.8
Rivers	7,304	1 (1)	100.0	0.014	7	0	12.5
Taraba	3,067	19 (5)	26.3	0.620	41	0	31.7
Total	193,393	423 (106)	25.1	0.219	1,460	10	22.5

*Data source: National Bureau of Statistics (*22*).

CFR among the laboratory-confirmed cases was 25.1% (106/423 [95% CI 20.9%–29.2%]). Among the 3 most affected states, CFR was 14.6% (Edo), 24.2% (Ondo), and 23.4% (Ebonyi) (Table 1). Among the 423 cases, a total of 414 CIFs with detailed information, including demographic information, onset date, symptoms, exposure history, and ribavirin administration, were collected (collection rate 97.9%). However, the data in some fields of the CIFs were incomplete. For example, the onset date was unknown in 2.4% (10/414) of cases, and symptom information was missing in 12.8% (53/414). An epidemic curve based on the onset date for laboratory-confirmed cases peaked at epidemiologic week 5 in 2018 (Figure 1, panel B). No statistically significant change was observed in the national CFR throughout the outbreak (p value for trend = 0.41).

We analyzed the age and sex distribution of the 414 patients with laboratory-confirmed cases (Table 2). Median age was 32 years (interquartile range 20–44 years); 157 (37.9%) were female and 257 (62.1%) male. CFR was lowest in children <10 years of age (11.1%) and highest in adults >61 years of age (38.2%); the aOR of fatal outcomes in the elderly group was 4.9 (95% CI 1.5–15.6) compared with children <10 years of age. Adults 41–60 years of age also had statistically higher CFRs compared with children <10 years of age (Table 2). CFR was higher for male patients (26.6%) than female patients (21.8%) but was not significantly different; the aOR of fatal outcomes in male patients compared with female patients was 1.3 (95% CI 0.81–2.1).

**Table 2:** Distribution of age and sex among patients with laboratory-confirmed Lassa fever, Nigeria, January 1–May 6, 2018*

Characteristic	No. girls and women (deaths)	No. boys and men (deaths)	Total no. (deaths)	Case-fatality rate, %	aOR (95% CI)	p value
Age group, y						
0–10	19 (3)	26 (2)	45 (5)	11.1	Reference	
11–20	24 (4)	42 (10)	66 (14)	21.2	2.1 (0.71–6.4)	0.18
21–30	38 (5)	52 (13)	90 (18)	20.5	2.1 (0.71–6.0)	0.18
31–40	30 (7)	60 (16)	90 (23)	25.6	2.7 (0.95–7.6)	0.063
41–50	23 (5)	39 (15)	62 (20)	32.3	3.8 (1.3–11.0)	0.015
51–60	10 (4)	15 (4)	25 (8)	32.0	3.8 (1.1–13.2)	0.039
>61	12 (5)	22 (8)	34 (13)	38.2	4.9 (1.5–15.6)	0.0074
Total	157 (34)	257 (68)	423 (106)			
Case-fatality rate, %	21.8	26.6				
aOR (95% CI)	Reference	1.3 (0.81–2.1)				
p value		0.26				

*aORs, 95% CIs, and p values calculated by using the binomial logistic regression model for fatal outcomes. Because information on age and sex was missing for some cases, the number of total cases is not equal to sum of the number of cases in all age groups. aOR, adjusted odds ratio.

The most common signs and symptoms among patients with laboratory-confirmed LF were fever (96.4%, 348/361), headache (58.7%, 210/358), vomiting (49.4%, 177/358), fatigue (43.3%, 155/358), and abdominal pain (40.2%, 144/358) (Table 3). Hemorrhaging was observed in 17.0% (61/358) of these patients. Cough (p = 0.0050), hemorrhaging (p<0.001), and unconsciousness (p = 0.0018) were significantly more prevalent in fatal than nonfatal cases.

**Table 3:** Prevalence of symptoms and outcomes among patients with laboratory-confirmed Lassa fever, Nigeria, January 1–May 6, 2018*

Sign/symptom	Cases, % (no. positive/no. with data available)			aOR (95% CI)	p value
	All cases	Fatal cases	Nonfatal cases		
Fever	96.4 (348/361)	97.8 (87/89)	96.3 (260/270)	1.5 (0.31–7.3)	1
Headache	58.7 (210/358)	64.0 (57/89)	56.6 (151/267)	1.4 (0.83–2.3)	1
Vomiting	49.4 (177/358)	56.2 (50/89)	47.6 (127/267)	1.5 (0.88–2.4)	1
Fatigue	43.3 (155/358)	55.1 (49/89)	39.7 (106/267)	1.5 (0.93–2.6)	1
Abdominal pain	40.2 (144/358)	49.4 (44/89)	37.1 (99/267)	1.7 (1.0–2.9)	0.68
Anorexia	33.0 (118/358)	39.3 (35/89)	31.1 (83/267)	1.4 (0.81–2.3)	1
Cough	30.4 (109/358)	46.1 (41/89)	25.5 (68/267)	2.6 (1.6–4.4)	0.0050
Diarrhea	26.8 (96/358)	39.3 (35/89)	22.8 (61/267)	2.2 (1.3–3.7)	0.068
Sore throat	22.1 (79/358)	32.6 (29/89)	18.7 (50/267)	2.0 (1.1–3.5)	0.33
Chest pain	21.3 (76/357)	26.1 (23/88)	19.9 (53/267)	1.4 (0.76–2.4)	1
Myalgia	18.5 (66/357)	28.4 (25/88)	15.0 (40/267)	2.1 (1.2–3.8)	0.28
Hemorrhaging	17.0 (61/358)	37.1 (33/89)	10.1 (27/267)	5.1 (2.8–9.3)	<0.001
Arthralgia	16.5 (59/357)	26.1 (23/88)	13.5 (36/267)	2.3 (1.2–4.3)	0.17
Dyspnea	14.8 (53/357)	25.0 (22/88)	11.6 (31/267)	2.6 (1.4–4.9)	0.061
Unconsciousness	4.8 (17/357)	13.6 (12/88)	1.9 (5/267)	9.4 (3.1–28.7)	0.0018
Conjunctivitis	4.5 (16/358)	6.7 (6/89)	3.7 (10/267)	2.1 (0.71–6.1)	1
Disorientation	4.2 (15/357)	8.0 (7/88)	3.0 (8/267)	2.8 (0.97–8.3)	1
Skin rash	3.6 (13/358)	6.7 (6/89)	2.6 (7/267)	3.0 (0.97–9.6)	1
Photophobia	3.4 (12/357)	6.8 (6/88)	2.2 (6/267)	3.0 (0.92–9.9)	1
Hiccup	2.5 (9/358)	6.7 (6/89)	1.1 (3/267)	6.6 (1.6–28.0)	0.22
Jaundice	2.2 (8/358)	4.5 (4/89)	1.5 (4/267)	3.7 (0.87–15.6)	1

*aORs and 95% CIs calculated by using a binomial logistic regression model for fatal outcomes adjusted for age and sex. p values calculated by using a binomial logistic regression model and adjusted by the Bonferroni correction. aOR, adjusted odds ratio.

**Figure 2:**
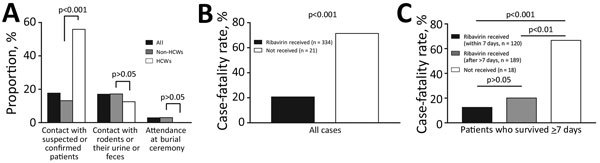
Exposure history and case-fatality rate among patients with laboratory-confirmed Lassa fever cases, Nigeria, January 1–May 6, 2018. A) Proportion of persons reporting Lassa fever exposure risks for all case-patients, HCWs, and non–HCWs. To assess differences in exposure risks between HCWs and non–HCWs, p values were calculated by using the χ^2^ test and adjusted by the Bonferroni correction. B) The case-fatality rate for case-patients who did or did not receive ribavirin. C) An investigation of the case-fatality rate in patients who survived >7 days after symptom onset. For panels B and C, p values were calculated by using binomial logistic regression analysis adjusted for age and sex and applying the Bonferroni correction. HCW, healthcare worker.

During the 3 weeks before symptom onset, 17.7% (62/350) of case-patients reported contact with patients who had known suspected or confirmed LF, 17.0% (56/330) reported contact with rodents or their urine or feces, and 2.9% (9/315) reported attendance at a burial ceremony (Figure 2, panel A). During the study period, 5,012 persons were determined to have had contact with confirmed case-patients; follow-up was conducted for 5,001 of them. During the 21-day follow-up period, 81 contacts experienced onset of symptoms, and 28 were found to have laboratory-confirmed LF. The positive rate among symptomatic contacts was 34.6% (28/81), and the secondary attack rate was 0.56% (28/5,001; 95% CI 0.35%–0.77%).

During the study period, 37 HCWs were infected, resulting in 8 deaths (CFR 21.6%). The incidence of HCW infections was distributed throughout the outbreak period (Figure 1, panel B). A significantly high proportion of HCWs (55.9%, 19/34) reported contact with patients with known suspected or confirmed LF compared with non-HCWs (p<0.001) (Figure 2, panel A).

Ribavirin was administered to 94.1% (334/355) of the patients with laboratory-confirmed cases. CFR for patients who received ribavirin was 20.7% (69/334), compared with 71.4% (15/21) for patients who did not receive ribavirin (Figure 2, panel B). We also analyzed the subset of patients who survived >7 days after symptom onset to account for the possible effect of the difference in clinical conditions. We further divided the patients who received ribavirin into 2 groups: patients who received the drug within 7 days after symptom onset and patients who received the drug after that point. Among case-patients who survived >7 days, CFR was significantly higher for patients who did not receive any ribavirin (66.7%, 12/18) compared with patients who received the drug (p<0.01), whether receipt of the drug occurred within 7 days of symptom onset or >7 days after onset (Figure 2, panel C). CFR was lower among patients who received the drug within 7 days of symptom onset (12.5%, 15/120) compared with patients who received the drug after that point (20.1%, 38/189), although this difference was not significant (p = 0.095). Because this reduction in CFR might have been attributable to not only ribavirin but also the other supportive treatments provided, the time between symptom onset date and the patient’s arrival at a health facility was included in addition to age and administration of ribavirin as a covariable in the binomial logistic regression analysis for fatal outcomes. Although absence of ribavirin administration (p<0.001) and advanced age (p = 0.025) remained significant factors in fatal outcomes, delay in visiting health facility did not (p = 0.19).

## Discussion

We describe the epidemiologic and clinical features of the LF outbreak in Nigeria during January 1–May 6, 2018. A total of 423 laboratory-confirmed cases were reported during the study period. Most of the laboratory-confirmed cases were reported from the 3 states (Edo, Ondo, and Ebonyi) where dedicated LF treatment centers are located; disease incidence was also highest in these areas. However, the positive rate among suspected cases was not especially high for these 3 states. In addition to the high prevalence of LF in the areas, HCWs in these 3 states had a high suspicion of LF in patients with high-grade fevers, which might have led to increased testing. Conversely, suspicion of LF might be low in some areas other than these 3 states. Lack of LF expertise and diagnostic capacity in these other areas might have discouraged active detection of LF patients, leading to underreporting of the disease. Also, LF incidence and prevalence might actually be lower in some areas for epidemiologic and environmental reasons, such as low prevalence of the virus in rodents or good hygiene practices that help reduce contact between humans and rodents. To clarify different LF prevalence by areas, sensitization and strengthening of the surveillance system to detect suspected cases and obtain test samples are required, particularly in states other than Edo, Ondo, and Ebonyi. In addition, seroprevalence surveys and ecologic studies of LF in humans and rodents should give further insight on the actual burden of the disease.

We used 2 protocols for molecular diagnosis of LF to cover the heterogeneous Lassa virus in Nigeria (*20*), and we tested >95% of the samples by using both protocols during the outbreak. Further study is needed to reveal sensitivity and specificity of each protocol in this country. That information would also give us further insight on genetic diversity of the virus in natural hosts in the country.

Although NCDC situation reports showed the peak of the outbreak at week 7 (*17*), our study found that the outbreak peaked at week 5. This difference occurred because the epidemic curve from the situation reports was based on the reporting date, whereas the epidemic curve in our study was based on symptom onset date. The difference can be explained by the time lag between symptom onset to health facility presentation and subsequent diagnosis and reporting. The median of the time lag between symptom onset to suspicion of LF was 7 days (interquartile range 4–11 days), and several additional days were required for a health facility to report through the DSNO for each Local Government Area and for the state epidemiologist in each state to report NCDC. Further strengthening of the surveillance system is required to shorten this time gap.

Girls and women accounted for a lower proportion of the laboratory-confirmed LF cases than boys and men (37.9% vs. 62.1%). Past studies have shown no or little difference in LF incidence between male and female patients (*23*–*25*). It is unclear whether the difference in this study came because men and boys were at higher risk for infection or more susceptible to the disease than women and girls or because ascertainment of cases in women and girls was low during this outbreak.

CFR among laboratory-confirmed cases during the study period was 25.1% and did not change substantially throughout the outbreak. CFR can reach 50% in hospitalized patients during epidemics (*8*), whereas the observed CFR among patients hospitalized with severe LF is 15% (*7*). CFR in this outbreak was especially high among elderly patients. Such difference in CFR among age groups was not observed in previous studies (*26*,*27*). The large number of cases in this outbreak might have increased the statistical power to detect the difference.

Gastroenteric symptoms, including vomiting, abdominal pain, and anorexia, were frequently observed among patients with laboratory-confirmed cases (>30%), whereas hemorrhaging was only observed in 17.0% of case-patients (Table 3). This observation is consistent with previous reports (*28*,*29*). Because some symptoms, such as cough, hemorrhaging, and unconsciousness, were more frequently observed for fatal cases than nonfatal cases, these symptoms might be predictors for fatal outcomes. Patients with such symptoms would require more attention to achieve better clinical outcomes.

Lassa virus is primarily transmitted to humans from rodents. The virus is also occasionally transmitted through the body fluids of infected persons (*3*). In this outbreak, ≈17% of patients reported a history of contact with rodents. A similar percentage reported contact with patients with suspected or confirmed LF. Because this information was obtained by patient interview, recall bias might have influenced the accuracy of exposure history. Although the secondary attack rate was as low as 0.56%, the positive rate for LF among person with suspected cases was higher for those who had contact with confirmed case-patients than that in the general population (34.6% vs. 22.5%). Contact tracing did not account for 34 LF patients, although they had reported contact with other confirmed or suspected case-patients. Some case-patients might have been targeted in the contact tracing whereas others were not because they reported contact with persons with suspected (but not confirmed) cases. Strengthening and expanding contact tracing is required but posed a challenge in resource-limited settings during the outbreak. A high rate of contact with suspected or confirmed LF case-patients among HCWs suggests the possibility of nosocomial infections, although we could not rule out another source of infection, such as rodents (*30*). Nevertheless, good infection prevention and control practices and readily available personal protective equipment are important to protect HCWs from infection with the virus (*5*,*6*).

The findings in this study support the effectiveness of ribavirin in reducing mortality rates from LF. CFR was lowest among patients who received treatment with ribavirin. Patients in severe conditions might have not received ribavirin because they died before reaching healthcare facilities where treatment was available; that is, the difference in CFRs between patients who did or did not receive ribavirin might be attributable not only to the ribavirin treatment but also to the patient’s clinical condition before ribavirin administration (*6*,*28*). To explore this issue further, we analyzed a subset of patients who survived >7 days after symptom onset. The highest CFR was still observed among patients who did not receive ribavirin; CFR was lower even if provision of ribavirin was deferred to >7 days after symptom onset. Furthermore, the early commencement of ribavirin treatment reduced CFR compared with deferred administration of the drug, although this difference was not statistically significant. Therefore, the reduction in CFR was more likely attributable to ribavirin than to the other supportive treatments provided. These findings support Nigeria’s national guidelines for clinical management of the disease, which advise that patient outcomes are more favorable when ribavirin treatment is commenced earlier (*9*).

This study has several limitations. Although we collected CIFs from 414 of the 423 patients with laboratory-confirmed cases, the data in some fields were incomplete. Availability of CIFs for patients with suspected cases whose laboratory tests were negative would have enabled us to conduct a case–control study to better determine the risk factors for the disease; however, collection of this information for 1,460 laboratory-negative suspected cases would have been burdensome to outbreak response staff. Also, our study did not provide any insights into why the 2018 LF outbreak was larger than those in previous years. Studies using viral genomic data have suggested that the outbreak was not caused by ongoing human-to-human transmission from a single source but by multiple environmental sources (*31*). Better surveillance through increased availability of laboratory testing of suspected cases might have played a role in the larger number of laboratory-confirmed cases during this outbreak. Localized clusters, which were not investigated as part of this study, might have contributed to the inflation of case numbers in some areas.

LF is endemic in Nigeria. The LF surveillance data from Nigeria, even with their limitations, are arguably the best longitudinal data collected on LF globally. Although these data mostly relate to the 2018 outbreak, data collection has continued, and all aspects of surveillance are being continuously improved (e.g., educating HCWs on the case definition, increasing ease and efficiency of the transport of samples, and increasing capacity and quality of diagnosis). In this study, we described the epidemiologic and clinical features of the largest recorded LF outbreak, which had a high CFR. The investigation also revealed several risk factors for fatal outcomes and the contribution of early treatment in reduction of CFR. These findings should lead to further investigation of the disease. Our study also highlights the need for specific incidence and seroprevalence surveys to determine the actual burden of disease in Nigeria and West Africa. Although the emergency phase of this outbreak was declared over on May 10, 2018, a small number of cases in some areas continued to be reported thereafter (*10*). Ceaseless efforts to improve risk communication, surveillance, laboratory diagnosis, clinical management, infection prevention and control practices, logistics, and coordination could mitigate the impact of future outbreaks.

Appendix Compilation of situation reports issued by the Nigeria Centre for Disease Control during a Lassa fever outbreak, Nigeria, January 1–May 6, 2018.
