# Supplementary material for: Epidemiologic and Clinical Features of Lassa Fever Outbreak in Nigeria, January 1–May 6, 2018
Source: Emerg Infect Dis. 2019 Jun;25(6):1066–74. doi: 10.3201/eid2506.181035 (PMC6537738; doi:10.3201/eid2506.181035)
Supplement: Appendix — Compilation of situation reports issued by the Nigeria Centre for Disease Control during a Lassa fever outbreak, Nigeria, January 1–May 6, 2018. [file 18-1035-Techapp-s1.pdf]

Article DOI: https://doi.org/10.3201/eid2506.181035

Epidemiologic and Clinical Features of Lassa Fever Outbreak in Nigeria, January 1–May 6, 2018

Appendix

Compilation of Situation Reports Issued by the Nigeria Centre for Disease Control during Lassa Fever Outbreak, Nigeria, January 1–May 6, 2018

(See following pages)

SITUATION REPORT
Nigeria Centre For Disease Control (NCDC)

www.ncdc.gov.ng PLOT 800 EBITU UKIWE STREET, JABI ABUJA, NIGERIA. **TOLL FREE CALL:** 0800-970000-10. **E:** info@ncdc.gov.ng @NCDCgov

**TITLE:**	**2018 LASSA FEVER OUTBREAK IN NIGERIA**
**SERIAL NUMBER:**	**01**
**EPI-WEEK:**	**01**
**DATE:**	**07 January, 2018**

HIGHLIGHTS

- In the reporting Week 01 (01-07 January, 2018), one(1) new confirmed[i] case was reported from Bauchi State
- So far in 2018, 1st – 7th January, only Bauchi State has recorded one (1) confirmed case of Lassa fever. Figure 1
- The outbreak is however active[ii] in five (5) states, namely Bauchi, Plateau, Edo, Ondo and Nasarawa States, an overflow from December 2017

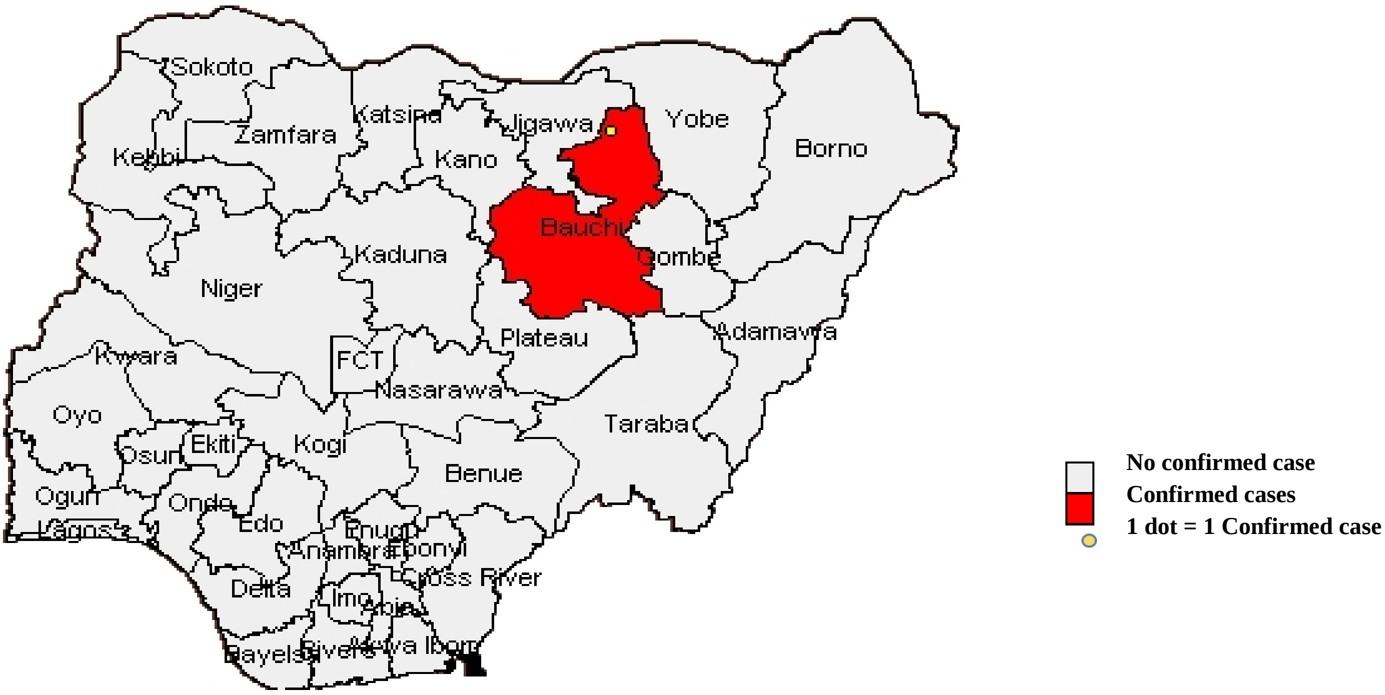

Figure 1. Distribution of Confirmed Lassa Fever cases in Nigeria as at 7th January, 2018

---

[i] *Any suspected case with laboratory confirmation (positive IgM antibody, PCR or virus isolation).*

[ii] *"Active" means where there has been at least one confirmed case, and contacts within 21 days post exposure*

TITLE:	2018 LASSA FEVER OUTBREAK IN NIGERIA
SERIAL NUMBER:	02
EPI-WEEK:	02
DATE:	14 January, 2018

HIGHLIGHTS

- In the reporting Week 02 (January 08-14, 2018) twenty-four (24) new suspected[i] cases were reported from Ondo (17), Nasarawa (4) and Bauchi (3) States.
- Seventeen (17) new confirmed[ii] cases were recorded from four States, Ondo (8) Edo (4) Ebonyi (4) and Anambra (1) with 3 deaths in Ebonyi among Health Care workers.
- From 1st – 14th January 2018, a total of fifty-six (56) suspected and twenty-two (22) confirmed[ii] cases with 3 deaths have been reported from six States -Bauchi, Edo, Ondo, Nasarawa, Ebonyi and Anambra States. *Figure 1*
- Case Fatality Rate in confirmed cases is 13.6%.
- Ebonyi state recorded a cluster of infection in health workers at the Federal Teaching Hospital, Abakaliki (FETHA). Four health workers were affected as at 14th January 2018 with 3 deaths recorded (2 Doctors and 1 Nurse). One doctor undergoing treatment at Irrua.
- The outbreak is currently active[iv] in 5 states (Edo, Ondo, Bauchi Nasarawa Ebonyi and Anambra). Plateau State has completed 42 days follow-up (two incubation periods) with no new confirmed case reported and the outbreak is over in the state
- NCDC staff and NFELTP residents (National RRT) deployed to Ebonyi state to support the state
- NCDC in collaborating with ALIMA in Edo and Ondo States for assessment of isolation Centres

Table 1: Active Outbreak Summary

Completed 42 days since last confirm<br>**Description**	**Edo**	**Ondo**	**Bauchi**	**Plateau**	**Nasarawa**	**Ebonyi**	**Anambra**	*week*<br>**TOTAL**
**Cases**								
New laboratory confirmed	4	8	0	0	0	4	1	**17**
Probable Cases	0	0	0	0	0	0	0	**0**
New Suspected	0	17	3	0	4	0	0	**24**
**Total laboratory confirmed**	**4**	**12**	**1**	**0**	**0**	**4**	**1**	**22**
Total suspected cases (including pending laboratory result and unclassified)	15	11	6	0	4	0	0	**36**
**Total cases reported (confirmed + probable+ suspected)**	**19**	**22**	**6**	**0**	**4**	**4**	**1**	**56**
Total number currently in treatment facility				0	0	1		**1**
**Deaths**								
Newly Reported	0	0	0	0	0	3	0	**3**
Total deaths in confirmed cases	0	0	0	0	0	3	0	**3**
Total Deaths in probable cases	0	0	0	0	0	0	0	**0**
Total deaths in suspected cases	0	0	0	0	0	0	0	**0**
**Total Deaths**	**0**	**0**	**0**	**0**	**0**	**3**	**0**	**3**

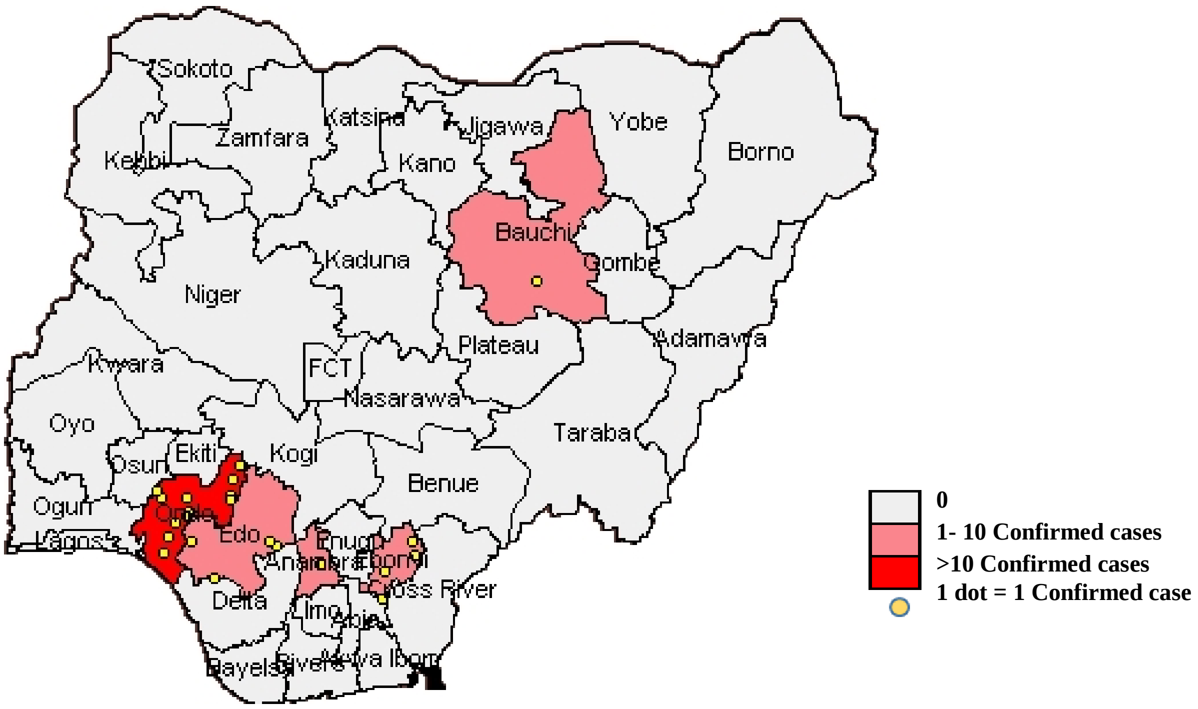

Figure 1. Distribution of Confirmed Lassa Fever cases in Nigeria as at 14th January, 2018

Response

Coordination

- NCDC Lassa fever response working group is leading coordination of weekly Lassa fever review meeting in conjunction with MDAs (FMARD and FMoE) and partners (WHO, CDC, UMB, AFENET, and MSF)
- Team consisting of NCDC staff and NFELTP residents deployed to respond to Ebonyi Outbreak
- NCDC in collaborating with ALIMA in Edo and Ondo States for assessment of isolation Centre and states' level of preparedness for 2018 outbreak

Case management, Infection Prevention and control

- Confirmed cases are being treated at identified treatment/isolation centres across the states with Ribavirin and necessary supportive management also instituted. Every state should have identified an isolation unit.

Surveillance

- Contact tracing and enhanced surveillance ongoing in affected states with an active outbreak through the State Surveillance Team
- Line listing of cases reported across all the states is ongoing, updated per time and uploaded on the VHF management system

Research and development

- Plans in collaboration with WHO to conduct seroprevalence survey and research to develop rapid test kit for diagnosis of LF

Actions

- Assessment of isolation centres in Nigeria ongoing
- Plans to conduct rapid assessment and intervention for high burden states- Edo and Ondo
- Plans to deploy outbreak data management tool –Surveillance, Outbreak Response, Management and Analysis System (SORMAS)– to affected states
- Follow up with Ondo and Edo States for retrieval of the new confirmed cases in VHF investigation form.
- Follow up with states to retrieve the line list of all 2017 Lassa fever cases
- Harmonization of Laboratory data with surveillance data ongoing
- Report of the 2016/2017 Lassa fever season

Challenges

- States not responding to submission of completed VHF forms and line list
- Twenty six states plus FCT yet to submit LF preparedness checklist

---

[i] *Suspected case describes any individual presenting with one or more of the following: malaise, fever, headache, sore throat, cough, nausea, vomiting, diarrhoea, myalgia, chest pain, hearing loss and either a. History of contact with excreta or urine of rodents b. History of contact with a probable or confirmed Lassa fever case within a period of 21 days of onset of symptoms OR Any person with inexplicable bleeding/hemorrhagia.*

[ii] *Any suspected case with laboratory confirmation (positive IgM antibody, PCR or virus isolation).*

[iii] *Any suspected case (see definition above) but who died without collection of specimen for laboratory testing*

[iv] *"Active" means where there has been at least one confirmed case, and contacts within 21 days post exposure*

Disclaimer – The information contained in this document is confidential, privileged and only for the intended recipient and may not be used, published or redistributed to the public. A redacted version is available on http://ncdc.gov.ng/diseases/sitreps

TITLE:	2018 LASSA FEVER OUTBREAK IN NIGERIA
SERIAL NUMBER:	03
EPI-WEEK:	03
DATE:	21 January, 2018

HIGHLIGHTS

- **In the reporting Week 03 (January 15-21, 2018) thirty-four new confirmed[ii] cases** and two probable[iii] cases with 14 deaths were recorded from eight States Edo (11), Ondo (14), Nasarawa (1), Ebonyi (4), Benue (1), Kogi (1) Lagos (1) , and Imo (1)
- From 1st – 21st January 2018, a total of **107 suspected[i] cases**, and 16 deaths have been reported from 10 active[iv] States- (Edo, Ondo, Bauchi, Nasarawa, Ebonyi, Anambra, Benue, Kogi, Imo, and Lagos). *Figure 2*
- Since the beginning of 2018, 64 cases have been classified as: **61 confirmed** cases, 3 probable cases with 16 deaths (13 deaths in confirmed and 3 in probable)
- Case Fatality Rate in confirmed and probable cases is 25.0% and 14.9% for all cases (including probable, confirmed and suspected)
- **Ten Health Care workers have been affected in four states** –Ebonyi (7), Nasarawa (1) Kogi (1) and Benue (1) with four deaths in Ebonyi (3) and Kogi (1)
- NCDC staff and NFELTP residents (National RRT) deployed to Ebonyi and Ondo state to support the States
- Irrua Specialist Hospital had 22 confirmed cases on admission this weekend. FMC Owo has 10 isolation beds, all occupied. Colleagues in Irrua are also providing clinical management advise for other hospitals
- NCDC supplied Irrua and Owo tents and beds this weekend for their surge capacity
- NCDC in collaborating with ALIMA in Edo and Ondo States for assessment of isolation Centres
- EOC established from Monday 22 January 2018 to coordinate the response

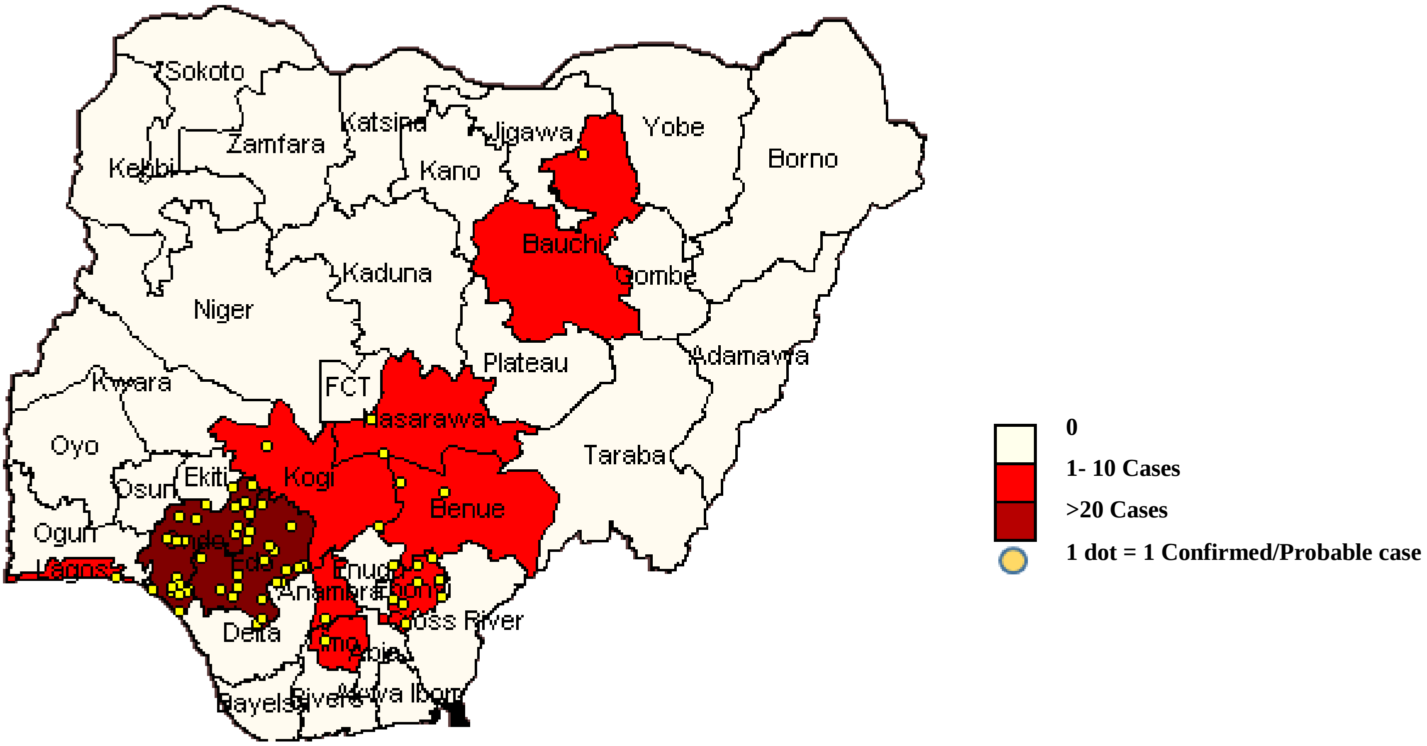

Figure 1. Distribution of Confirmed/Probable Lassa Fever cases in Nigeria as at 21st January, 2018

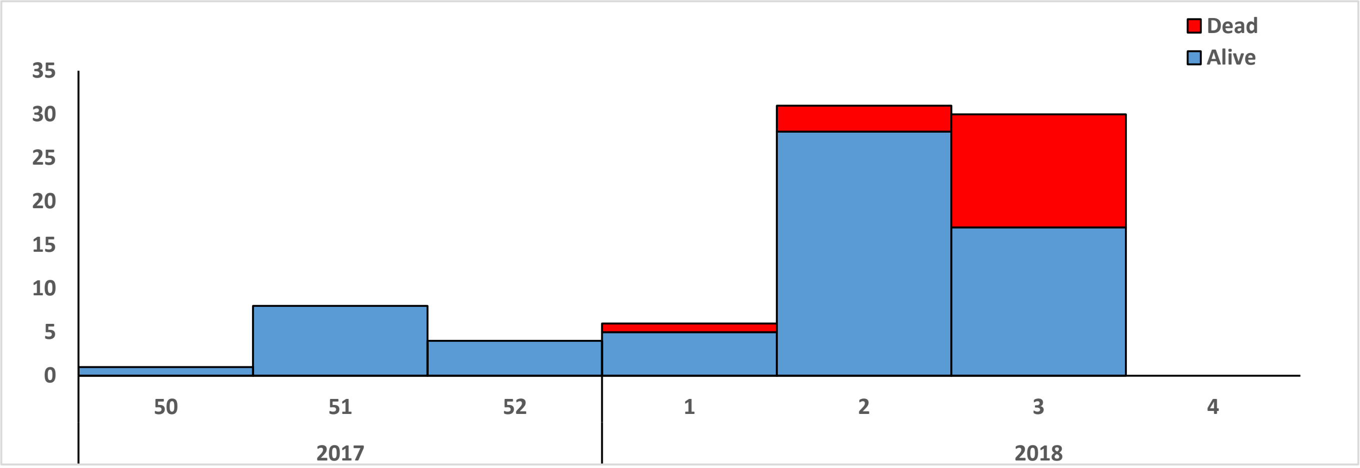

Figure 2. Confirmed and Probable Cases of Lassa fever by epidemiological week in Nigeria – 2017 50 week 1-3 2018

---

[i]Suspected case describes any individual presenting with one or more of the following: malaise, fever, headache, sore throat, cough, nausea, vomiting, diarrhoea, myalgia, chest pain, hearing loss and either a. History of contact with excreta or urine of rodents b. History of contact with a probable or confirmed Lassa fever case within a period of 21 days of onset of symptoms OR Any person with inexplicable bleeding/hemorrhagia.

[ii]Any suspected case with laboratory confirmation (positive IgM antibody, PCR or virus isolation).

[i]Any suspected case (see definition above) but who died without collection of specimen for laboratory testing

[iii]Any suspected case (see definition above) but who died without collection of specimen for laboratory testing

[iv] "Active" means where there has been at least one confirmed case, and contacts within 21 days post exposure

TITLE:	2018 LASSA FEVER OUTBREAK IN NIGERIA
SERIAL NUMBER:	04
EPI-WEEK:	04
DATE:	28 January, 2018

HIGHLIGHTS

- **In the reporting Week 04 (January 22-28, 2018) fifteen new confirmed[ii] cases** and two deaths were recorded from five States Edo (6), Ondo (4), Delta (1) ,Imo(1) and Taraba (3)
- From 1st – 25th January 2018, a total of **297 suspected[i] cases**, and 22 deaths have been reported from 13 active[iv] States- (Edo, Ondo, Bauchi, Nasarawa, Ebonyi, Anambra, Benue, Kogi, Imo, Plateau, Lagos, Taraba and Delta). *Figure 1*
- Since the beginning of 2018, 80 cases have been classified as: **77 confirmed** cases, 3 probable cases with 21 deaths (18 in confirmed and 3 in probable) -*Table 1*
- Case Fatality Rate in confirmed and probable cases is 27.6% and 7.4% for all cases (including probable, confirmed and suspected)
- **Ten Health Care workers have been affected in four states** –Ebonyi (7), Nasarawa (1) Kogi (1) and Benue (1) with four deaths in Ebonyi (3) and Kogi (1)
- NCDC staff and NFELTP residents (National RRT) deployed to Ebonyi, Ondo and Edo states to support the States
- Irrua Specialist Hospital has 43 cases on admission this weekend. FMC Owo has 18 isolation beds, all occupied. Colleagues in Irrua are also providing clinical management advise for other hospitals
- A total of 415 contacts have been identified and are currently under follow up
- NCDC supplied Irrua and Owo tents and beds this weekend for their surge capacity
- NCDC in collaborating with ALIMA in Edo and Ondo States for assessment of isolation Centres
- National Lassa fever Emergency Operations Centre(EOC) continues to coordinate the response
- Letter of notification of Lassa fever EOC Activation sent to 36 states and FCT
- Setup of 24 hours Lassa fever case management helpdesk -**09062654453**

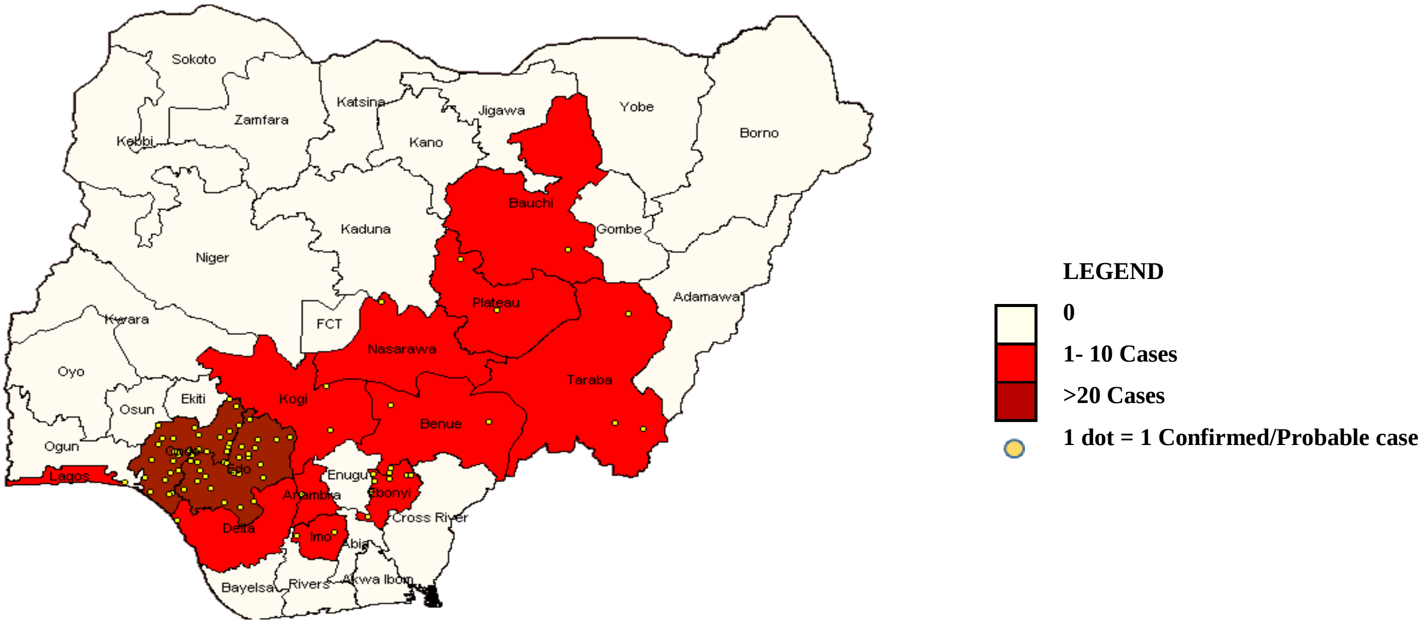

Figure 1. Distribution of Confirmed Lassa Fever cases in Nigeria as at 28th January, 2018

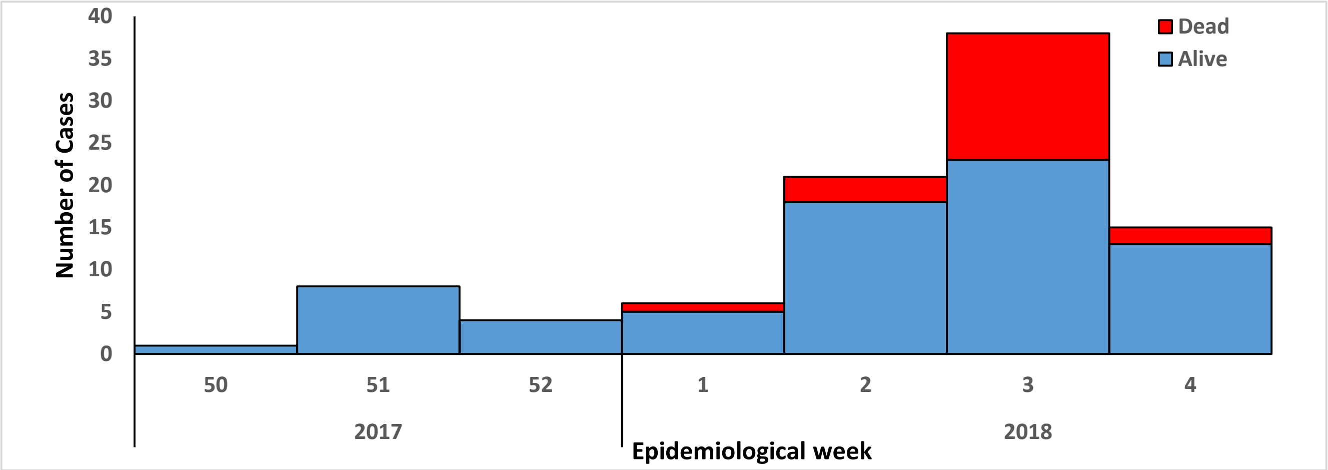

Figure 2. Confirmed and Probable Cases of Lassa fever by epidemiological week in Nigeria – Dec 2017 week 50 – 4 Jan 2018 (N = 80)

---

[i]*Suspected case describes any individual presenting with one or more of the following: malaise, fever, headache, sore throat, cough, nausea, vomiting, diarrhoea, myalgia, chest pain, hearing loss and either a. History of contact with excreta or urine of rodents b. History of contact with a probable or confirmed Lassa fever case within a period of 21 days of onset of symptoms OR Any person with inexplicable bleeding/hemorrhagia.*

[ii]*Any suspected case with laboratory confirmation (positive IgM antibody, PCR or virus isolation).*

[i]*Any suspected case (see definition above) but who died without collection of specimen for laboratory testing*

[iii]*Any suspected case (see definition above) but who died without collection of specimen for laboratory testing*

[iv] *"Active" means where there has been at least one confirmed case, and contacts within 21 days post exposure*

TITLE:	2018 LASSA FEVER OUTBREAK IN NIGERIA
SERIAL NUMBER:	05
EPI-WEEK:	05
DATE:	04 February, 2018

HIGHLIGHTS

- **In the reporting Week 05 (January 29-February 04, 2018) seventeen new confirmed[ii] cases** were recorded from Six States Edo (3), Ondo (5), Plateau (2), Anambra(1), Delta(2) Osun(1) Rivers (1) FCT(1) and Gombe(1) with nine new deaths in confirmed from eight states Ondo (2), Plateau (1), Anambra (1), Delta (1), Osun (1) Rivers (1), FCT (1) and Gombe (1)
- From 1st January to 4th February 2018, a total of 449 **suspected[i] cases**, and 43 deaths have been reported from 17 active[iv] States- (Edo, Ondo, Bauchi, Nasarawa, Ebonyi, Anambra, Benue, Kogi, Imo, Plateau, Lagos, Taraba, Delta, Osun, Rivers, FCT and Gombe). *Figure 1*
- Since the onset of the 2018 outbreak, 135 cases have been classified as: **132 confirmed** cases, 3 probable cases with 40 deaths (37 in Lab confirmed and 3 in probable) -*Table 1*
- Case Fatality Rate in confirmed and probable cases is 29.6% and 9.6% for all cases (including probable, confirmed and suspected)
- **Eleven Health Care workers have been affected in five states** –Ebonyi (7), Nasarawa (1) Kogi (1) Benue (1) and Ondo (1) with four deaths in Ebonyi (3) and Kogi (1)
- Predominant age group affected is age group 30-50 (Median Age = 32) *Figure3*
- The male to female ratio for confirmed cases is 2:1
- 75% of all confirmed cases are from Edo (42%) and Ondo (33%) states
- NCDC staff and NFELTP residents (National RRT) deployed to Ebonyi, Ondo and Edo states to support the States, additional team deployed to Ondo
- Irrua Specialist Hospital has 28 cases on admission this weekend. FMC Owo has 19 isolation beds, all occupied. Colleagues in Irrua are also providing clinical management advice for other hospitals
- A total of 858 contacts have been identified and 828 are currently under follow up
- NCDC is collaborating with ALIMA and MSF in Edo, Ondo and Anambra states to support case management
- NCDC deployed NFELTP Residents/Graduates in four Benin Republic border states (Kebbi, Kwara, Niger and Oyo) for enhanced surveillance activities
- NCDC in collaboration with ALIMA and AFENET supplied medicines and other commodities to States, treatment Centres and Military barracks in eight states
- National Lassa fever multi-partner multi-agency Emergency Operations Centre(EOC) continues to coordinate the response activities at all levels

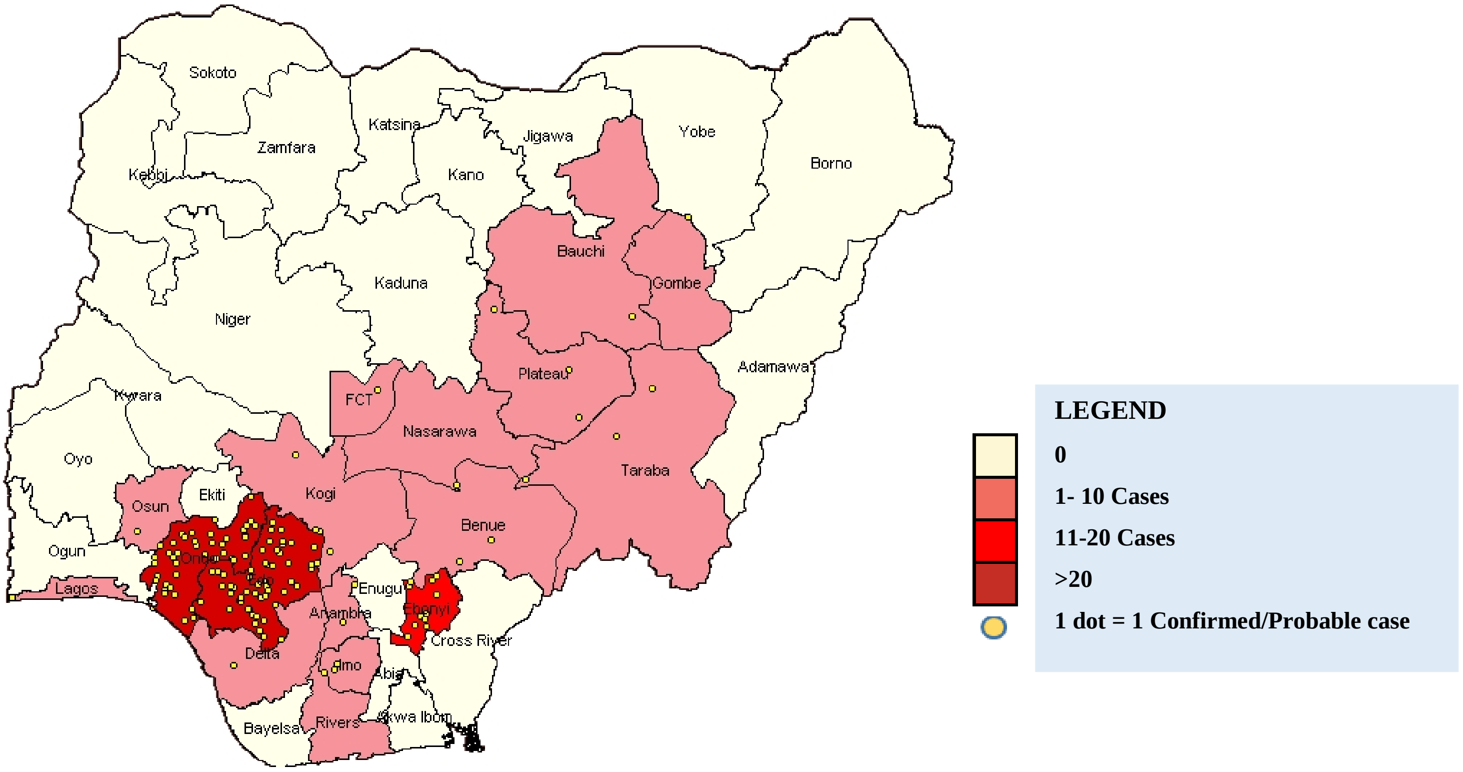

Figure 1. Distribution of Confirmed Lassa Fever cases in Nigeria as at 4th February, 2018

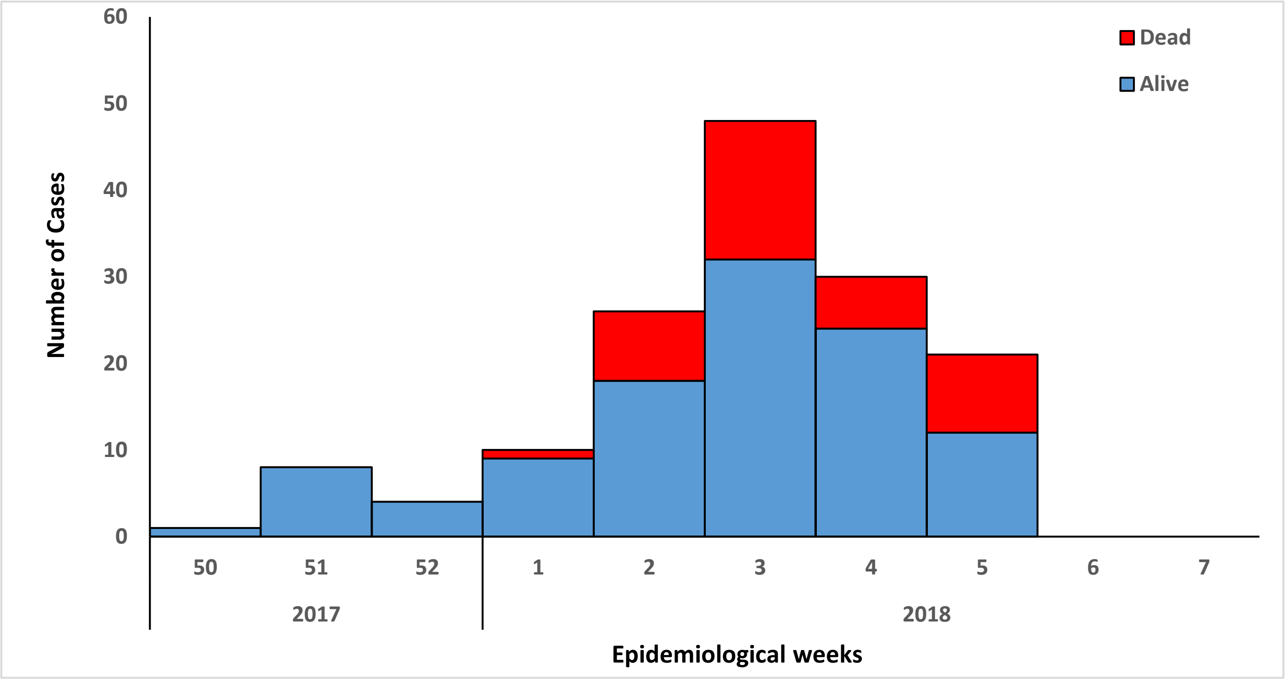

Figure 2. Confirmed and Probable Cases of Lassa fever by epidemiological week in Nigeria – Dec 2017 week 50 – 4 Jan 2018 (N = 135)

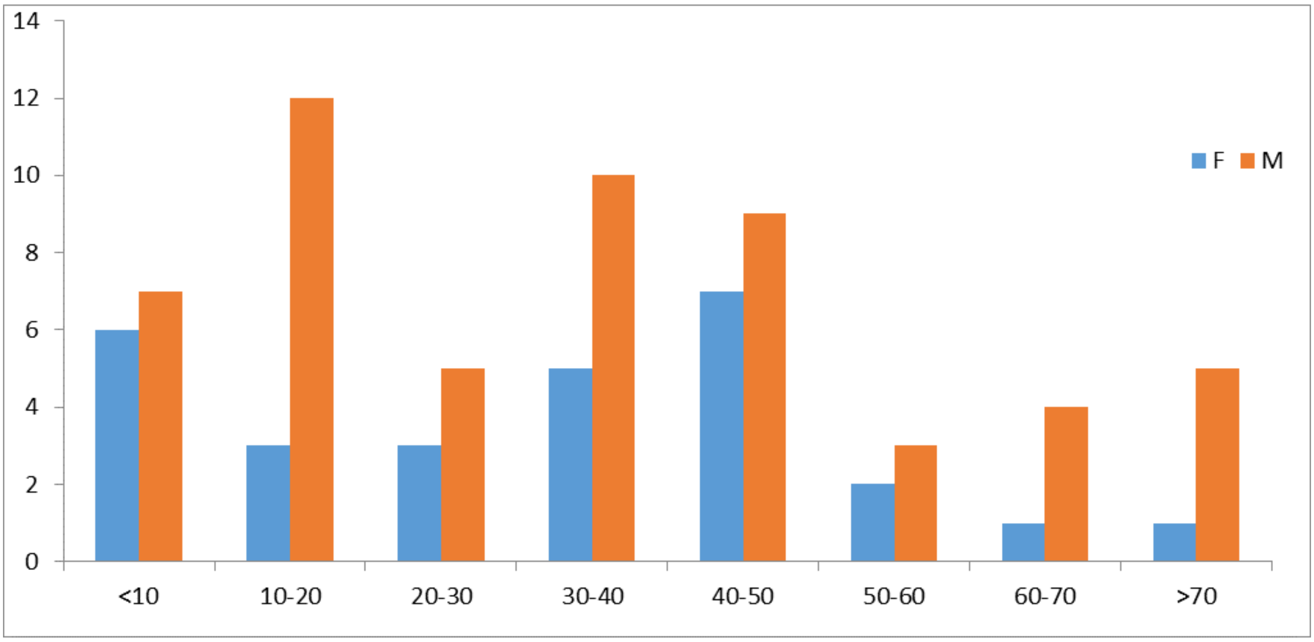

Figure 3. Age –Sex distribution of Confirmed Lassa fever cases in Nigeria as at 4th February 2018

---

[i]*Suspected case describes any individual presenting with one or more of the following: malaise, fever, headache, sore throat, cough, nausea, vomiting, diarrhoea, myalgia, chest pain, hearing loss and either a. History of contact with excreta or urine of rodents b. History of contact with a probable or confirmed Lassa fever case within a period of 21 days of onset of symptoms OR Any person with inexplicable bleeding/hemorrhagia.*

[ii]*Any suspected case with laboratory confirmation (positive IgM antibody, PCR or virus isolation)*

[iii]*Any suspected case (see definition above) who died without collection of specimen for laboratory testing*

[iv] *"Active" means where there has been at least one confirmed case, and contacts within 21 days post exposure*

TITLE:	2018 LASSA FEVER OUTBREAK IN NIGERIA
SERIAL NUMBER:	06
EPI-WEEK:	06
DATE:	11 February, 2018

HIGHLIGHTS

- **In the reporting Week 06 (February 05-11,2018) nineteen new confirmed[ii] cases** were recorded from Seven States Edo (3), Ondo (7), Bauchi (2), Nasarawa(1), Ebonyi(2) Kogi(2) and Taraba(2) with six new deaths in confirmed cases from four states Ondo (2), Edo(1), Bauchi (2), and Nasarawa (1)
- From 1st January to 11th February 2018, a total of 615 **suspected[i] cases**, and 57 deaths have been reported from 17 active[iv] States- (Edo, Ondo, Bauchi, Nasarawa, Ebonyi, Anambra, Benue, Kogi, Imo, Plateau, Lagos, Taraba, Delta, Osun, Rivers, FCT and Gombe). *Figure 1*
- Since the onset of the 2018 outbreak, 197 cases have been classified as: **193 confirmed** cases, 4 probable cases with 47 deaths (43 in Lab confirmed and 4 in probable)
- Case Fatality Rate in confirmed and probable cases is 23.9% and 9.3% for all cases (including probable, confirmed and suspected)
- **Fourteen Health Care workers have been affected in six states** –Ebonyi (7), Nasarawa (1), Kogi (1), Benue (1), Ondo (1) and Edo (3) with four deaths in Ebonyi (3) and Kogi (1)
- Predominant age group affected is age group 30-50 (Median Age = 32) *Figure 4*
- The male to female ratio for confirmed cases is 2:1
- 75% of all confirmed cases are from Edo (45%) and Ondo (30%) states
- NCDC staff and NFELTP residents (National RRT) deployed to Ebonyi, Ondo and Edo states to support the States
- Irrua Specialist Hospital has 28 cases on admission this weekend. FMC Owo has 19 isolation beds, all occupied. Colleagues in Irrua are also providing clinical management advice for other hospitals
- A total of 1641 contacts have been identified from 11 states and 1388 are currently under follow up
- NCDC is collaborating with ALIMA and MSF in Edo, Ondo and Anambra States to support case management
- NCDC deployed NFELTP Residents/Graduates in four Benin Republic border states (Kebbi, Kwara, Niger and Oyo) for enhanced surveillance activities
- National Lassa fever multi-partner multi-agency Emergency Operations Centre(EOC) continues to coordinate the response activities at all levels
- NCDC is now being supported and strengthened by WHO team from AFRO, Headquarters and Country office.

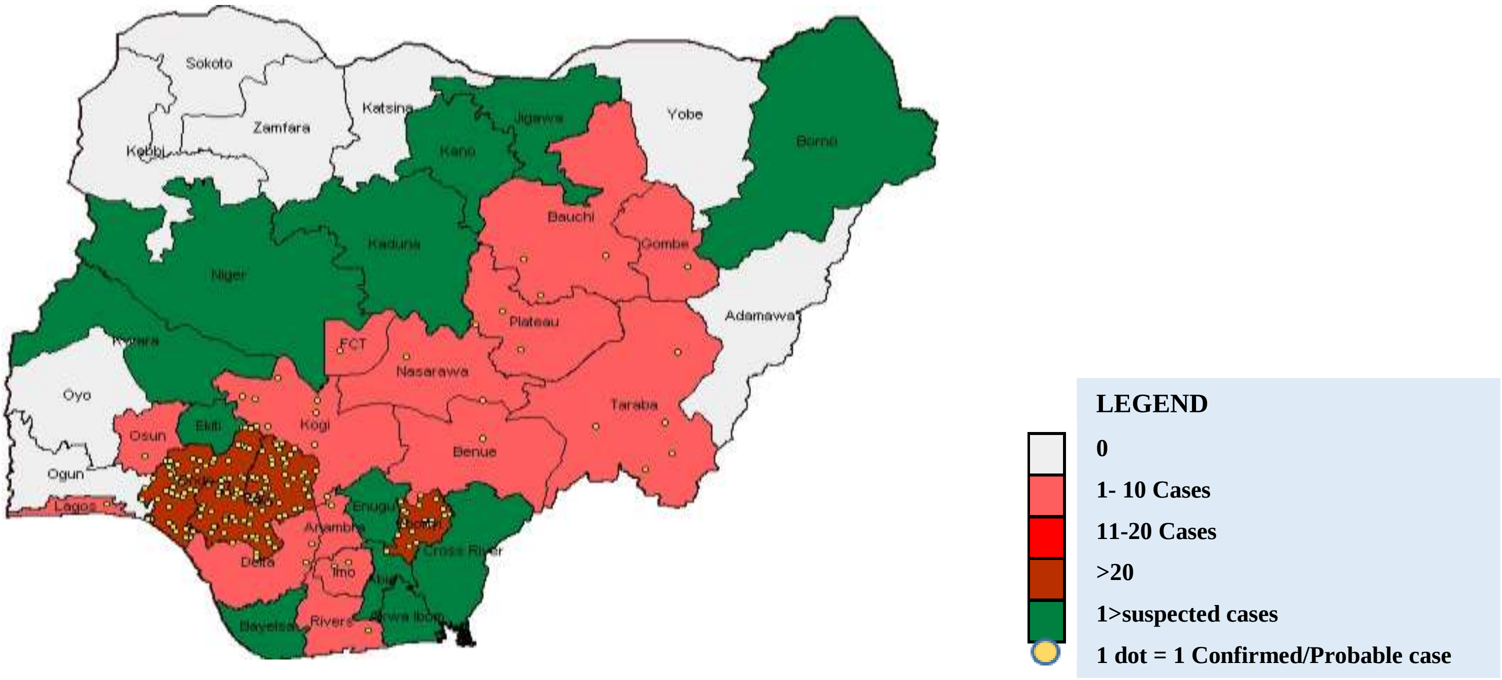

Figure 1. Distribution of Confirmed and Probable Lassa Fever cases in Nigeria as at 11th February, 2018

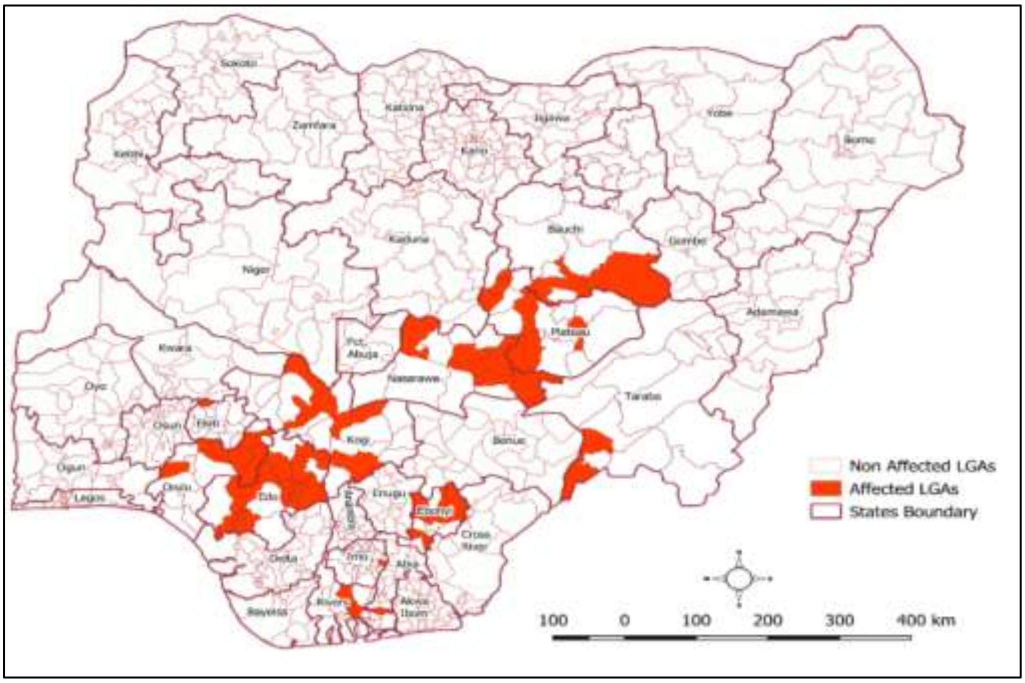

Figure 2. Distribution of Confirmed and Probable Lassa Fever cases in Nigeria by LGA as at 11th February, 2018

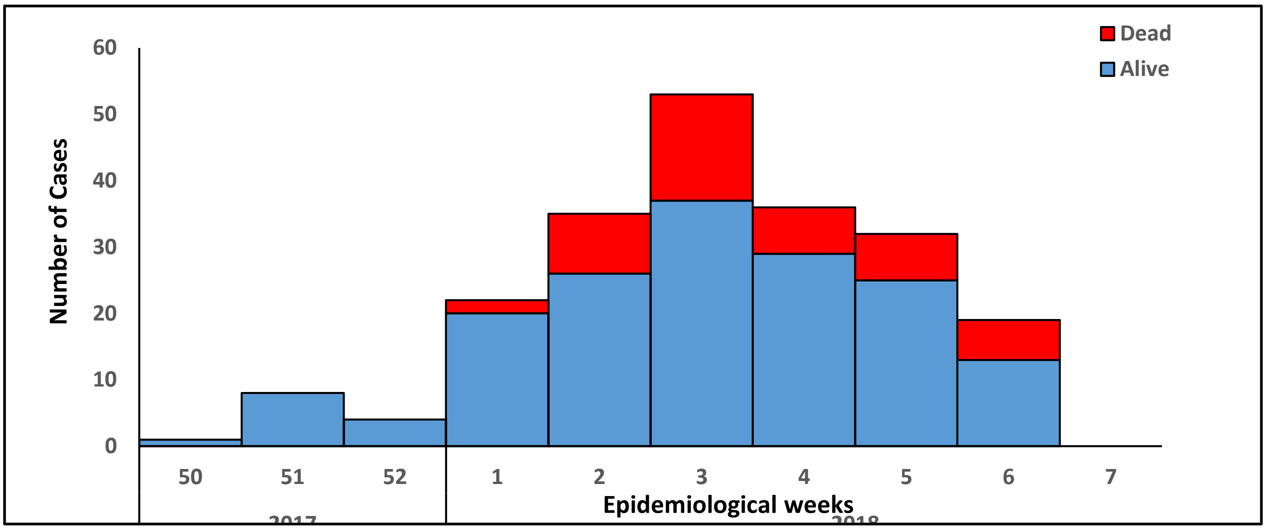

Figure 3. Confirmed and Probable Cases of Lassa fever by epidemiological week in Nigeria Dec 2017 week 50 – 11 Feb 2018 (N = 197)

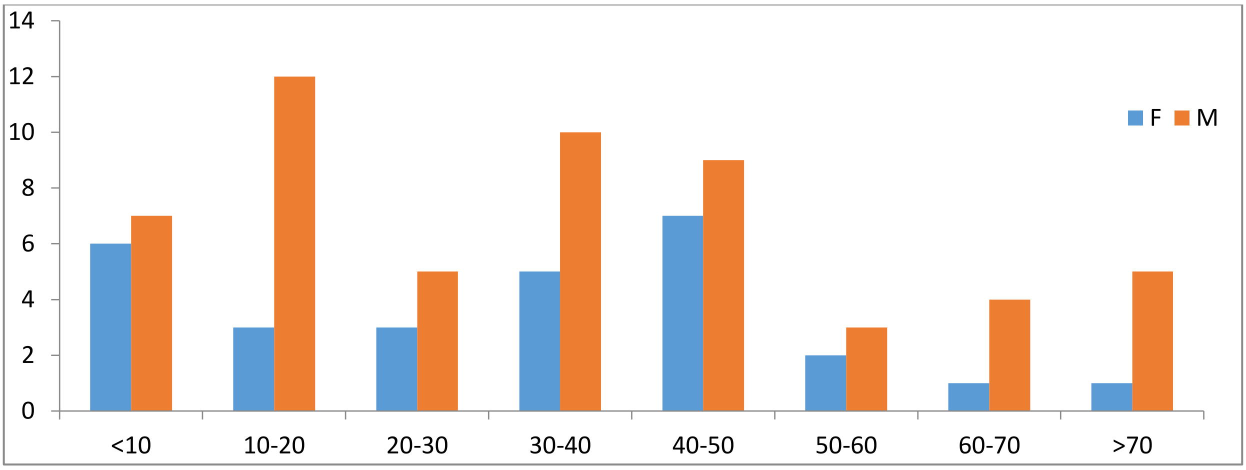

Figure 4. Age –Sex distribution of Confirmed Lassa fever cases in Nigeria as at 11th February 2018

---

[i] *Suspected case describes any individual presenting with one or more of the following: malaise, fever, headache, sore throat, cough, nausea, vomiting, diarrhoea, myalgia, chest pain, hearing loss and either a. History of contact with excreta or urine of rodents b. History of contact with a probable or confirmed Lassa fever case within a period of 21 days of onset of symptoms OR Any person with inexplicable bleeding/hemorrhagia.*

[ii] *Any suspected case with laboratory confirmation (positive IgM antibody, PCR or virus isolation)*

[iii] *Any suspected case (see definition above) who died without collection of specimen for laboratory testing*

[iv] *"Active" means where there has been at least one confirmed case, and contacts within 21 days post exposure*

TITLE:	2018 LASSA FEVER OUTBREAK IN NIGERIA
SERIAL NUMBER:	07
EPI-WEEK:	07
DATE:	18 February 2018

HIGHLIGHTS

- **In the reporting Week 07 (February 12-18,2018) sixty eight new confirmed[il] cases** were recorded from seven States Edo (35), Ondo (19), Bauchi (1), Ebonyi (7), Anambra (4), Imo(1) and FCT (1) with four new deaths in confirmed cases from two states Ondo (2), and Bauchi (2)
- From 1st January to 18th February 2018, a total of 913 **suspected[i] cases**, and 73 deaths have been reported from 17 active[iv] States- (Edo, Ondo, Bauchi, Nasarawa, Ebonyi, Anambra, Benue, Kogi, Imo, Plateau, Lagos, Taraba, Delta, Osun, Rivers, FCT, and Gombe) - *Figure 1*
- Since the onset of the 2018, 277 cases have been classified as: **272 confirmed** cases, 5 probable cases with 59 deaths (54 in Lab confirmed and 5 in probable) -*Table 1*
- Case Fatality Rate in confirmed and probable cases is 21%
- **Fourteen Health Care workers have been affected in six states** –Ebonyi (7), Nasarawa (1), Kogi (1), Benue (1), Ondo (1) and Edo (3) with four deaths in Ebonyi (3) and Kogi (1)
- Predominant age group affected is age group 30-50 (Median Age = 32) - *Figure 4*
- The male to female ratio for confirmed cases is 2:1
- 74% of all confirmed cases are from Edo (45%) and Ondo (29%) states
- National RRT team (NCDC staff and NFELTP residents) batch A replaced with batch B to continue response support in Ebonyi, Ondo and Edo States
- **Irrua Specialist Hospital has 39 cases on admission this weekend. FMC Owo has 29 isolation beds, all occupied.**
- A total of 2351 contacts have been identified from 17 active states and 1747 are currently being followed up
- Joint NCDC and WHO team on high level visit to Edo, Ondo and Ebonyi states
- NCDC is collaborating with ALIMA and MSF in Edo, Ondo and Anambra States to support case management
- NCDC deployed teams to four Benin Republic border states (Kebbi, Kwara, Niger and Oyo) for enhanced surveillance activities
- National Lassa fever multi-partner multi-agency Emergency Operations Centre(EOC) continues to coordinate the response activities at all levels

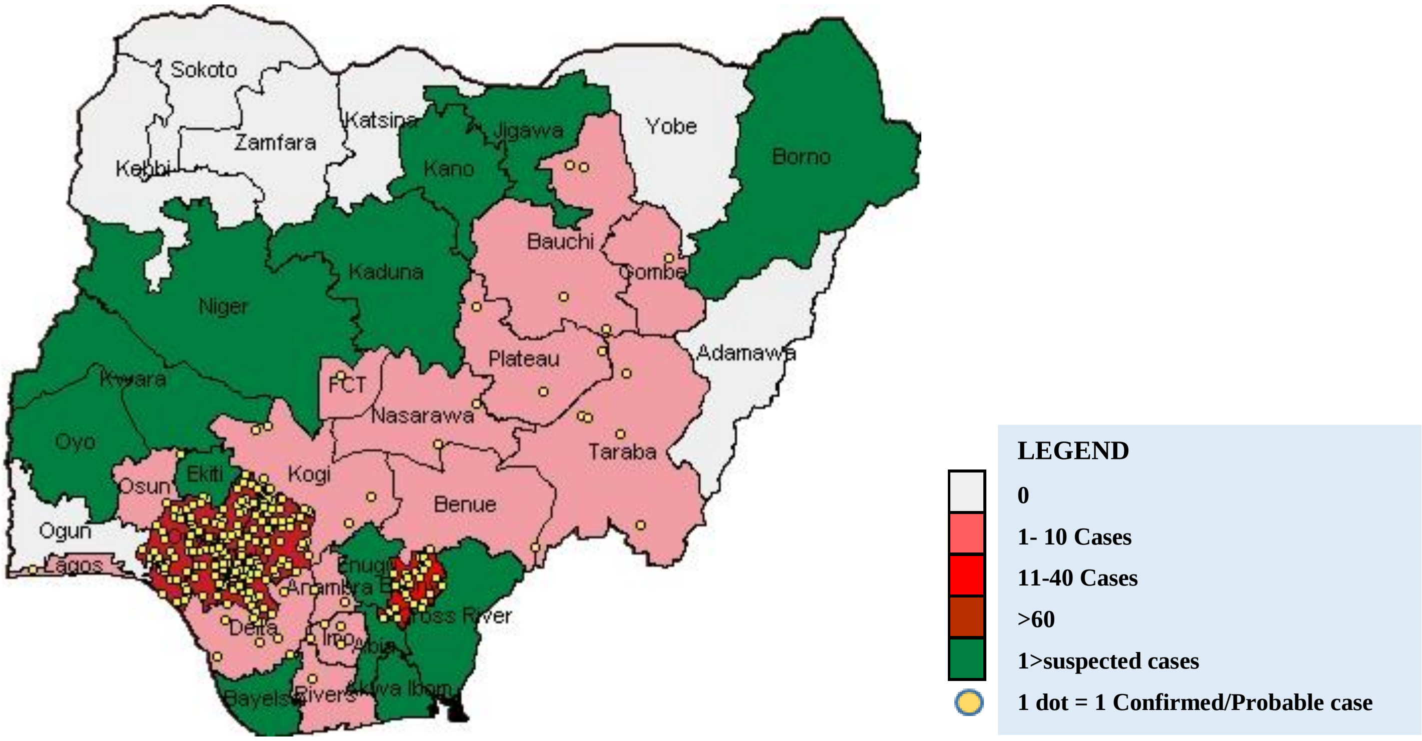

Figure 1. Distribution of Confirmed and Probable Lassa Fever cases in Nigeria as at 18th February, 2018

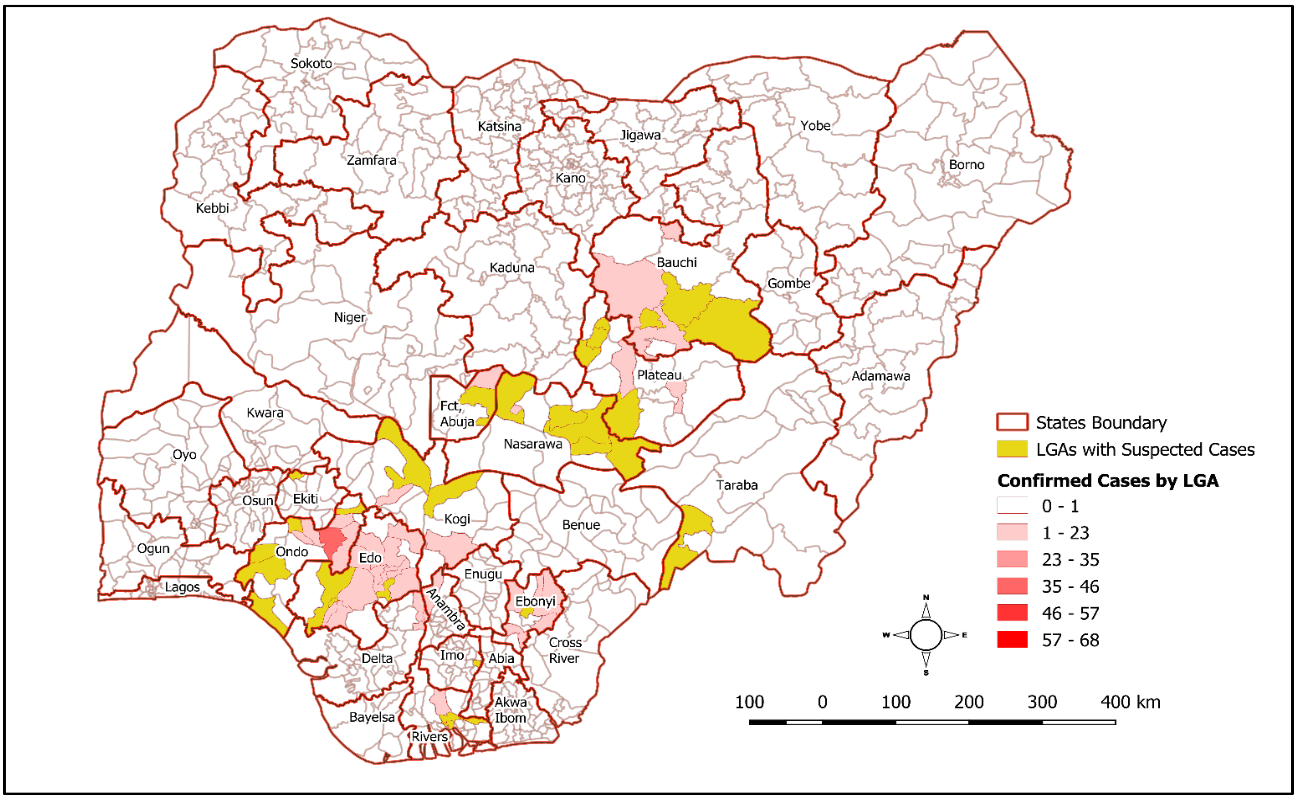

Figure 2. Distribution of Confirmed, Probable and Suspected Lassa Fever cases in Nigeria by LGA as at 18th February, 2018

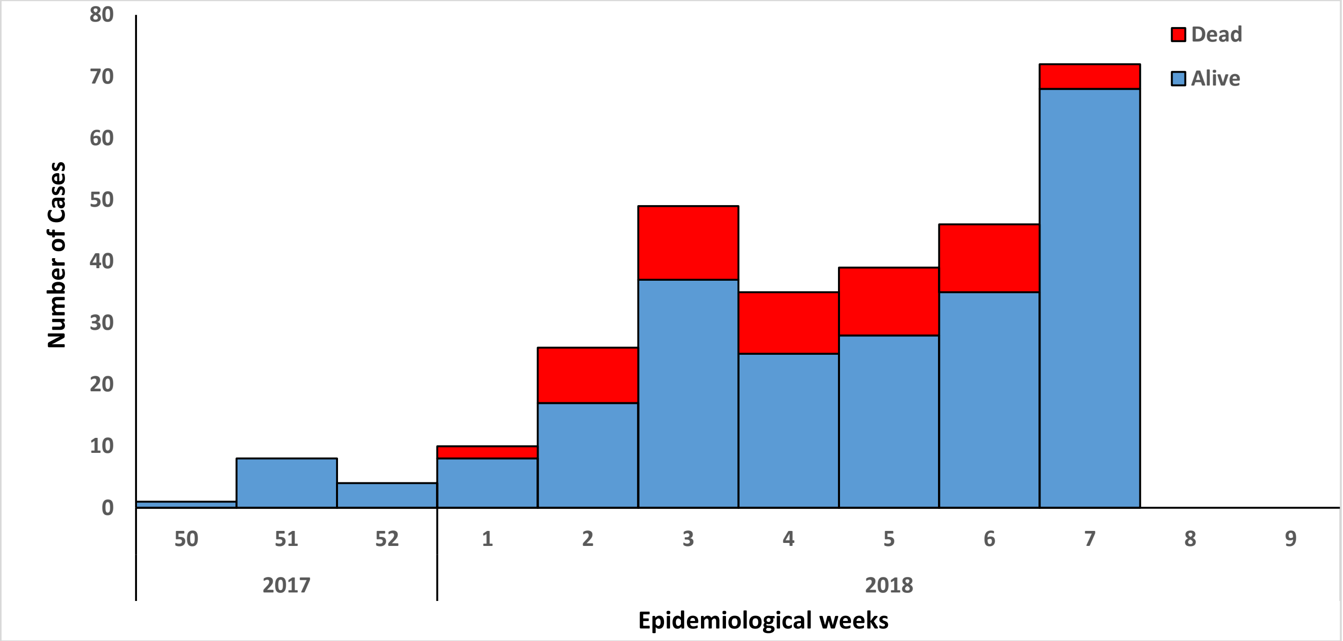

Figure 3. Confirmed and Probable Cases of Lassa fever by epidemiological week in Nigeria Dec 2017 week 50 – 18th February 2018 (N = 277)

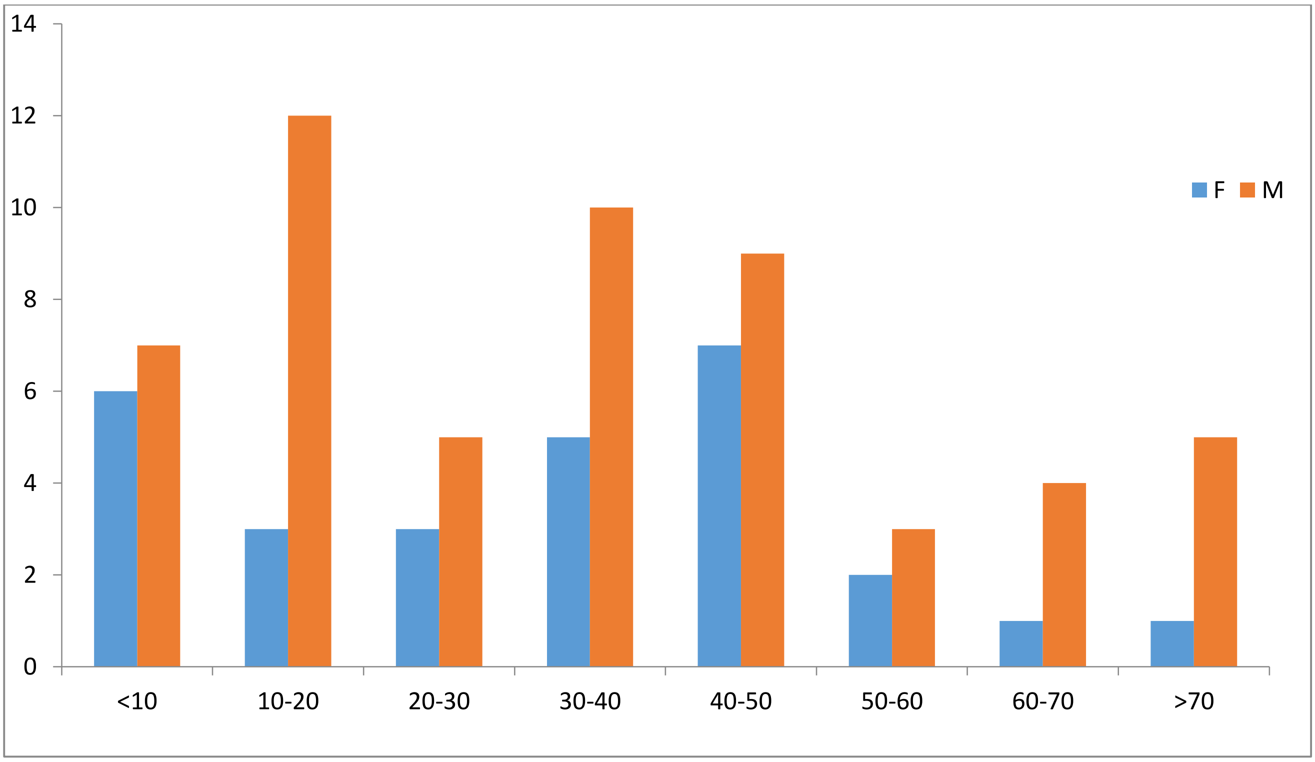

Figure 4. Age –Sex distribution of Confirmed Lassa fever cases in Nigeria as at 18th February 2018

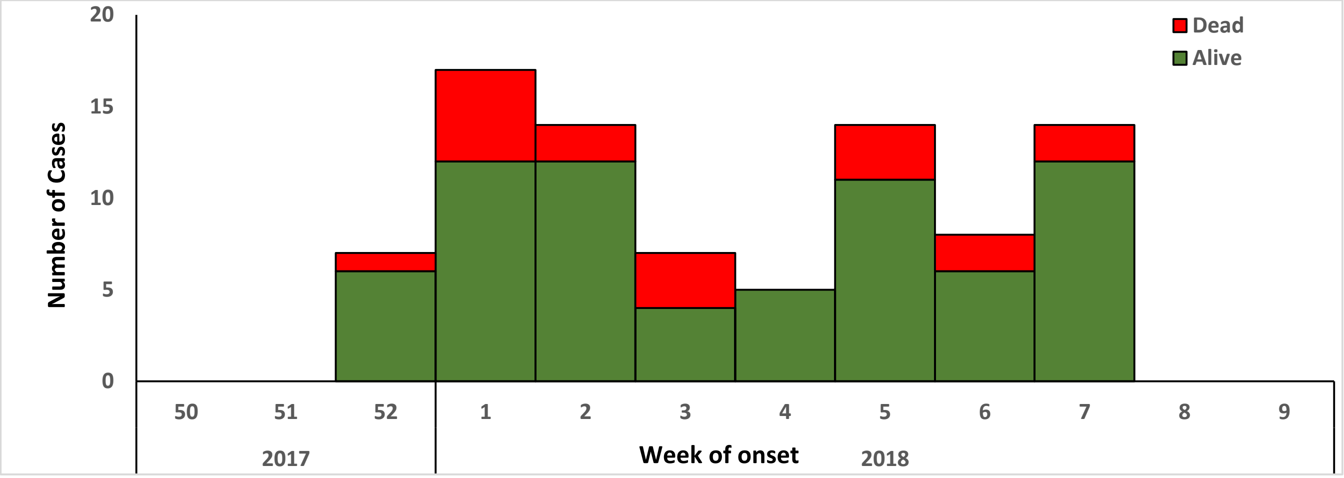

Epicurve of Lassa fever confirmed cases in Ondo State week 49 2017 to 18th February 2018

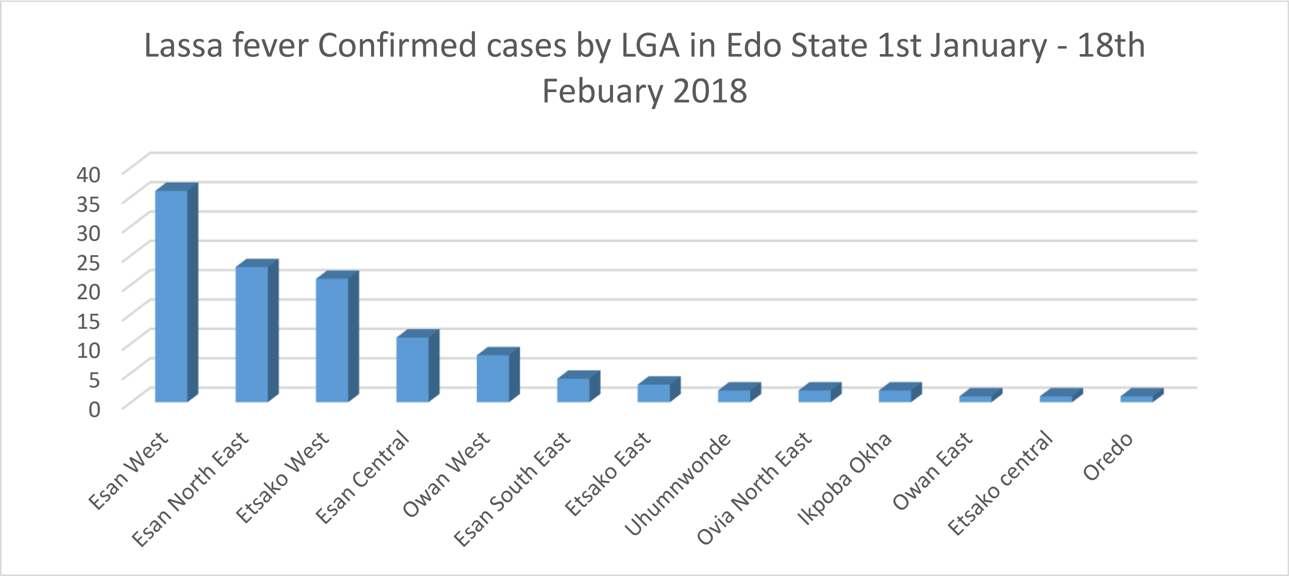

Figure 5: Lassa fever confirmed cases in Edo State week 1st January to 18th February 2018

---

[i] *Suspected case describes any individual presenting with one or more of the following: malaise, fever, headache, sore throat, cough, nausea, vomiting, diarrhoea, myalgia, chest pain, hearing loss and either a. History of contact with excreta or urine of rodents b. History of contact with a probable or confirmed Lassa fever case within a period of 21 days of onset of symptoms OR Any person with inexplicable bleeding/hemorrhagia.*

[ii] *Any suspected case with laboratory confirmation (positive IgM antibody, PCR or virus isolation)*

[iii] *Any suspected case (see definition above) who died without collection of specimen for laboratory testing*

[iv] *"Active" means where there has been at least one confirmed case, and contacts within 21 days post exposure*

SITUATION REPORT
Nigeria Centre For Disease Control (NCDC)

www.ncdc.gov.ng PLOT 800 EBITU UKIWE STREET, JABI ABUJA, NIGERIA. **TOLL FREE CALL:** 0800-970000-10. **E:** info@ncdc.gov.ng @NCDCgov

**TITLE:**	**2018 LASSA FEVER OUTBREAK IN NIGERIA**
**SERIAL NUMBER:**	**08**
**EPI-WEEK:**	**08**
**DATE:**	**25 February 2018**

HIGHLIGHTS

- **In the reporting Week 08 (February 19-25,2018) fifty four new confirmed[ii] cases** were recorded from eight States Edo (21), Ondo (9), Nasarawa (2), Ebonyi (18), Plateau(1),Kogi(1) Imo (1)and Ekiti (1) with ten new deaths in confirmed cases from five states Ondo (2), Edo (2), Plateau (1) Ekiti(1) and Ebonyi (4 and 2 probable deaths)
- From 1st January to 25th February 2018, a total of 1081 **suspected[i] cases**, and 90 deaths have been reported from 18 active[iv] States- (Edo, Ondo, Bauchi, Nasarawa, Ebonyi, Anambra, Benue, Kogi, Imo, Plateau, Lagos, Taraba, Delta, Osun, Rivers, FCT, Gombe and Ekiti) - *Figure 1*
- Since the onset of the 2018, 325 cases have been classified as: **317 confirmed** cases, 8 probable cases with 72 deaths (64 in Lab confirmed and 8 in probable)
- Case Fatality Rate in confirmed and probable cases is 22%
- **Fourteen Health Care workers have been affected in six states** –Ebonyi (7), Nasarawa (1), Kogi (1), Benue (1), Ondo (1) and Edo (3) with four deaths in Ebonyi (3) and Kogi (1)
- Predominant age group affected is age group 21-40 (Median Age = 34) - *Figure 5*
- The male to female ratio for confirmed cases is 2:1
- 69% of all confirmed cases are from Edo (43%) and Ondo (26%) states
- National RRT team (NCDC staff and NFELTP residents) batch B continues response support in Ebonyi, Ondo and Edo States
- **Irrua Specialist Hospital has 42 cases on admission this weekend. FMC Owo has 21 isolation beds, all occupied**
- A total of 2845 contacts have been identified from 18 active states and 1897 are currently being followed up
- WHO scaling up its support of the response at National and State levels
- NCDC is collaborating with ALIMA and MSF in Edo, Ondo and Anambra States to support case management
- NCDC deployed teams to four Benin Republic border states (Kebbi, Kwara, Niger and Oyo) for enhanced surveillance activities
- National Lassa fever multi-partner multi-agency Emergency Operations Centre(EOC) continues to coordinate the response activities at all levels

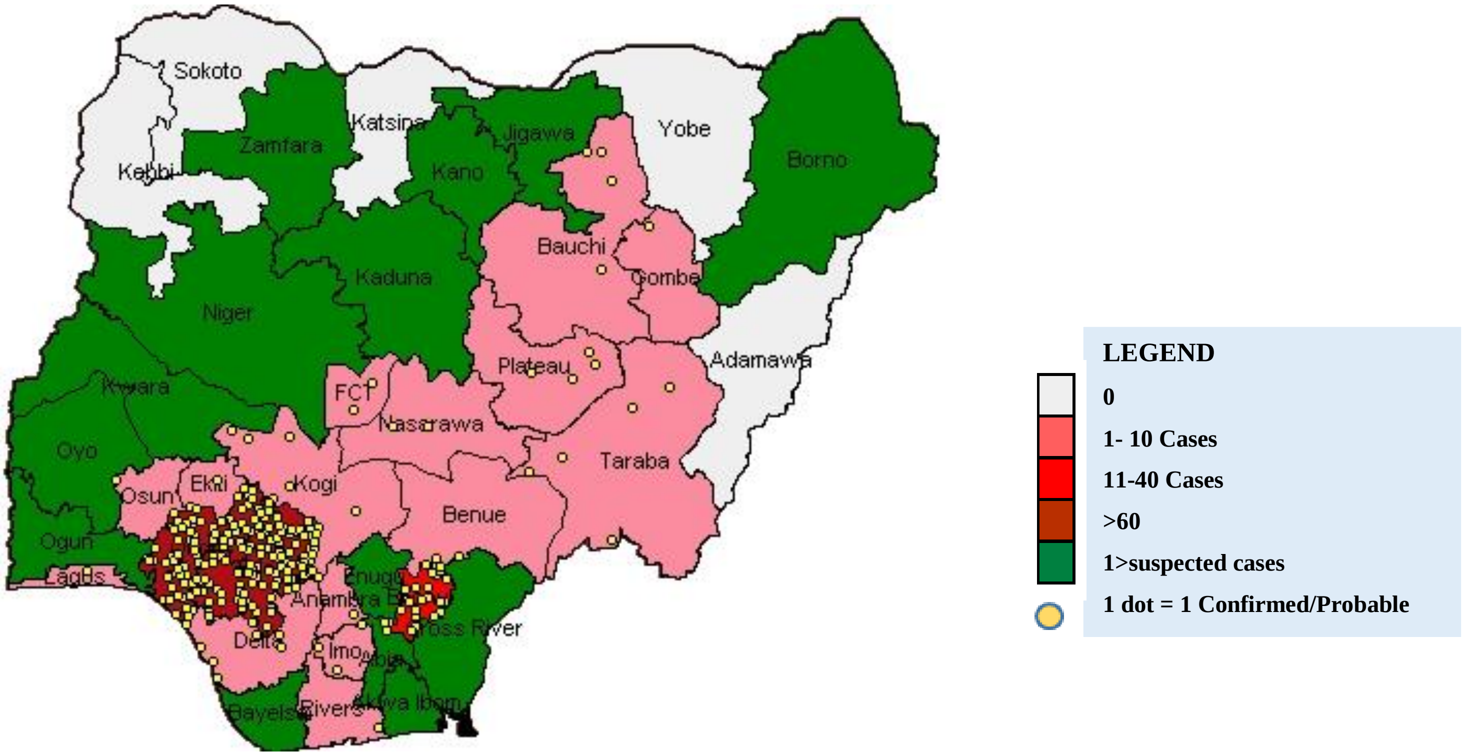

Figure 1. Distribution of Confirmed and Probable Lassa Fever cases in Nigeria as at 25th February, 2018

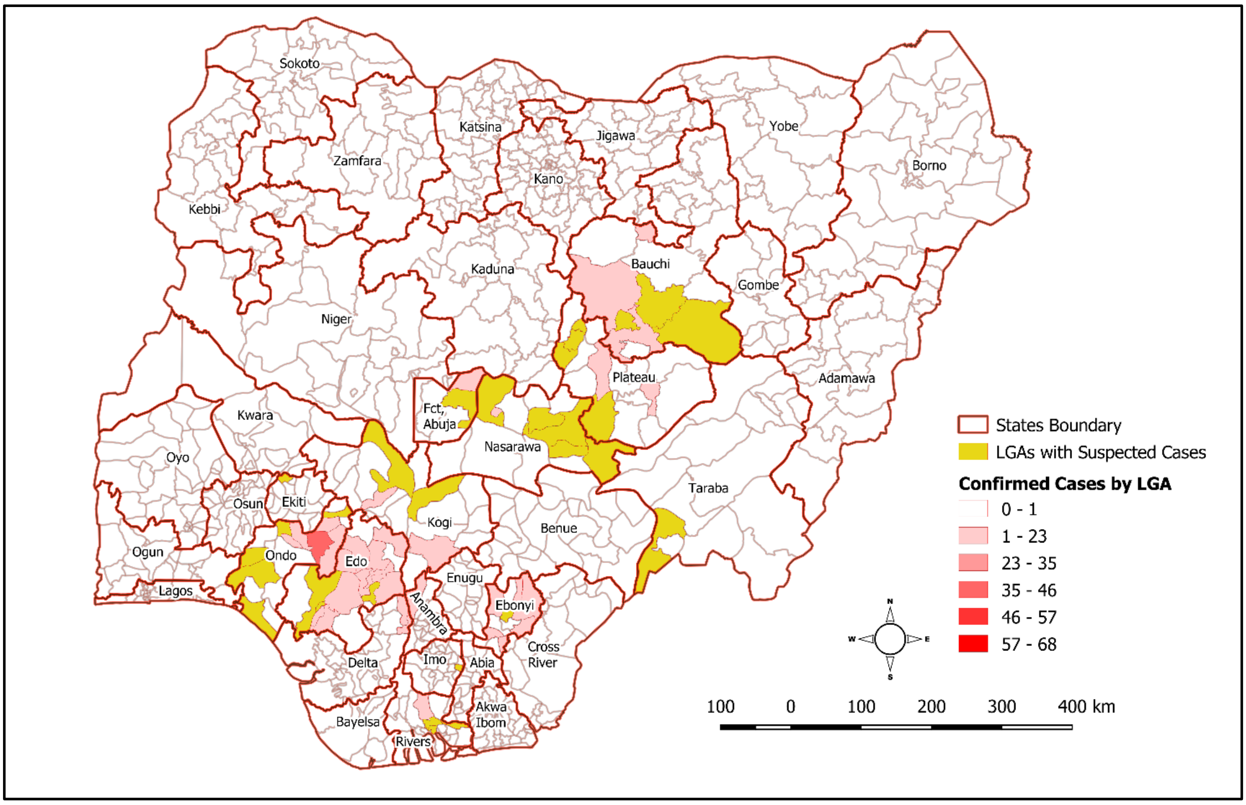

Figure 2. Distribution of Confirmed, Probable and Suspected Lassa Fever cases in Nigeria by LGA as at 25th February, 2018

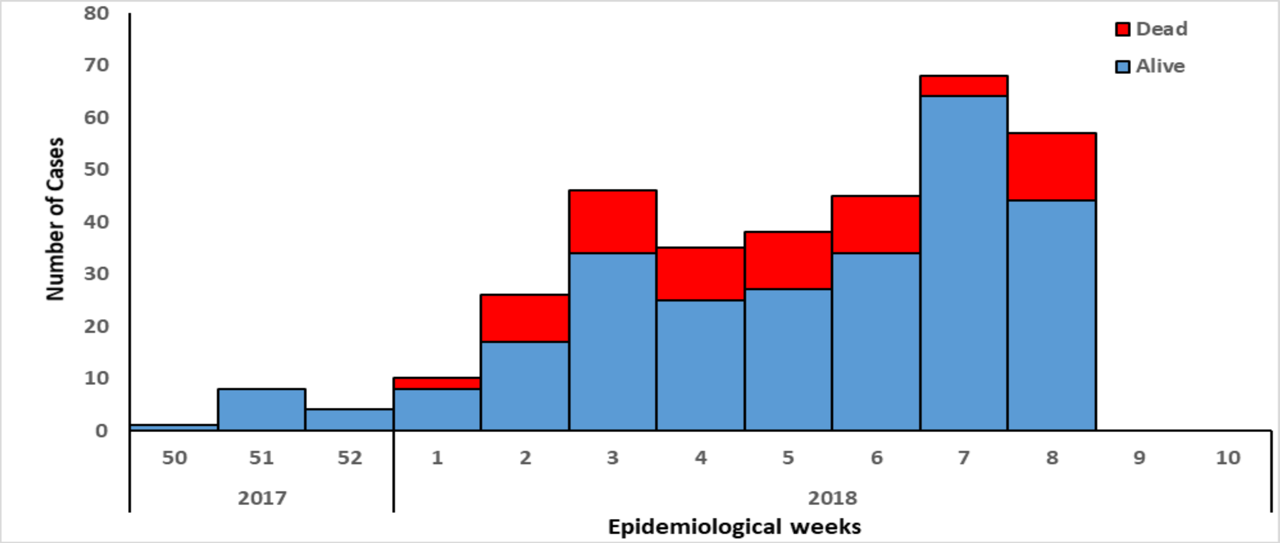

Figure 3. Confirmed (317) and Probable () Cases of Lassa fever by epidemiological week in Nigeria Dec 2017 week 50 – 18th February 2018 (N = 325)

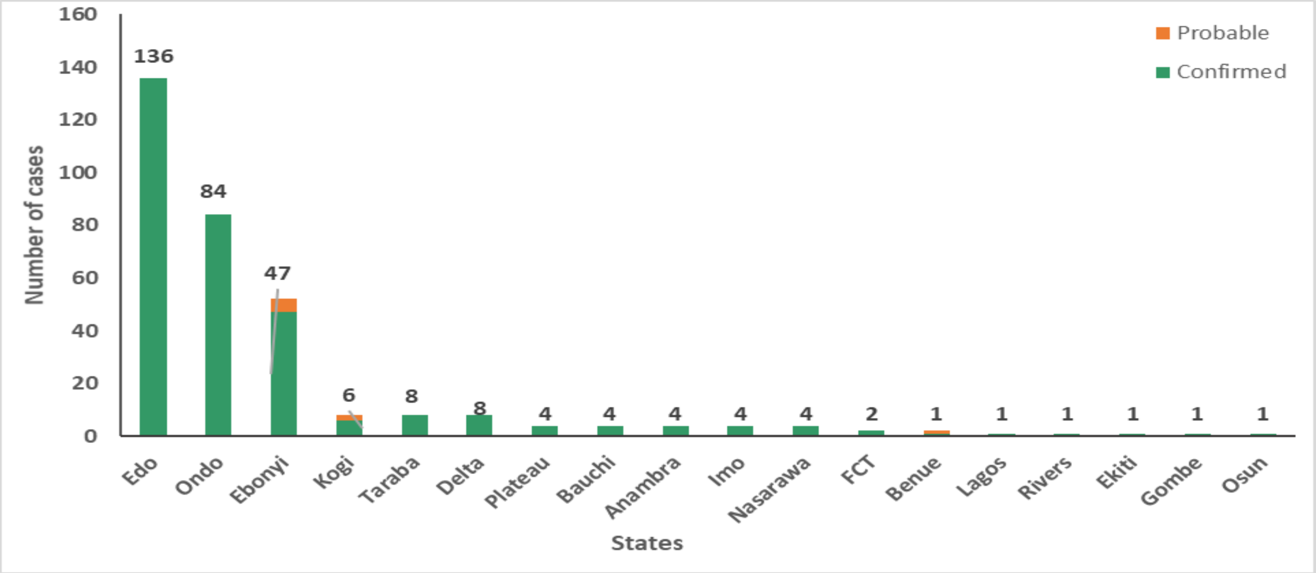

Figure 4. State distribution of Confirmed/Probable Lassa fever cases in Nigeria as at 25th February 2018

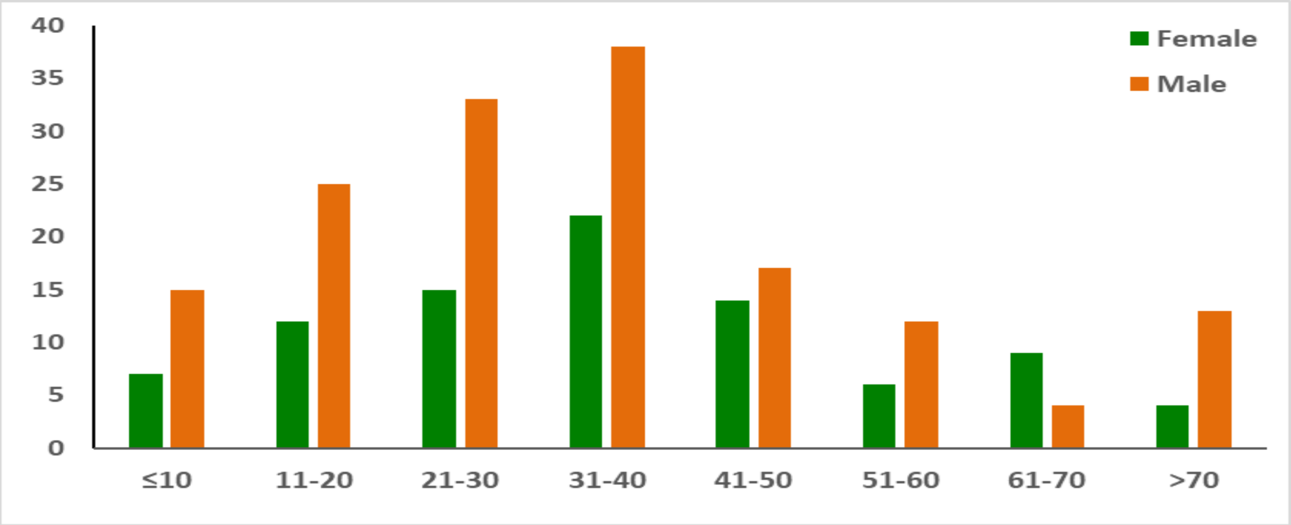

Figure 5. Age –Sex distribution of Confirmed Lassa fever cases in Nigeria as at 25th February 2018

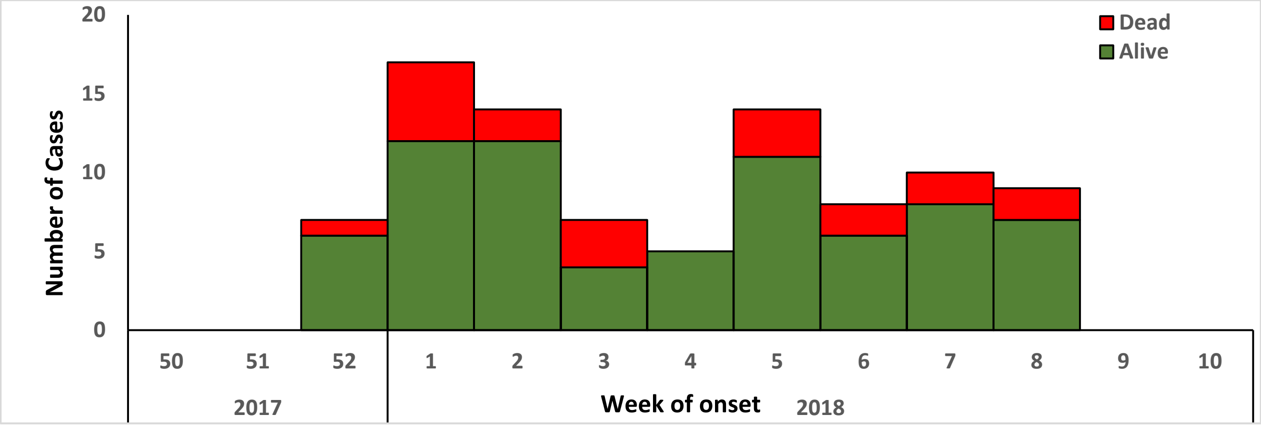

Figure 6: Epicurve of Lassa fever confirmed cases in Ondo State week 50 2017 to week 8 as at 25th February 2018

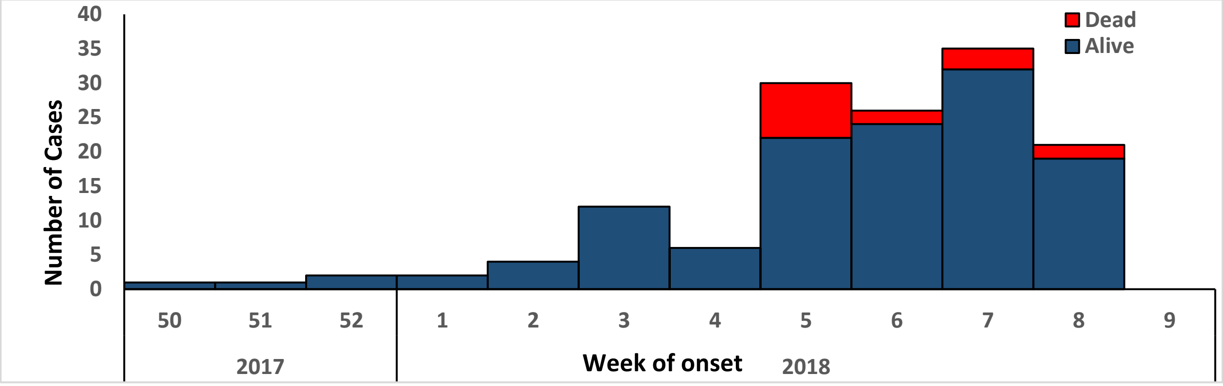

Figure 7: Epicurve of Lassa fever confirmed cases in Edo State week 50 2017 to week 8 as at 25th February 2018

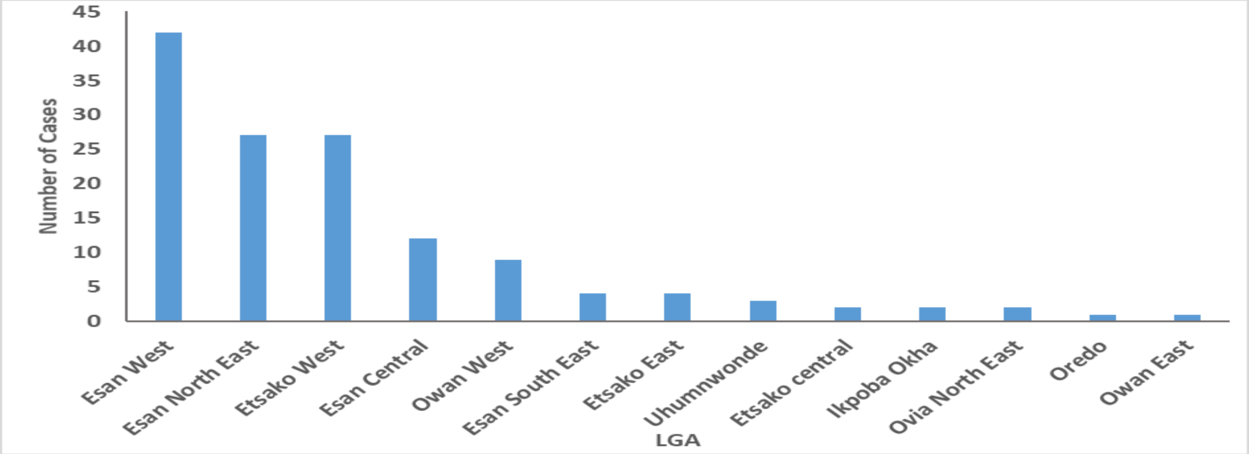

Figure 7: Lassa fever confirmed cases in Edo State by LGA 1st January to 25th February 2018

---

[i] *Suspected case describes any individual presenting with one or more of the following: malaise, fever, headache, sore throat, cough, nausea, vomiting, diarrhoea, myalgia, chest pain, hearing loss and either a. History of contact with excreta or urine of rodents b. History of contact with a probable or confirmed Lassa fever case within a period of 21 days of onset of symptoms OR Any person with inexplicable bleeding/hemorrhagia.*

[ii] *Any suspected case with laboratory confirmation (positive IgM antibody, PCR or virus isolation)*

[iii] *Any suspected case (see definition above) who died without collection of specimen for laboratory testing*

[iv] *"Active" means where there has been at least one confirmed case, and contacts within 21 days post exposure*

www.ncdc.gov.ng PLOT 800 EBITU UKIWE STREET, JABI ABUJA, NIGERIA. **TOLL FREE CALL:** 0800-970000-10. **E:** info@ncdc.gov.ng @NCDCgov

TITLE:	2018 LASSA FEVER OUTBREAK IN NIGERIA
SERIAL NUMBER:	09
EPI-WEEK:	09
DATE:	04 March 2018

HIGHLIGHTS

- **In the reporting Week 09 (February 26-March 4,2018) thirty five new confirmed**[ii] **cases** were recorded from five States Edo (19), Ondo (5), Bauchi (1), Ebonyi (9), and Plateau (1), with **seven new deaths** in confirmed cases from three states Ondo (2), Edo (2), and Ebonyi (3)
- From 1st January to 4th March 2018, a total of 1121 **suspected**[i] **cases. Of these, 353 are confirmed positive, 8 are probable, 723 are negative (not a case) and 37 are awaiting laboratory results (pending)**. 18 States are active[iv] (Edo, Ondo, Bauchi, Nasarawa, Ebonyi, Anambra, Benue, Kogi, Imo, Plateau, Lagos, Taraba, Delta, Osun, Rivers, FCT, Gombe and Ekiti) - *Table 1/ Figure 1*
- Since the onset of the 2018 outbreak, there have been 110 deaths: 78 in positive-confirmed cases, 8 in probable cases and 24 in negative cases. Case Fatality Rate in confirmed and probable cases is 23.8% -*Table 1*
- Two health workers were confirmed positive this week in Ebonyi State. Cumulatively, **sixteen health care workers have been affected in six states** –Ebonyi (9), Nasarawa (1), Kogi (1), Benue (1), Ondo (1) and Edo (3) with four deaths in Ebonyi (3) and Kogi (1)
- Predominant age-group affected is 21-40 years (Range: 9 months to 92 years, Median Age: 34 years) - *Figure 5*
- The male to female ratio for confirmed cases is 2:1
- 85% of all confirmed cases are from Edo (44%) Ondo (25%) and Ebonyi (16%)states
- **Cases currently on admission this weekend at Irrua Specialist Hospital (35), FMC Owo (18) and FETH Abakiliki (16) all isolation beds at the treatment facilities occupied.**
- National RRT team (NCDC staff and NFELTP residents) batch B continues response support in Ebonyi, Ondo and Edo States
- A total of 3126 contacts have been identified from 18 active states. Of these 1586 are currently being followed up, 1485 have completed 21 days follow up and **21** of the 47 symptomatic contacts have tested positive from 3 states (Edo-11, Ondo-7 and Ebonyi-3)
- WHO and NCDC has scaled up response at National and State levels
- Bernhard Nocht Institute for Tropical Medicine Germany is currently supporting ISTH, NRL and LUTH Laboratories with testing reagents
- NCDC distributed response commodities -PPEs, Ribavirin (injection and tablets), beds, body bags and hand sanitizers to FMC Owo, FETH Abakiliki, Niger and Ekiti states in the reporting week
- NCDC is collaborating with ALIMA and MSF in Edo, Ondo and Anambra States to support case management
- NCDC deployed teams to four Benin Republic border states (Kebbi, Kwara, Niger and Oyo) for enhanced surveillance activities
- National Lassa fever multi-partner multi-agency Emergency Operations Centre (EOC) continues to coordinate the response activities at all levels

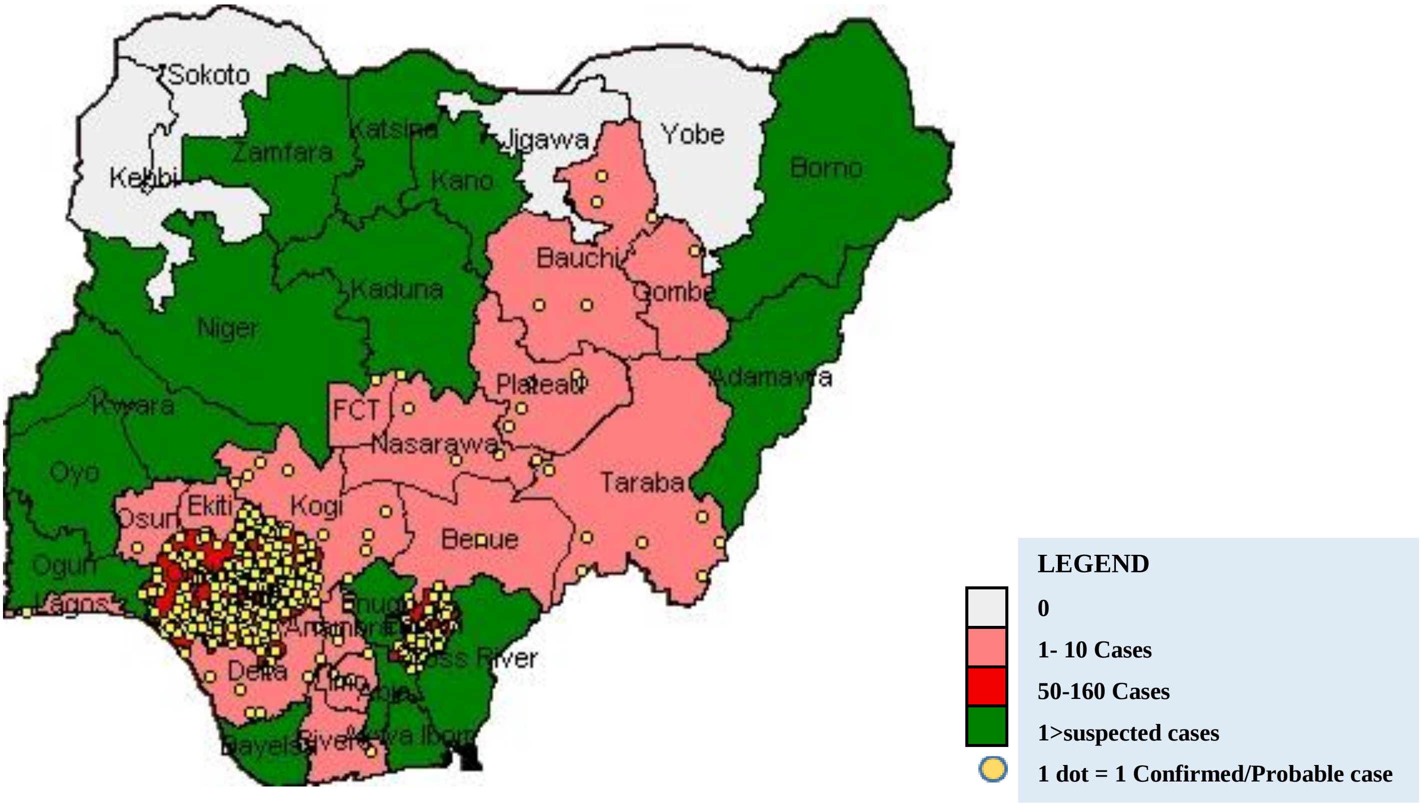

Figure 1. Distribution of Confirmed Lassa Fever cases in Nigeria as at 4th March, 2018

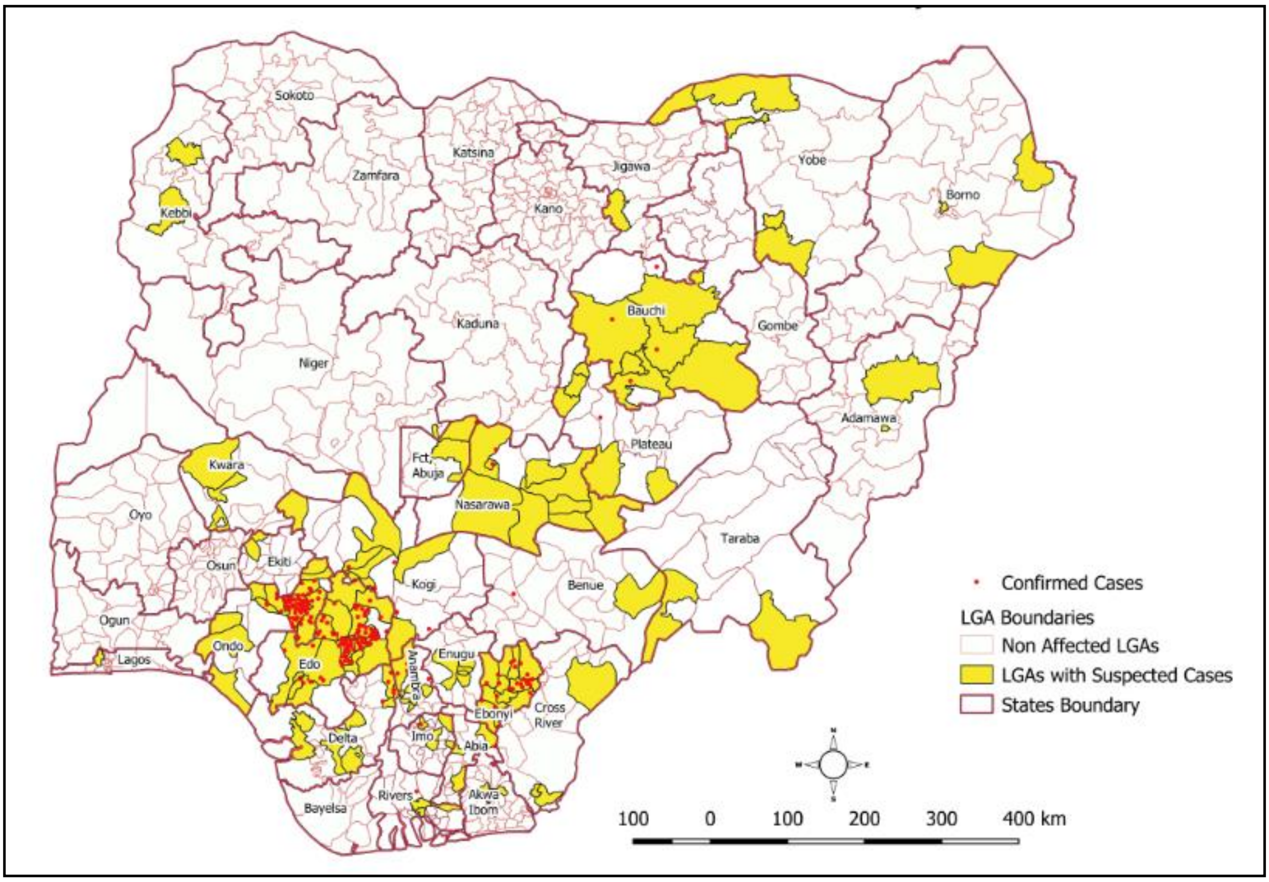

Figure 2. Distribution of Suspected and Confirmed Lassa Fever cases in Nigeria by LGA as at 4th March, 2018

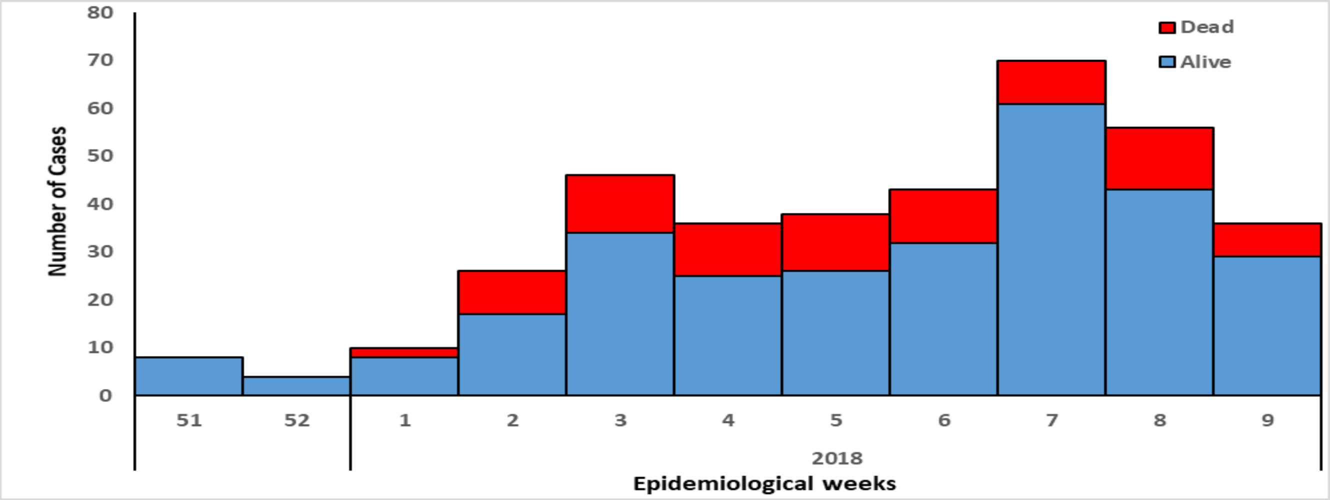

Figure 3. Confirmed (323) and Probable (8) Cases of Lassa fever by epidemiological week in Nigeria Dec 2017 week 51 – week 9 as at 4th March 2018

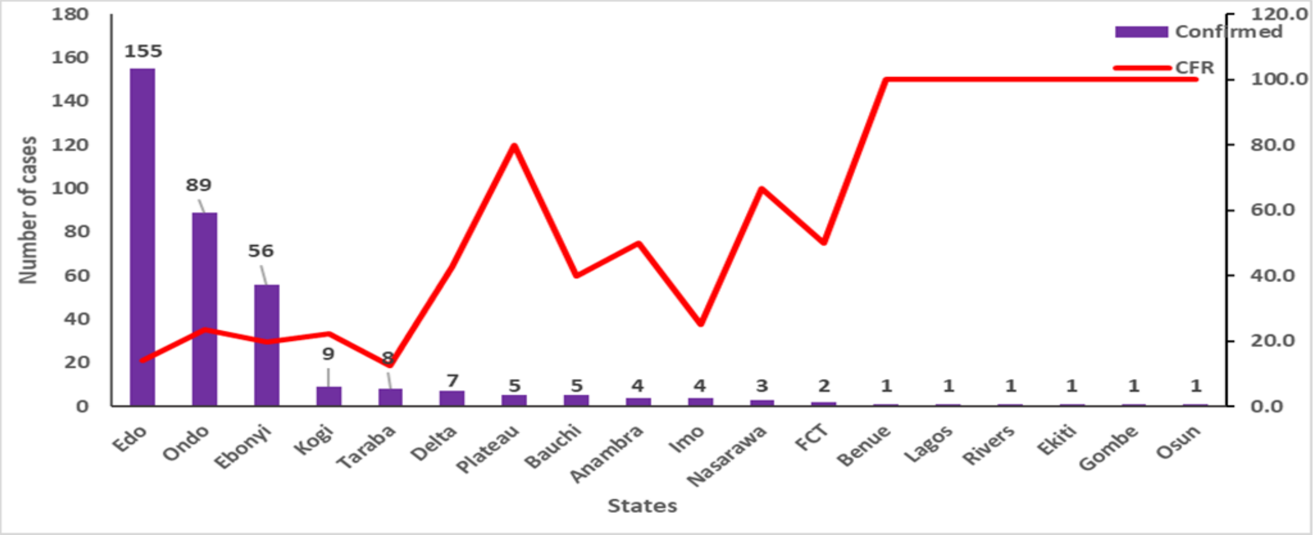

Figure 4. Confirmed Lassa fever cases in Nigeria with state specific case fatality rates (CFR) as at 4th March 2018

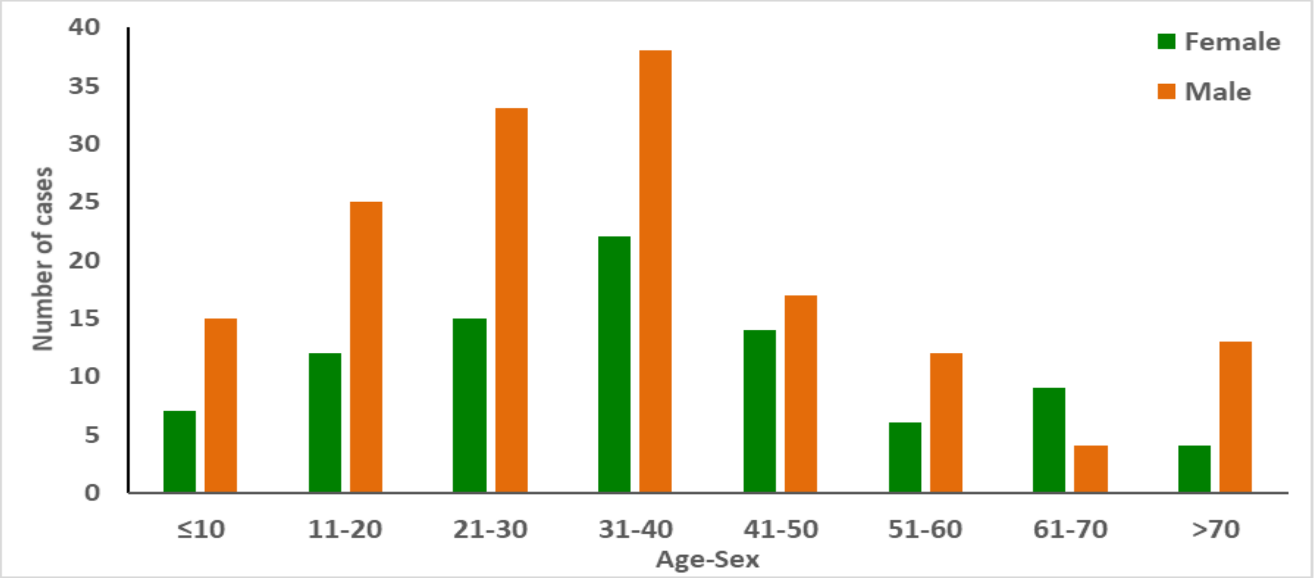

Figure 5. Age –Sex distribution of Confirmed Lassa fever cases in Nigeria as at 4th March 2018

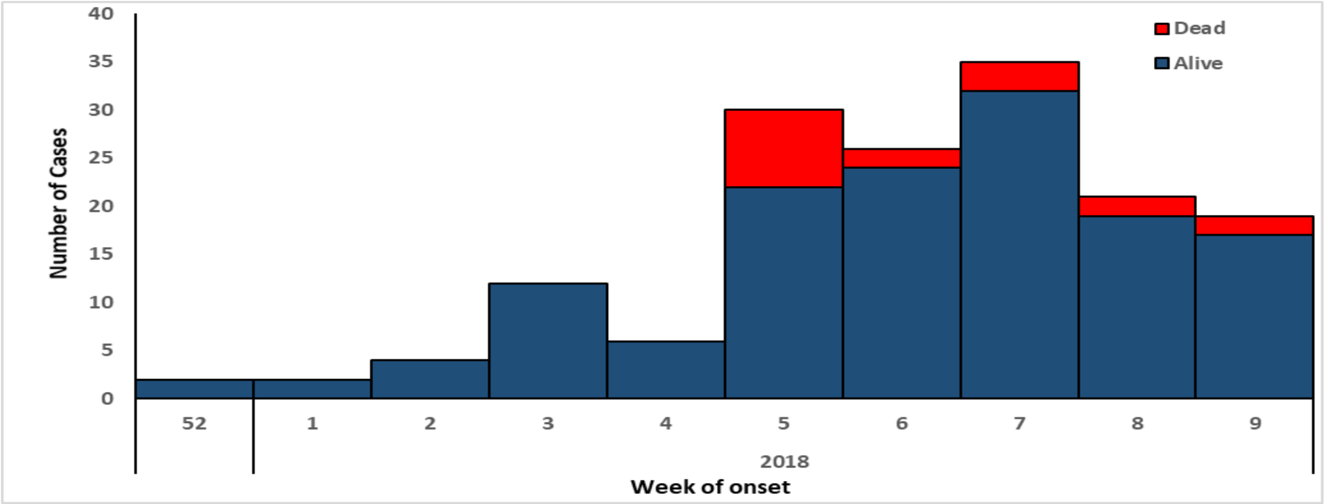

Figure 6: Epicurve of confirmed cases Lassa fever in Edo State week 52- 2017 to week 9- 2018

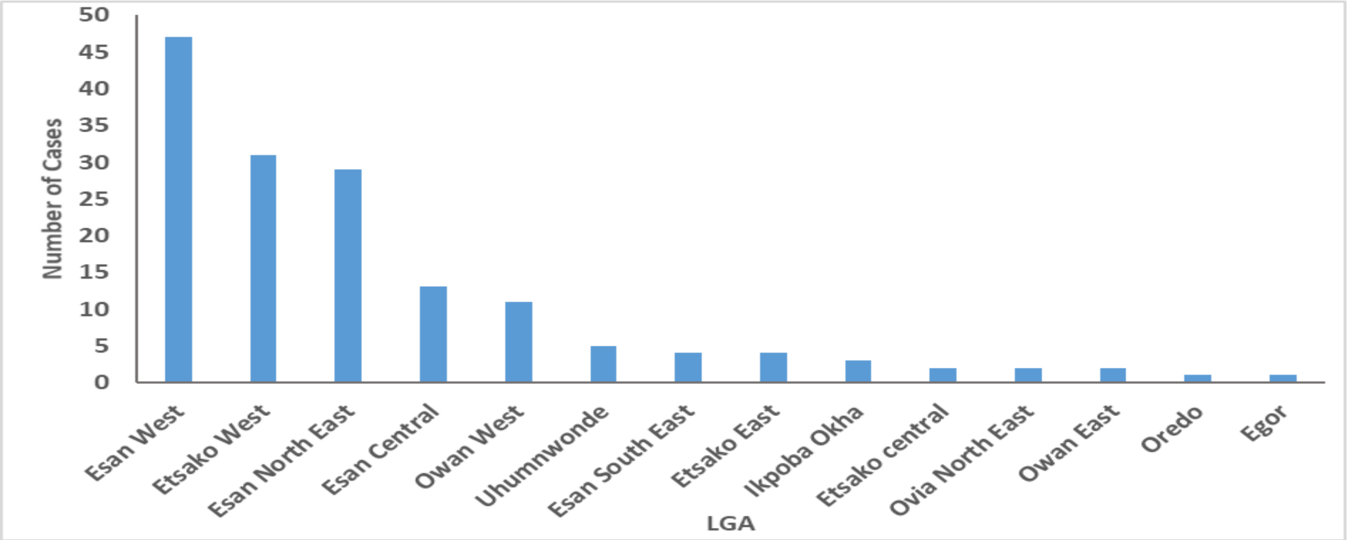

Figure 7: Distribution of Lassa fever confirmed cases in Edo State by LGA as at 4th March 2018

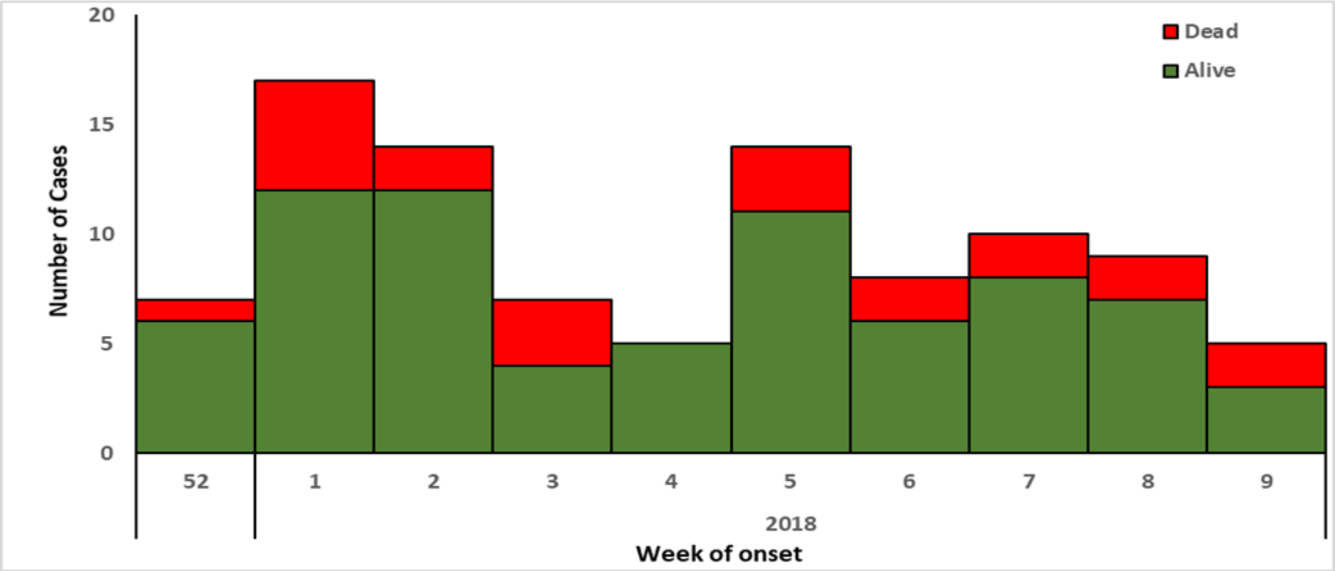

Figure 8: Epicurve of Lassa fever confirmed cases in Ondo State week 51 2017 to week 9 as at 4th March 2018

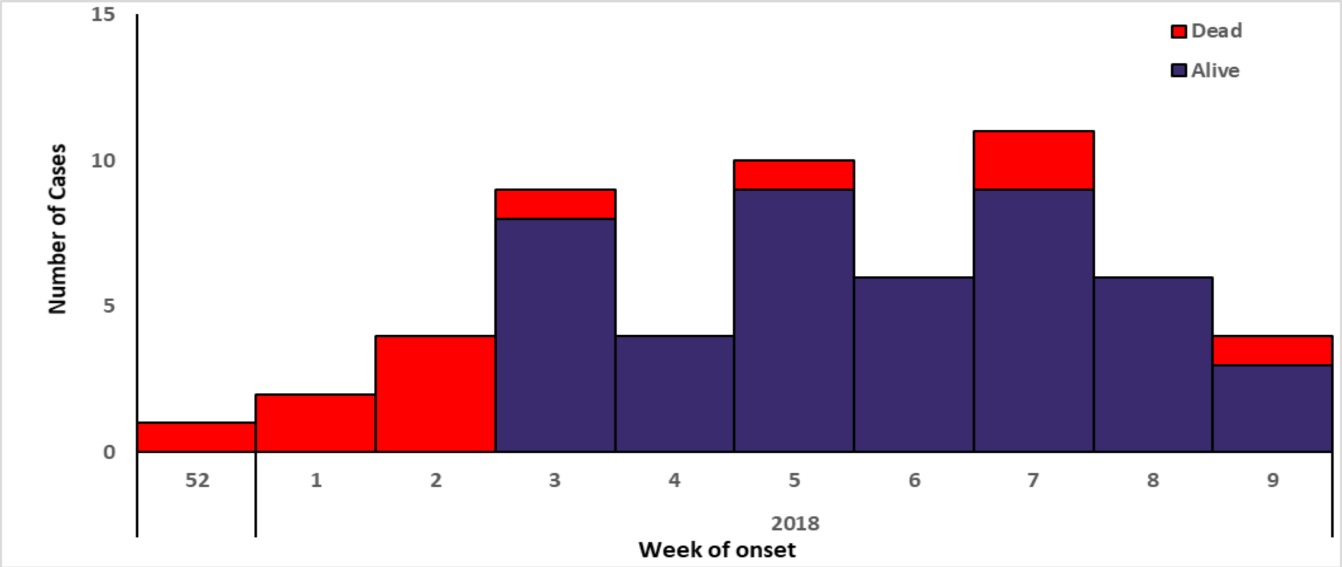

Figure 9: Epicurve of Lassa fever confirmed cases in Ebonyi State week 52 2017 to week 8 as at 4th March 2018

---

[i] *Suspected case describes any individual presenting with one or more of the following: malaise, fever, headache, sore throat, cough, nausea, vomiting, diarrhoea, myalgia, chest pain, hearing loss and either a. History of contact with excreta or urine of rodents b. History of contact with a probable or confirmed Lassa fever case within a period of 21 days of onset of symptoms OR Any person with inexplicable bleeding/hemorrhagia.*

[ii] *Any suspected case with laboratory confirmation (positive IgM antibody, PCR or virus isolation)*

[iii] *Any suspected case (see definition above) who died without collection of specimen for laboratory testing*

[iv] *"Active" means where there has been at least one confirmed case, and contacts within 21 days post exposure*

TITLE:	2018 LASSA FEVER OUTBREAK IN NIGERIA
SERIAL NUMBER:	10
EPI-WEEK:	10
DATE:	11 March 2018

HIGHLIGHTS

- **In the reporting Week 10 (March 5-11, 2018) twelve new confirmed[il] cases** were recorded from seven States Edo (3), Ondo (2), Bauchi (2), Ebonyi (1), Plateau (1), Taraba (1) Ekiti (1) and Kaduna(1) with **four new deaths,** three in confirmed cases from Plateau (1), Edo (1), and Kaduna (1) and one probable death in Ondo
- From 1st January to 11th March 2018, a total of 1386 **suspected[i] cases. Of these, 365 are confirmed positive, 9 are probable, 988 are negative (not a case) and 24 are awaiting laboratory results (pending**)
- Since the onset of the 2018 outbreak, there have been 114 deaths: 81 in positive-confirmed cases, 9 in probable cases and 24 in negative cases. Case Fatality Rate in confirmed and probable cases is 24% -*Table 1*
- 19 states have recorded at least one confirmed case across 55 Local Government Areas (Edo, Ondo, Bauchi, Nasarawa, Ebonyi, Anambra, Benue, Kogi, Imo, Plateau, Lagos, Taraba, Delta, Osun, Rivers, FCT, Gombe, Ekiti and Kaduna). Kaduna state reported one new confirmed case for the first time in this outbreak. Seven states have exited the active phase of the outbreak; 12 States remain active[iv] - *Table 1/ Figure 1*
- In the reporting week 10, no healthcare worker was affected. **Sixteen health care workers have been affected since the onset of the outbreak in six states** –Ebonyi (9), Nasarawa (1), Kogi (1), Benue (1), Ondo (1) and Edo (3) with four deaths in Ebonyi (3) and Kogi (1)
- 84% of all confirmed cases are from Edo (43%) Ondo (25%) and Ebonyi (16%) states
- 36 cases are currently under treatment in treatment centres across seven states
- National RRT team (NCDC staff and NFELTP residents) batch C has been deployed to replace batch B in Ebonyi, Ondo, Edo and new team deployed to Bauchi State
- A total of 3323 contacts have been identified from 19 active states. Of these 1150 are currently being followed up, 2182 have completed 21 days follow up and **21** of the 54 symptomatic contacts have tested positive from 3 states (Edo-11, Ondo-7 and Ebonyi-3)
- WHO and NCDC has scaled up response at National and State levels
- NCDC deployed teams to four Benin Republic border states (Kebbi, Kwara, Niger and Oyo) for enhanced surveillance activities
- National Lassa fever multi-partner multi-agency Emergency Operations Centre (EOC) continues to coordinate the response activities at all levels

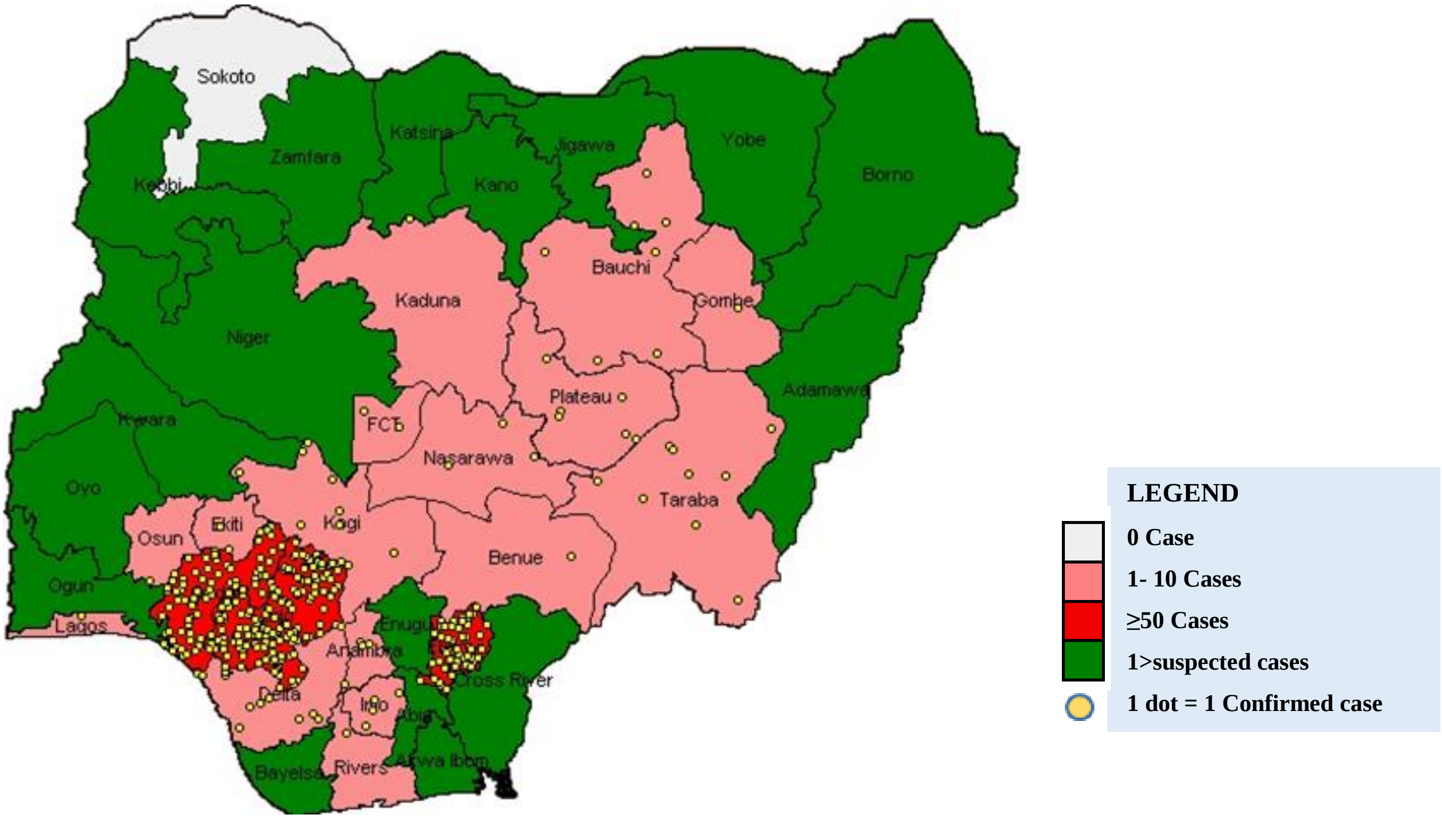

Figure 1. Distribution of Confirmed Lassa Fever cases in Nigeria as at 11th March, 2018

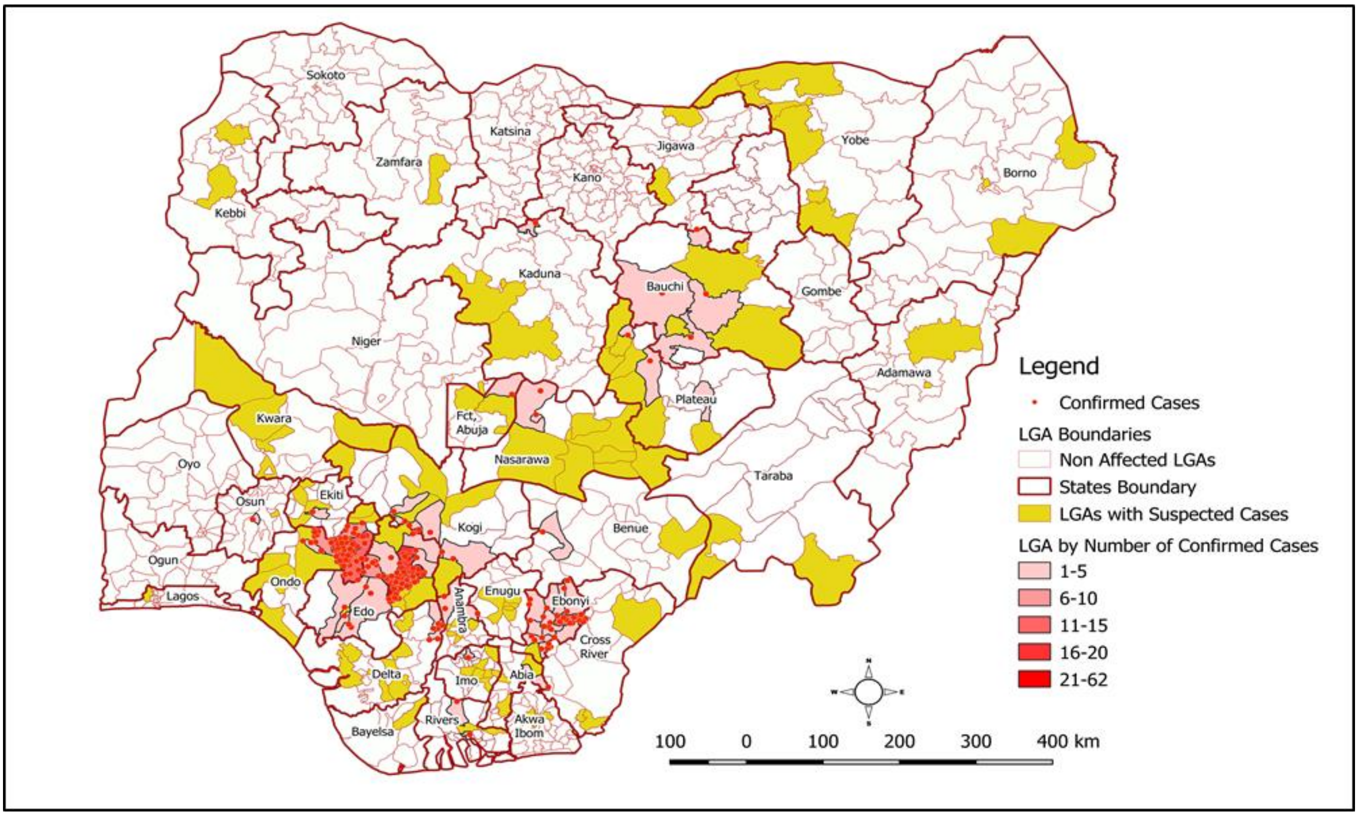

Figure 2. Distribution of Suspected and Confirmed Lassa Fever cases in Nigeria by LGA

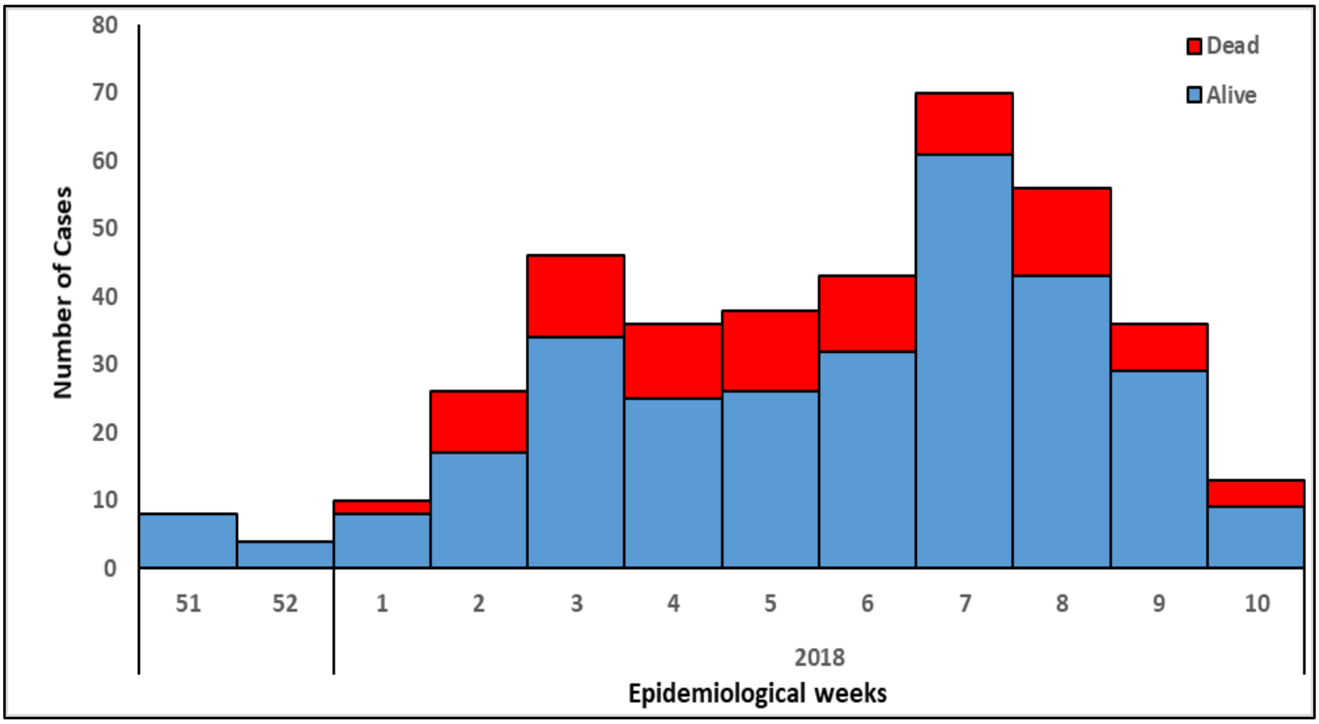

Figure 3. Confirmed (365) and Probable (9) Cases of Lassa fever by epidemiological week in Nigeria week 50, 2017 to week 10, 2018

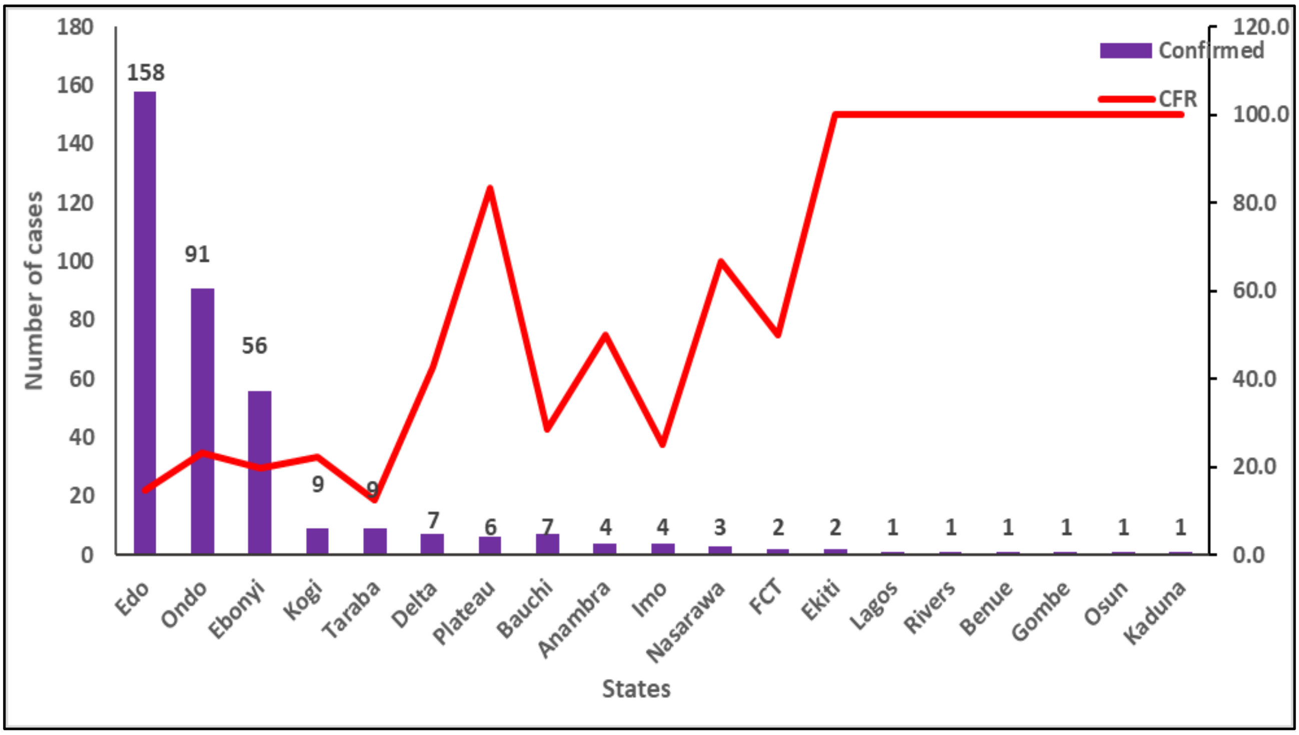

Figure 4. Confirmed Lassa fever cases in Nigeria with state specific Case Fatality Rates (CFR) as at 11th March 2018

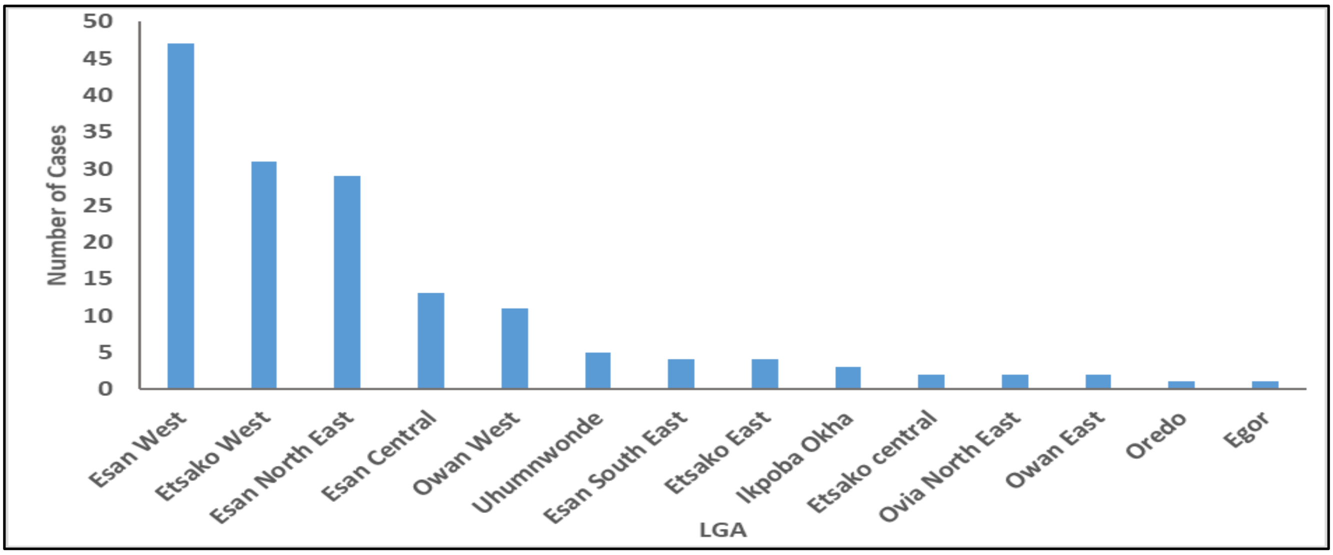

Figure 5: Epicurve of confirmed cases Lassa fever in Edo State week 52, 2017 to week 9, 2018

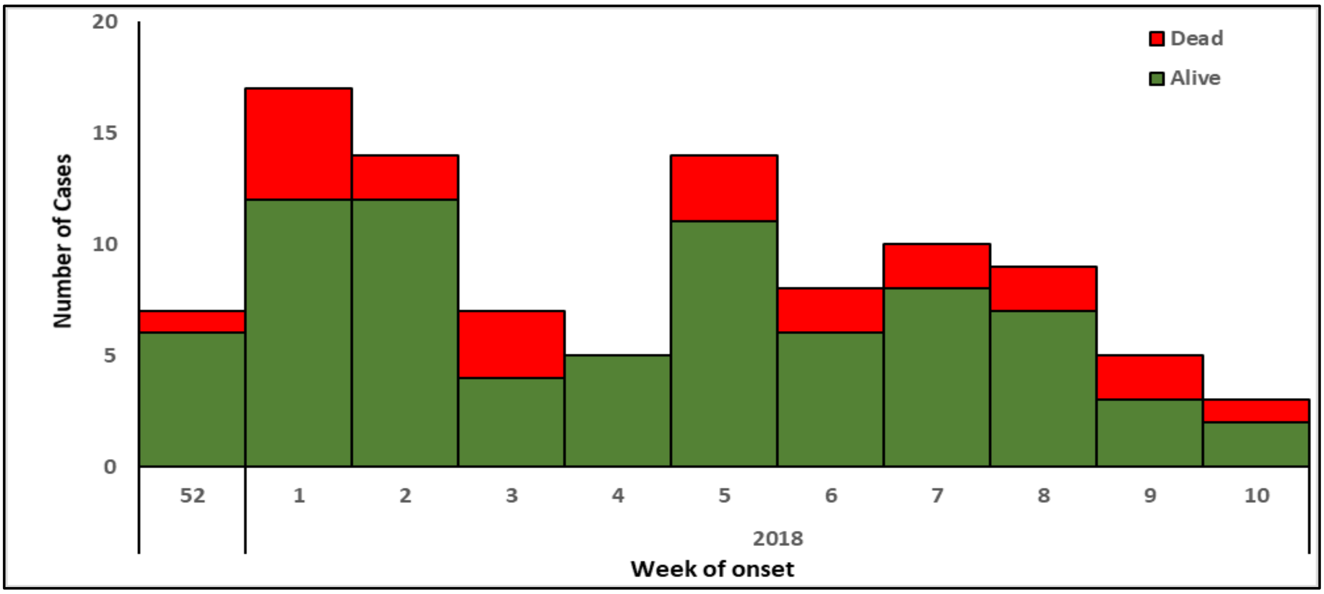

Figure 6: Distribution of Lassa fever confirmed cases in Edo State by LGA as at 11th March 2018

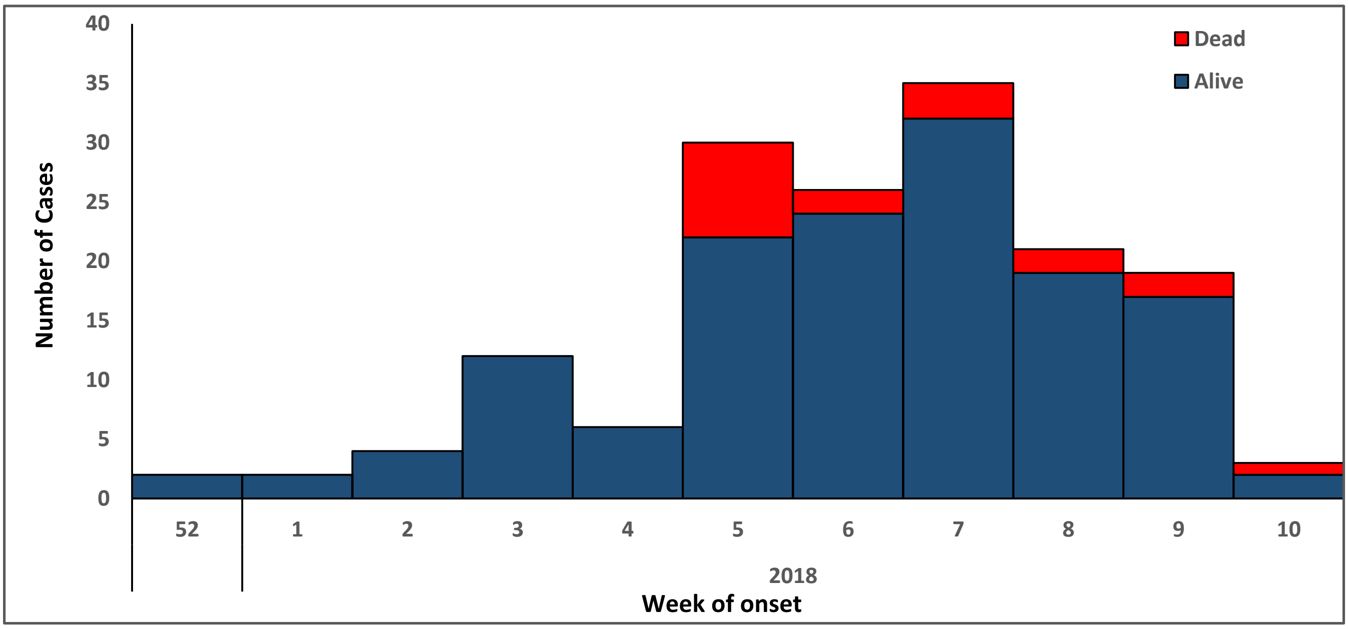

Figure 7: Epicurve of Lassa fever confirmed cases in Ondo State week 52, 2017 to week 10, 2018

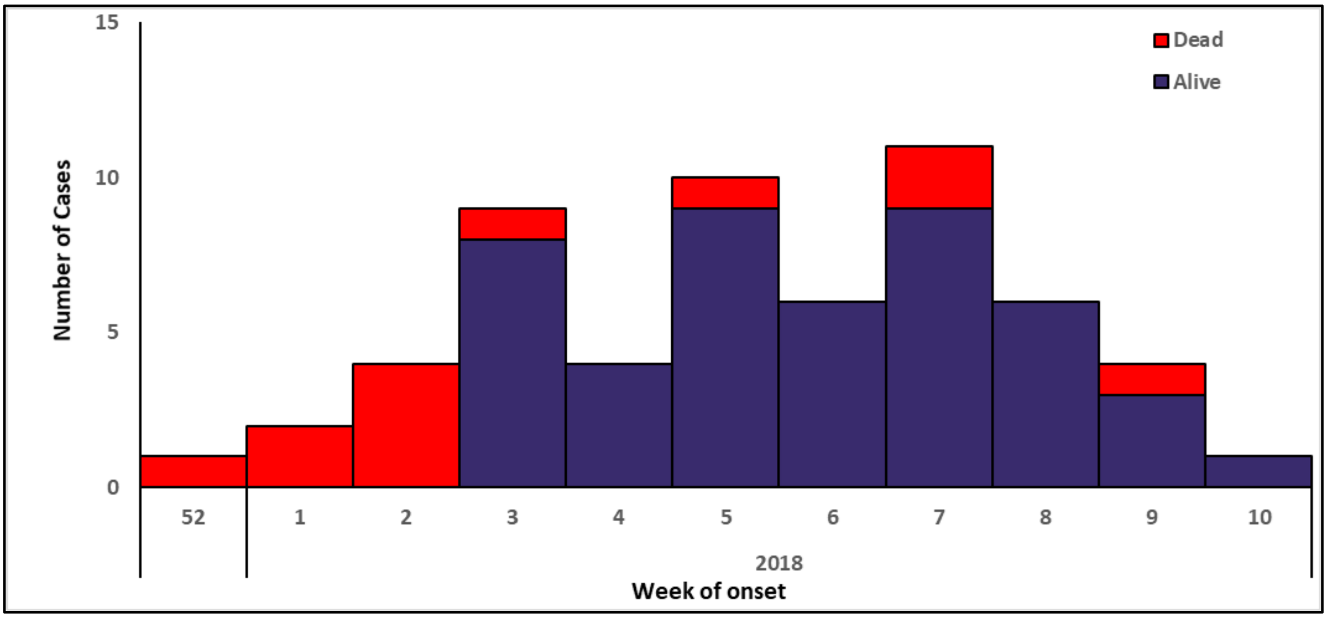

Figure 8: Epicurve of Lassa fever confirmed cases in Ebonyi State week 52, 2017 to week 10, 2018

---

[i]*Suspected case describes any individual presenting with one or more of the following: malaise, fever, headache, sore throat, cough, nausea, vomiting, diarrhoea, myalgia, chest pain, hearing loss and either a. History of contact with excreta or urine of rodents b. History of contact with a probable or confirmed Lassa fever case within a period of 21 days of onset of symptoms OR Any person with inexplicable bleeding/hemorrhagia.*

[ii]*Any suspected case with laboratory confirmation (positive IgM antibody, PCR or virus isolation)*

[iii]*Any suspected case (see definition above) who died without collection of specimen for laboratory testing*

[iv] *"Active" means where there has been at least one confirmed case, and contacts within 21 days post exposure*

SITUATION REPORT
Nigeria Centre For Disease Control (NCDC)

NIGERIA CENTRE FOR DISEASE CONTROL

www.ncdc.gov.ng PLOT 800 EBITU UKIWE STREET, JABI ABUJA, NIGERIA. **TOLL FREE CALL:** 0800-970000-10. **E:** info@ncdc.gov.ng @NCDCgov

**TITLE:**	**2018 LASSA FEVER OUTBREAK IN NIGERIA**
**SERIAL NUMBER:**	**11**
**EPI-WEEK:**	**11**
**DATE:**	**18 March 2018**

HIGHLIGHTS

- **In the reporting Week 11 (March 12-18, 2018) nine new confirmed[ii] cases** were recorded from four States Edo (2), Ondo (2), Bauchi (1), and Taraba (4) with **three new deaths** in confirmed cases from Taraba (1), Edo (1) and Ebonyi (1)
- From 1st January to 18th March 2018, a total of 1495 **suspected[i] cases. Of these, 376* confirmed positive, 9 are probable,** 1084 are negative (not a case) and 26 are awaiting laboratory results (pending)
- Since the onset of the 2018 outbreak, there have been 119 deaths: 86 in positive-confirmed cases, 9 in probable cases and 24 in negative cases. Case Fatality Rate in confirmed and probable cases is 24.7%
- 19 states have recorded at least one confirmed case across 56 Local Government Areas (Edo, Ondo, Bauchi, Nasarawa, Ebonyi, Anambra, Benue, Kogi, Imo, Plateau, Lagos, Taraba, Delta, Osun, Rivers, FCT, Gombe, Ekiti and Kaduna). Nine states have exited the active phase of the outbreak, 10 States remain active[iv] - *Figure 1*
- In the reporting week 11, no new healthcare worker was affected. **Seventeen+ health care workers have been affected since the onset of the outbreak in six states** –Ebonyi (9), Nasarawa (1), Kogi (2), Benue (1), Ondo (1) and Edo (3) with four deaths in Ebonyi (3) and Kogi (1)
- 83% of all confirmed cases are from Edo (43%) Ondo (24%) and Ebonyi (16%) states
- 38 cases are currently under treatment in treatment centres across six states (Edo (10), Ondo (8), Ebonyi (4), Bauchi (10), Taraba (5) and Plateau (1)
- A total of 3675 contacts have been identified from 19 states. Of these 805 are currently being followed up, 2863 have completed 21 days follow up while 7 were lost follow up. Twenty-three (**23)** of the 59 symptomatic contacts have tested positive from 3 states (Edo-12, Ondo-7, Ebonyi-3 and Kogi -1)
- WHO and NCDC has scaled up response at National and State levels
- National RRT team (NCDC staff and NFELTP residents) batch C continues response support in Ebonyi, Ondo, Edo and Bauchi State
- NCDC deployed teams to four Benin Republic border states (Kebbi, Kwara, Niger and Oyo) for enhanced surveillance activities
- National Lassa fever multi-partner multi-agency Emergency Operations Centre (EOC) continues to coordinate the response activities at all levels

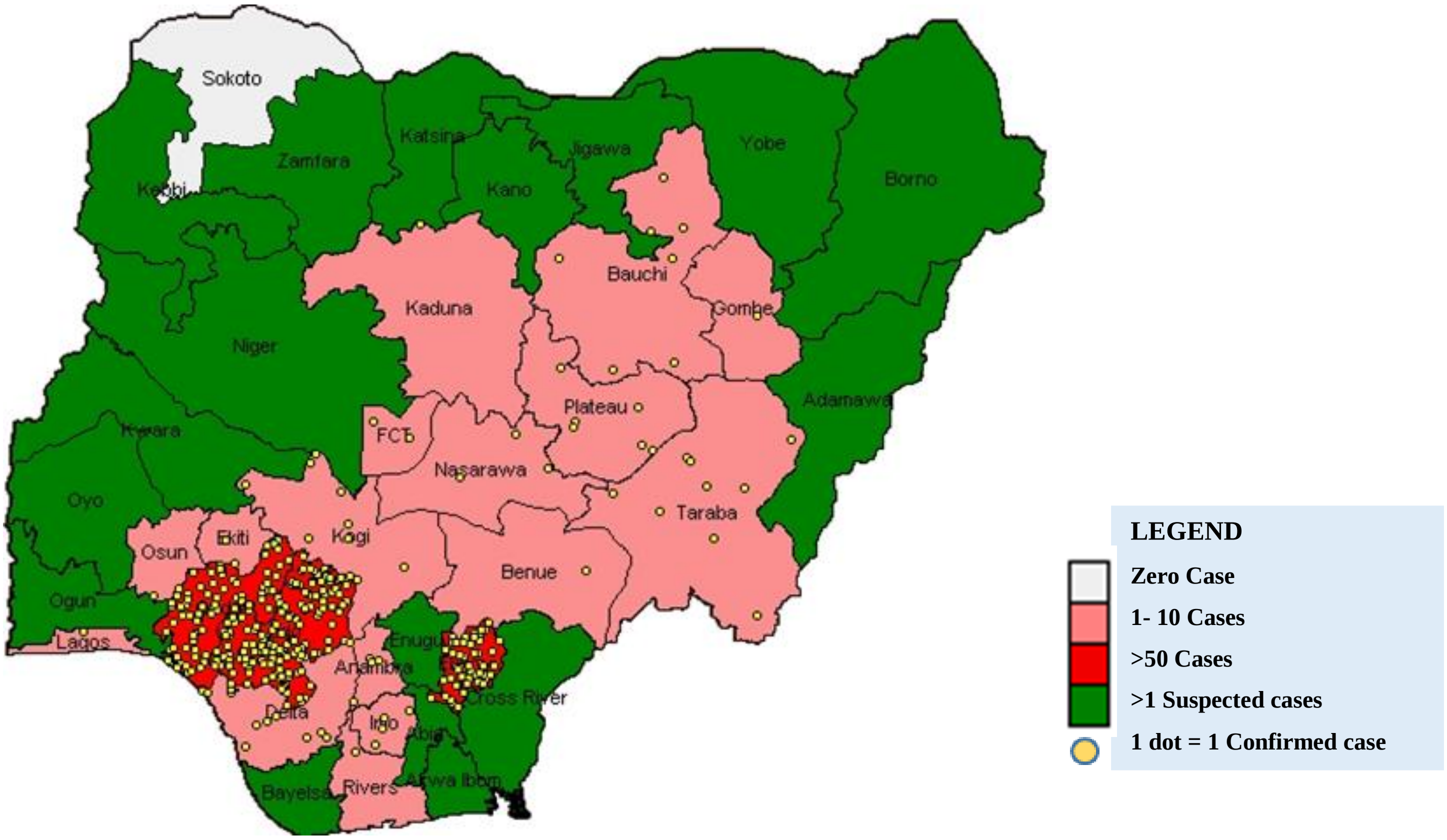

Figure 1. Distribution of Confirmed Lassa Fever cases in Nigeria as at 18th March, 2018

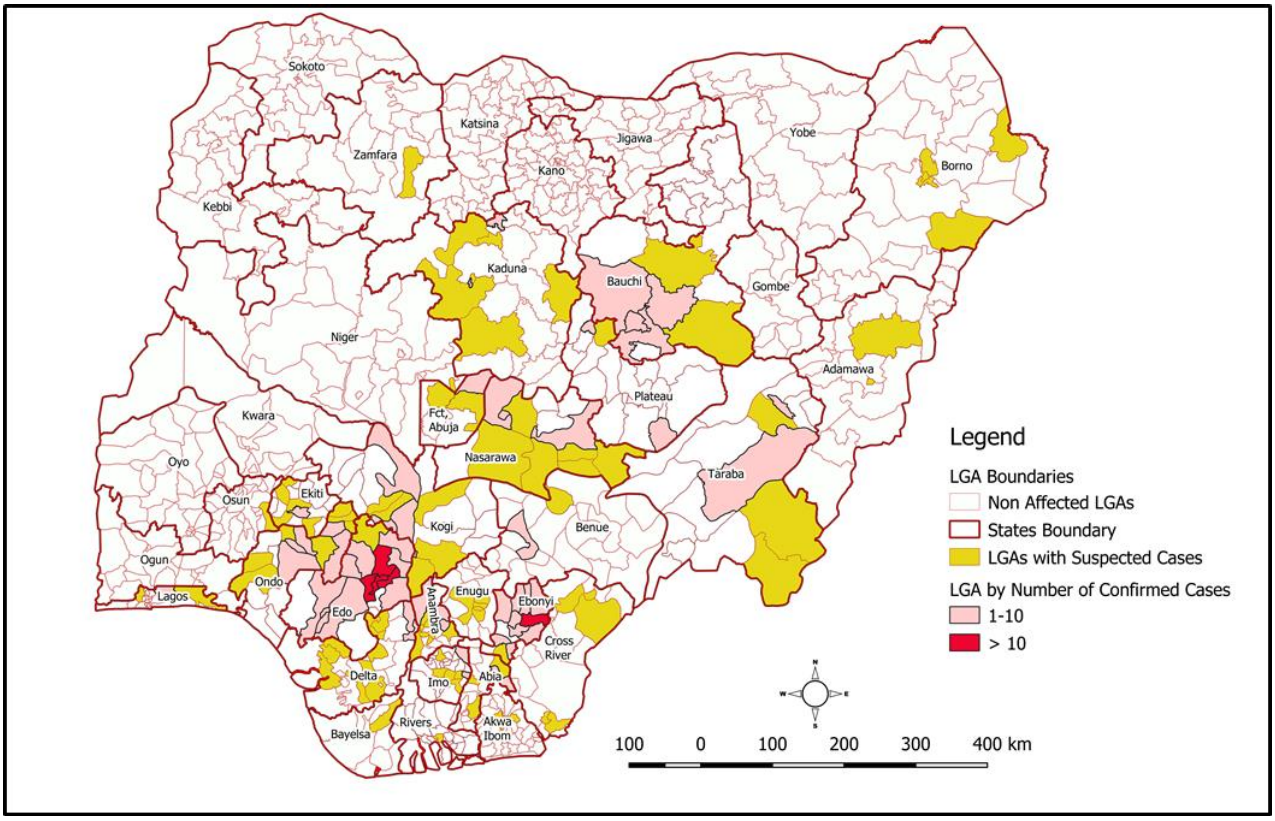

Figure 2. Distribution of Suspected and Confirmed Lassa Fever cases in Nigeria by LGA

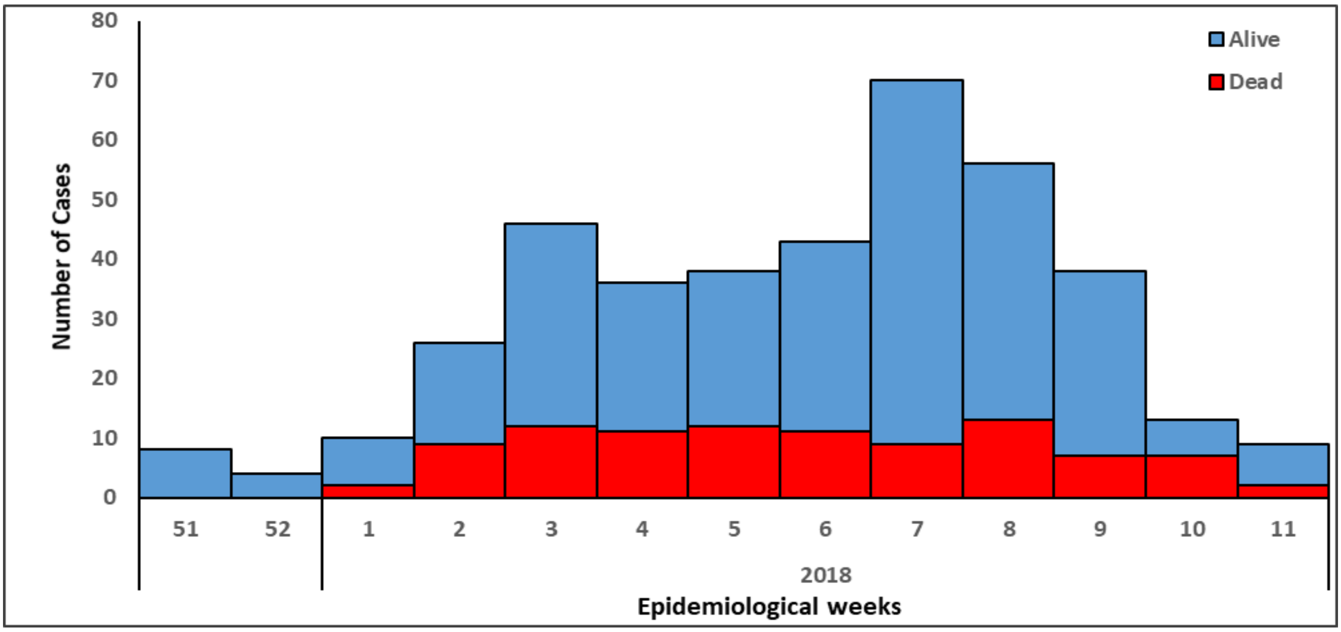

Figure 3. Confirmed (376) and Probable (9) Cases of Lassa fever by epidemiological week in Nigeria week 51, 2017 to week 11, 2018

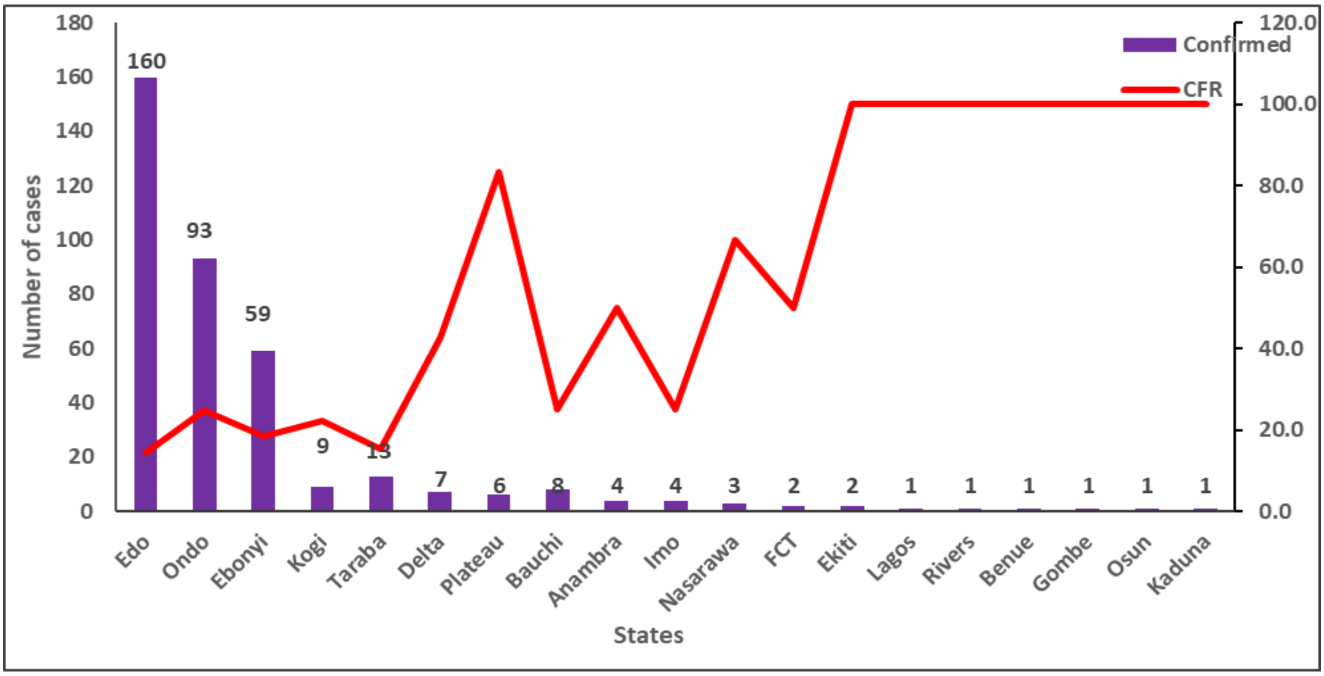

Figure 4. Confirmed Lassa fever cases in Nigeria with state specific Case Fatality Rates (CFR) as at 18th March 2018

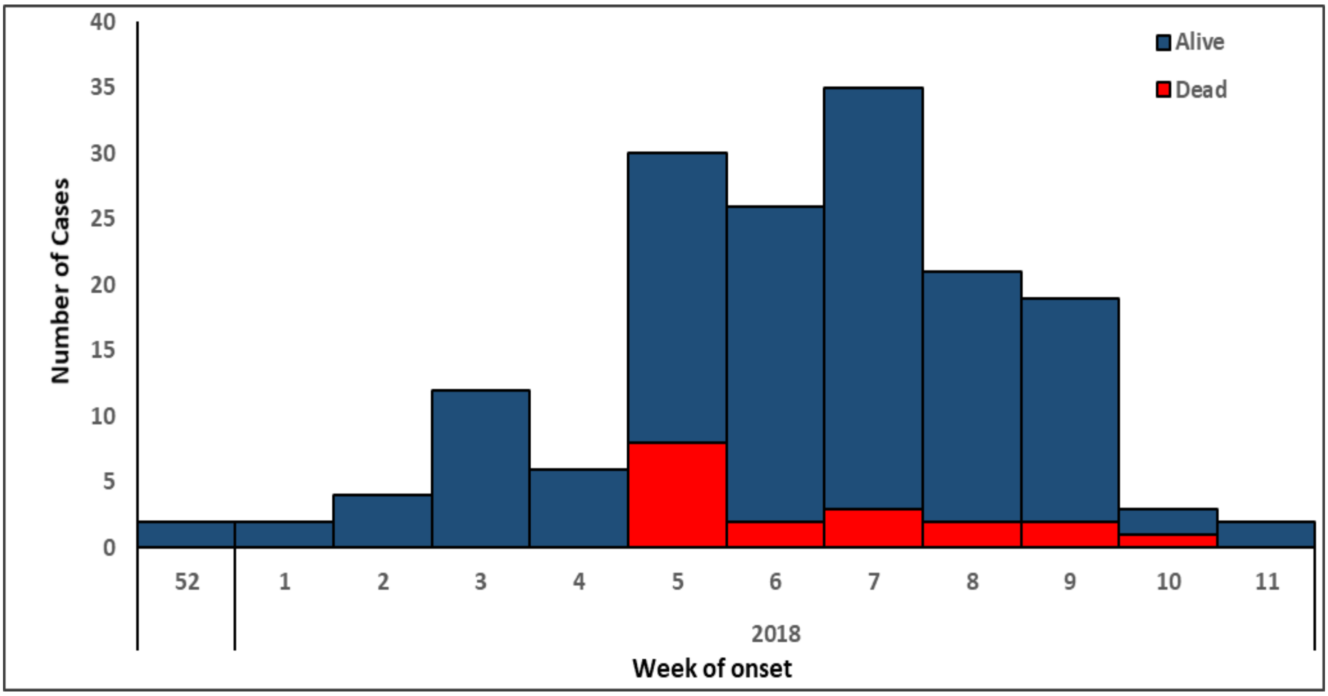

Figure 5: Epicurve of confirmed cases Lassa fever in Edo State week 52, 2017 to week 11, 2018

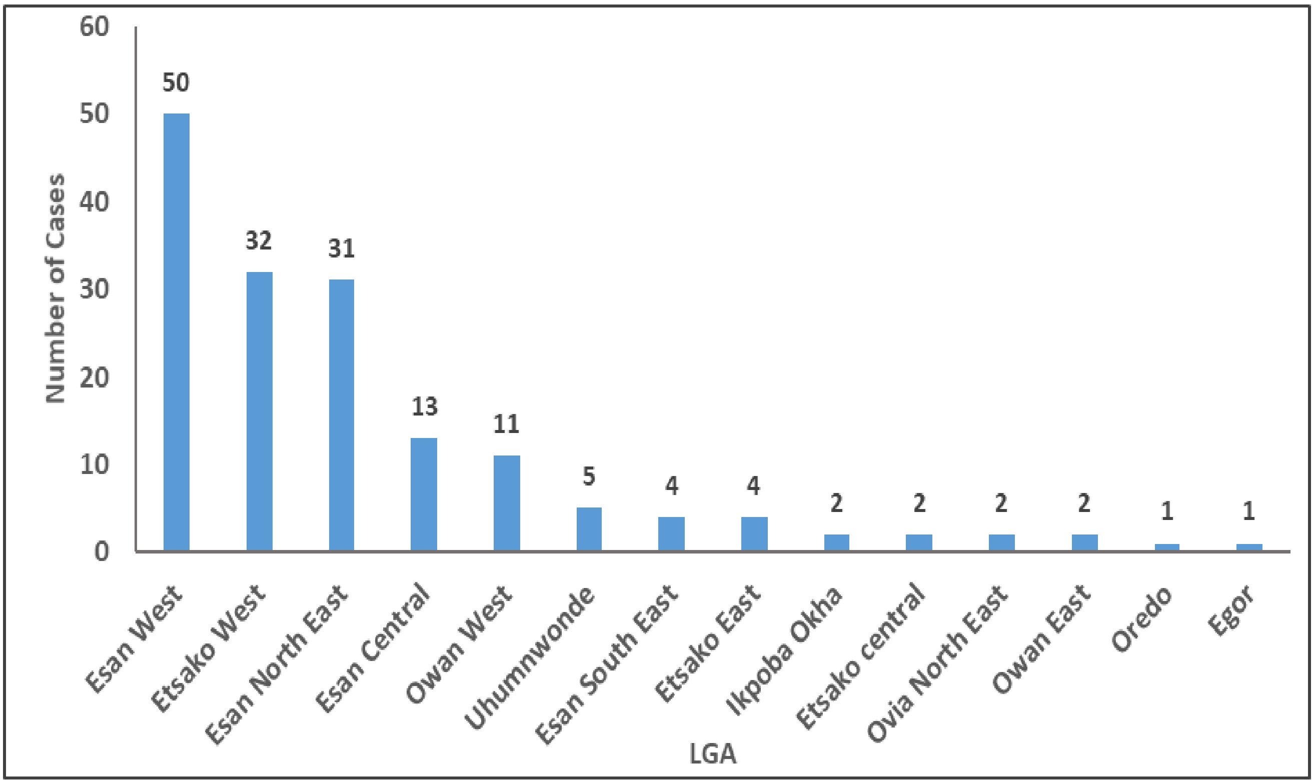

Figure 6: Distribution of Lassa fever confirmed cases in Edo State by LGA as at 18th March 2018

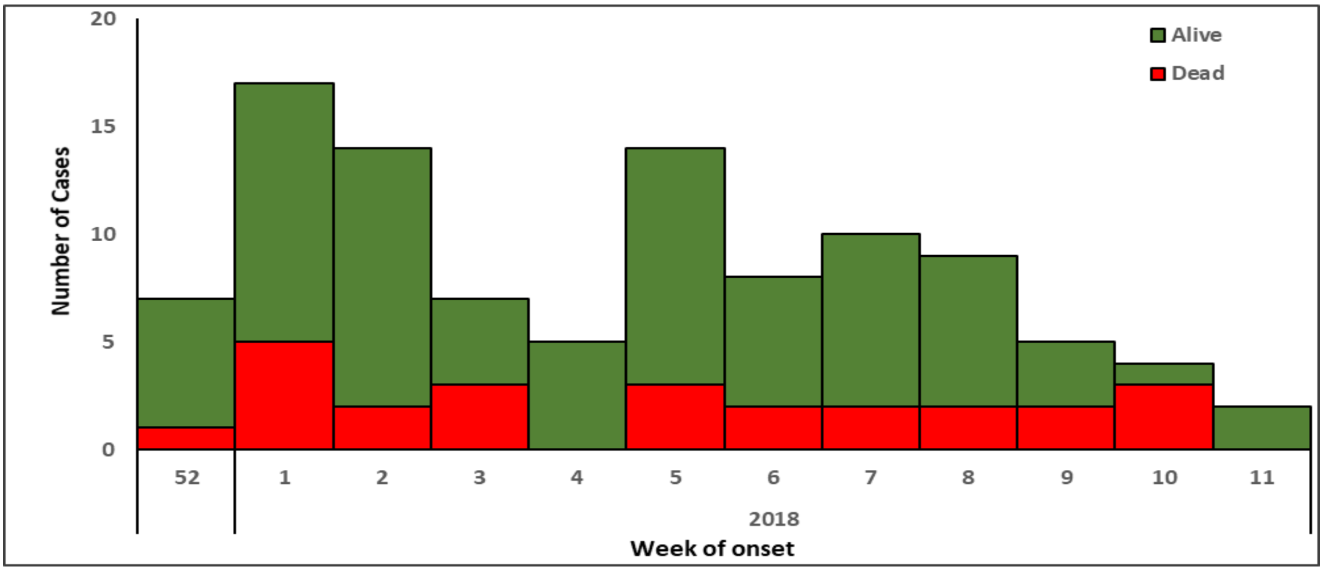

Figure 7: Epicurve of Lassa fever confirmed cases in Ondo State week 52, 2017 to week 11, 2018

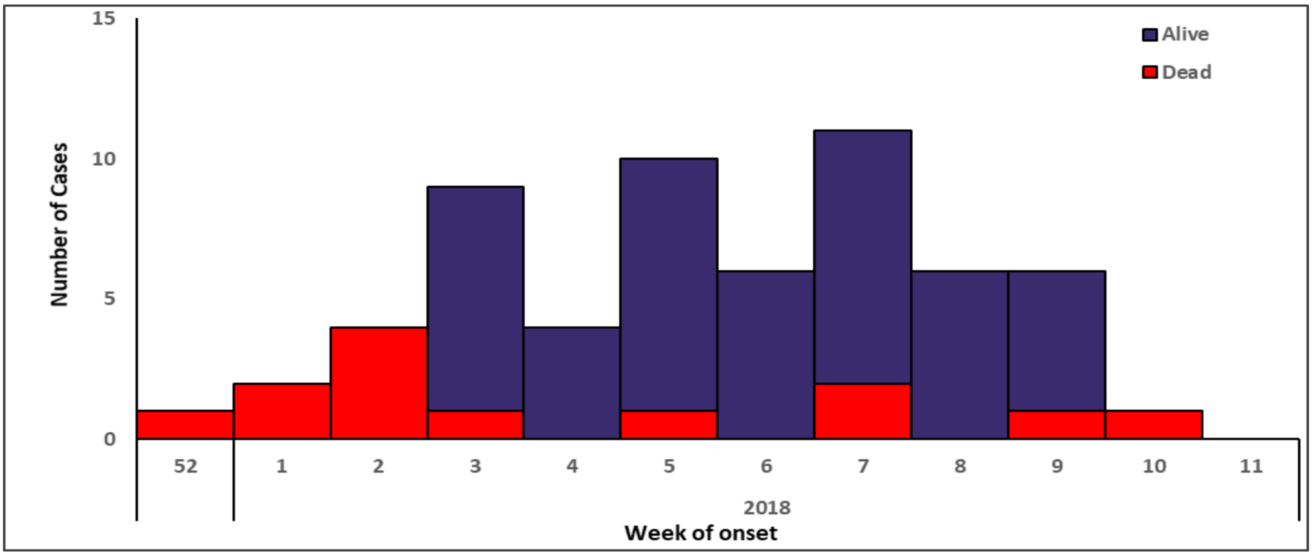

Figure 8: Epicurve of Lassa fever confirmed cases in Ebonyi State week 52, 2017 to week 11, 2018

---

[i]*Suspected case describes any individual presenting with one or more of the following: malaise, fever, headache, sore throat, cough, nausea, vomiting, diarrhoea, myalgia, chest pain, hearing loss and either a. History of contact with excreta or urine of rodents b. History of contact with a probable or confirmed Lassa fever case within a period of 21 days of onset of symptoms OR Any person with inexplicable bleeding/hemorrhagia.*

[ii]*Any suspected case with laboratory confirmation (positive IgM antibody, PCR or virus isolation)*

[iii]*Any suspected case (see definition above) who died without collection of specimen for laboratory testing*

[iv] *"Active" means where there has been at least one confirmed case, and contacts within 21 days post exposure*

**Ebonyi state data validation revealed 2 additional retrospective cases in week 9*

+One HCW (Doctor) was infected in week 09

**TITLE:**	**2018 LASSA FEVER OUTBREAK IN NIGERIA**
**SERIAL NUMBER:**	**12**
**EPI-WEEK:**	**12**
**DATE:**	**25 March 2018**

HIGHLIGHTS

- **In the reporting Week 12 (March 19-25, 2018) eighteen new confirmed[ii] cases** were recorded from ten States - Edo (5), Ondo (2), Bauchi (1), Ebonyi (2), Taraba (3),Plateau (1), Kogi (1), Osun (1) FCT (1)and Gombe(1) with **six new deaths** in confirmed cases from Edo (1), Taraba (2), Ebonyi (1),Plateau (1) and Gombe (1)
- From 1st January to 25th March 2018, a total of 1613 **suspected[i] cases. Of these, 394 confirmed positive, 9 are probable,** 1198 are negative (not a case) and 12 are awaiting laboratory results (pending)
- Since the onset of the 2018 outbreak, there have been 134 deaths: 95 in positive-confirmed cases, 9 in probable cases and 30 in negative cases. Case Fatality Rate in confirmed cases is 24.1% -*Table 1*
- 19 states have recorded at least one confirmed case across 56 Local Government Areas (Edo, Ondo, Bauchi, Nasarawa, Ebonyi, Anambra, Benue, Kogi, Imo, Plateau, Lagos, Taraba, Delta, Osun, Rivers, FCT, Gombe, Ekiti and Kaduna). Seven states have exited the active phase of the outbreak while 12 States remain active[iv] - *Table 1/ Figure 1*
- In the reporting week 12, no new healthcare worker was affected. **Seventeen health care workers have been affected since the onset of the outbreak in six states** –Ebonyi (9), Nasarawa (1), Kogi (2), Benue (1), Ondo (1) and Edo (3) with four deaths in Ebonyi (3) and Kogi (1)
- 81% of all confirmed cases are from Edo (42%) Ondo (23%) and Ebonyi (16%) states
- 27 cases are currently under treatment in treatment centres across nine states -Edo (9), Ondo (5), Ebonyi (5), Bauchi (2), Taraba (3), Plateau (1), Osun (1) and Kogi (1)
- A total of 4005 contacts have been identified from 19 states. Of these 763(19.0%) are currently being followed up, 3235(80.8%) have completed 21 days follow up while 7(0.2%) were lost follow up. **23 (36%)** of the 64 symptomatic contacts have tested positive from 3 states (Edo-12, Ondo-7, Ebonyi-3 and Kogi -1)
- WHO and NCDC has scaled up response at National and State levels
- National RRT team (NCDC staff and NFELTP residents) batch C continues response support in Ebonyi, Ondo, Edo, Bauchi and Taraba State
- National Lassa fever multi-partner multi-agency Emergency Operations Centre (EOC) continues to coordinate the response activities at all levels

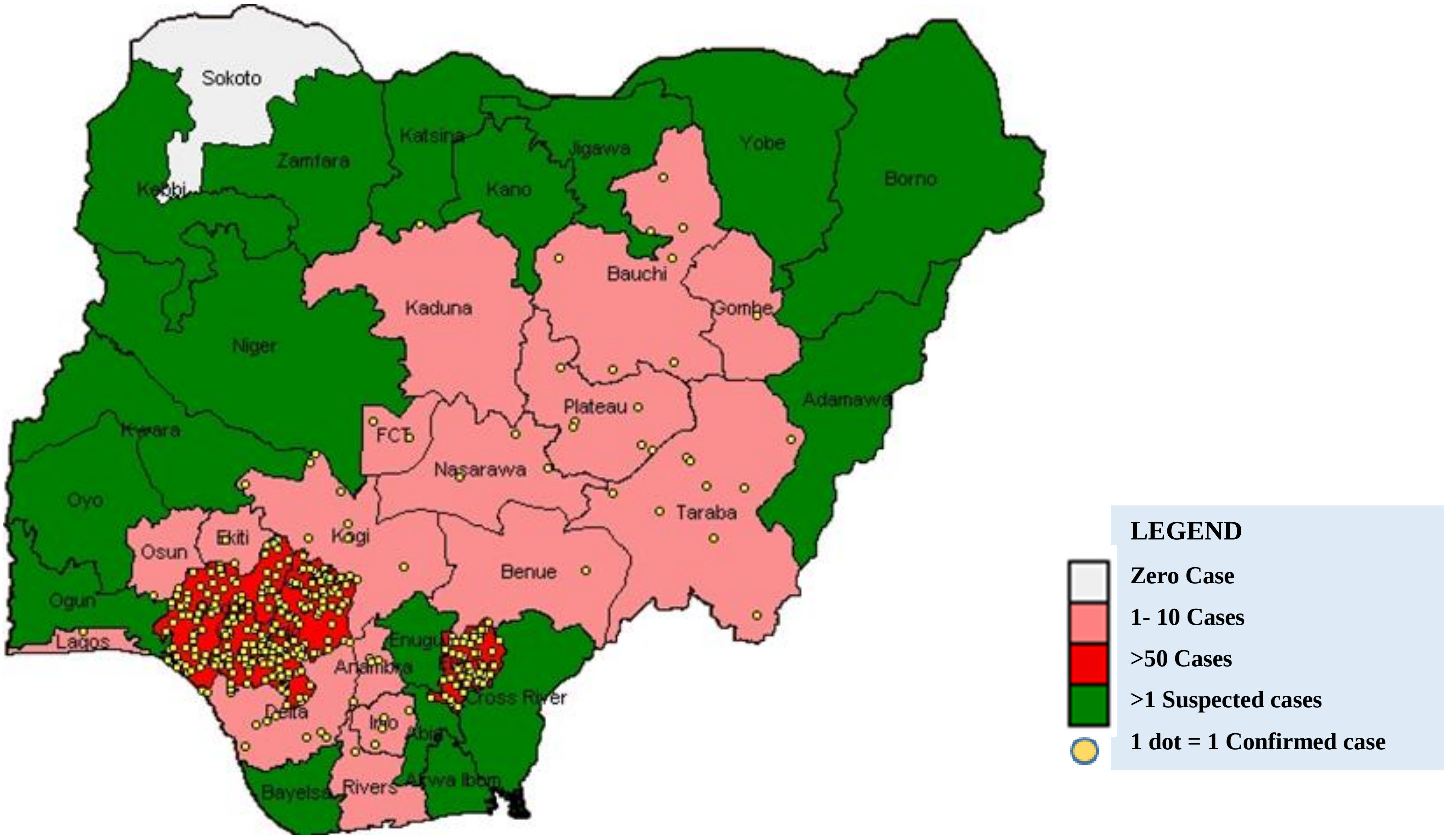

Figure 1. Distribution of Confirmed Lassa Fever cases in Nigeria as at 25th March, 2018

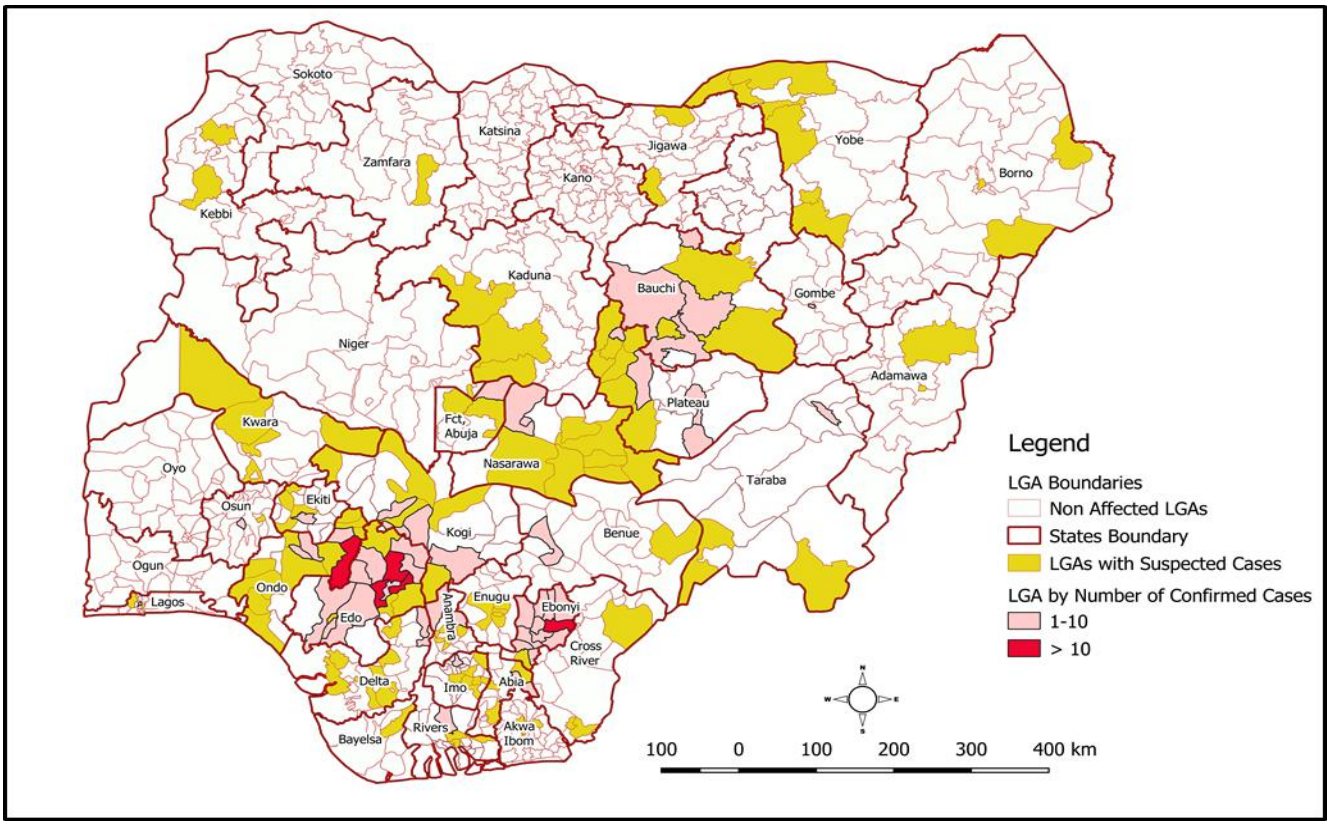

Figure 2. Distribution of Suspected and Confirmed Lassa Fever cases in Nigeria by LGA

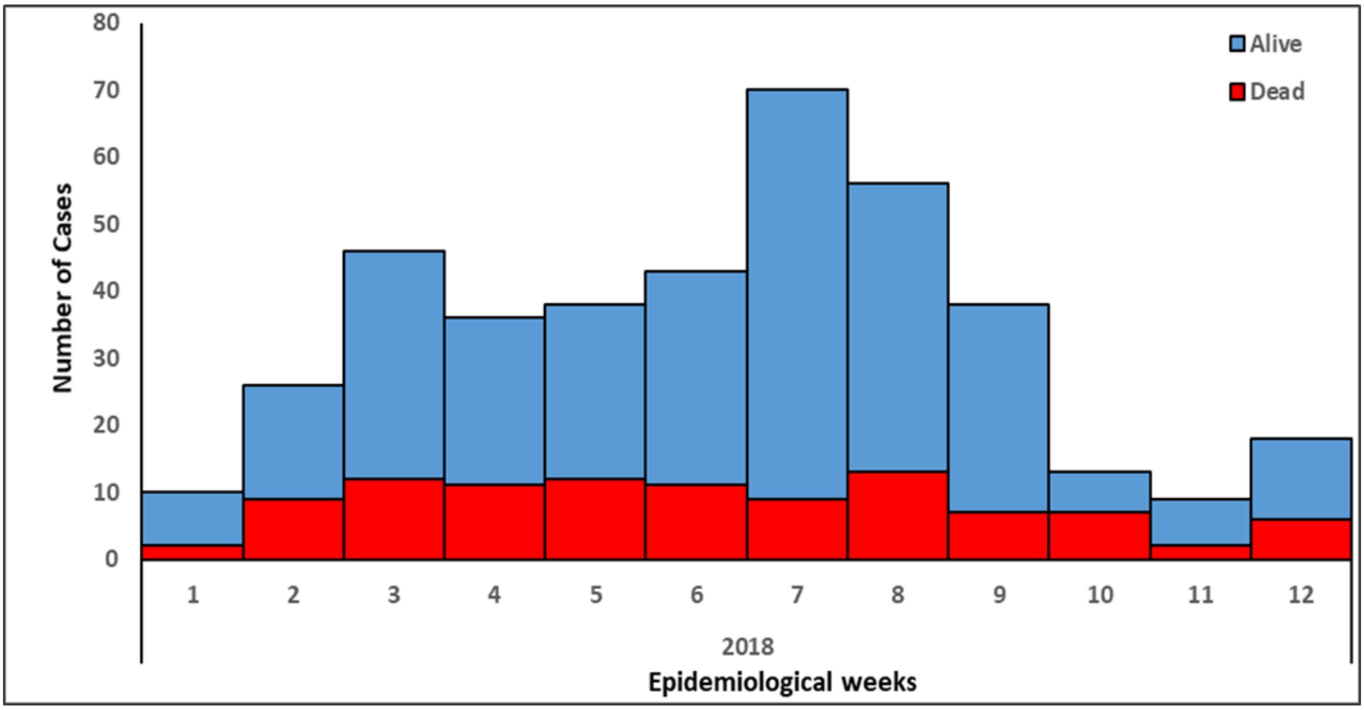

Figure 3. Confirmed (394) and Probable (9) Cases of Lassa fever by epidemiological week in Nigeria week 1-12, 2018

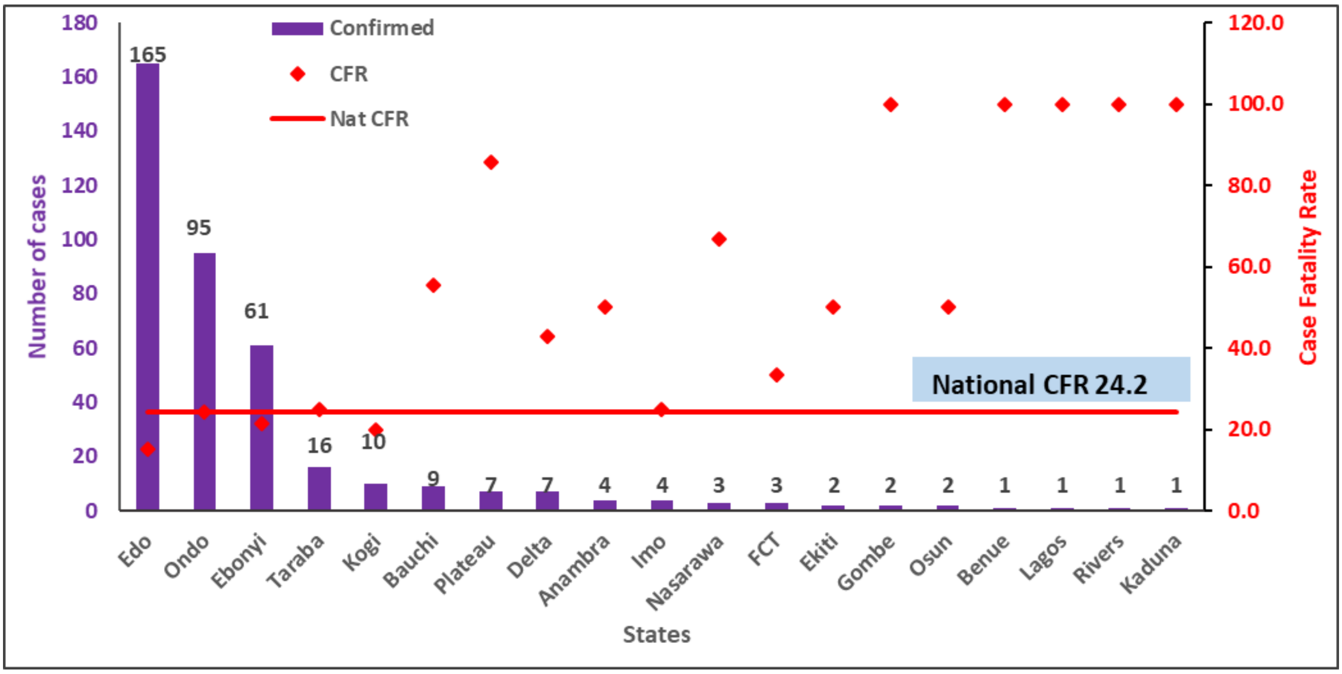

Figure 4. Confirmed Lassa fever cases in Nigeria with state specific Case Fatality Rates (CFR) as at 25th March 2018

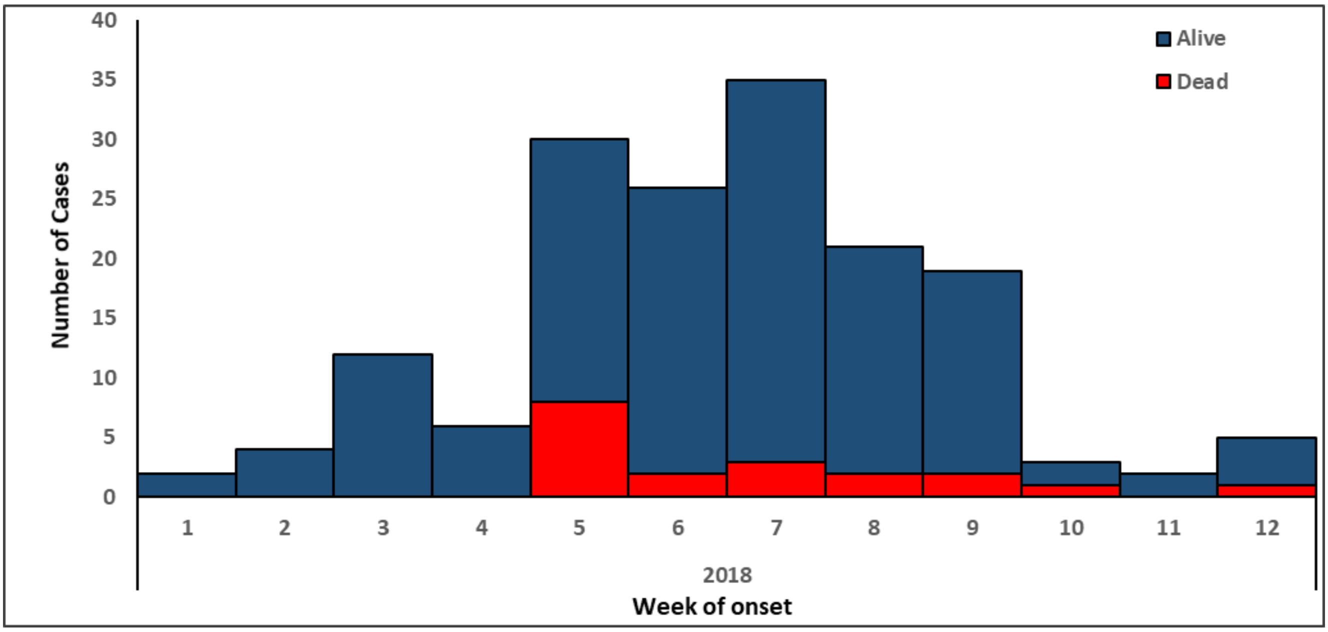

Figure 5: Epicurve of confirmed cases Lassa fever in Edo State week 1-12, 2018

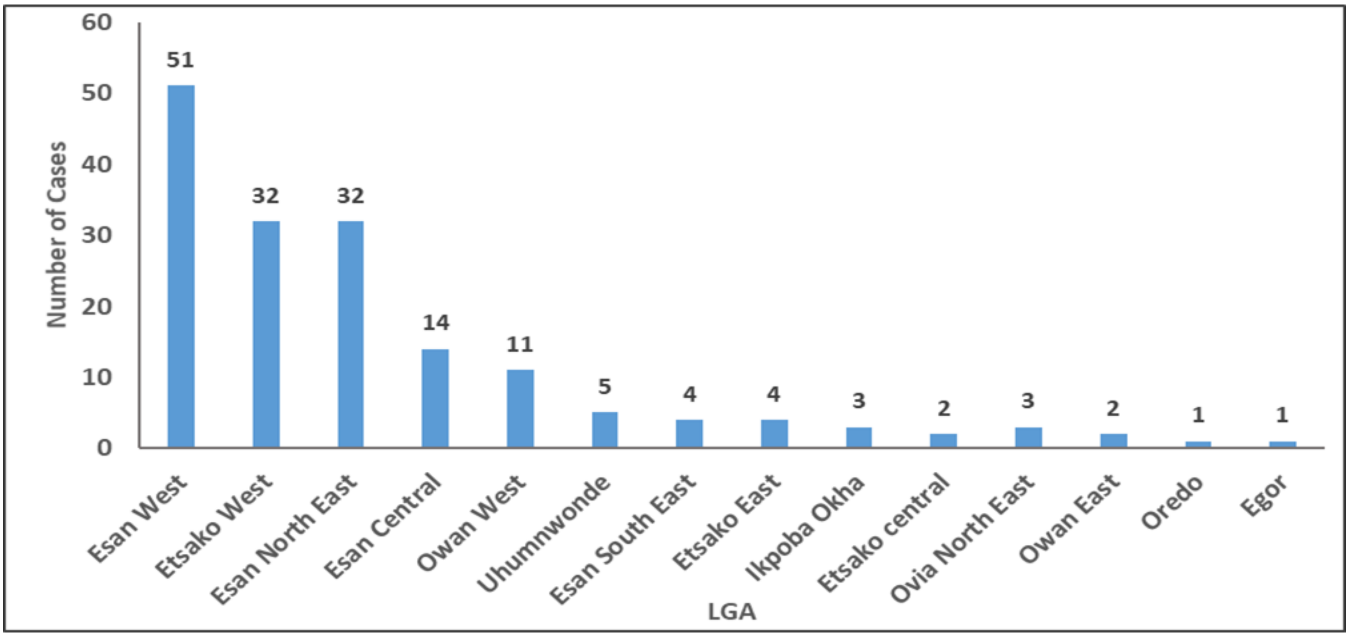

Figure 6: Distribution of Lassa fever confirmed cases in Edo State by LGA as at 25th March 2018

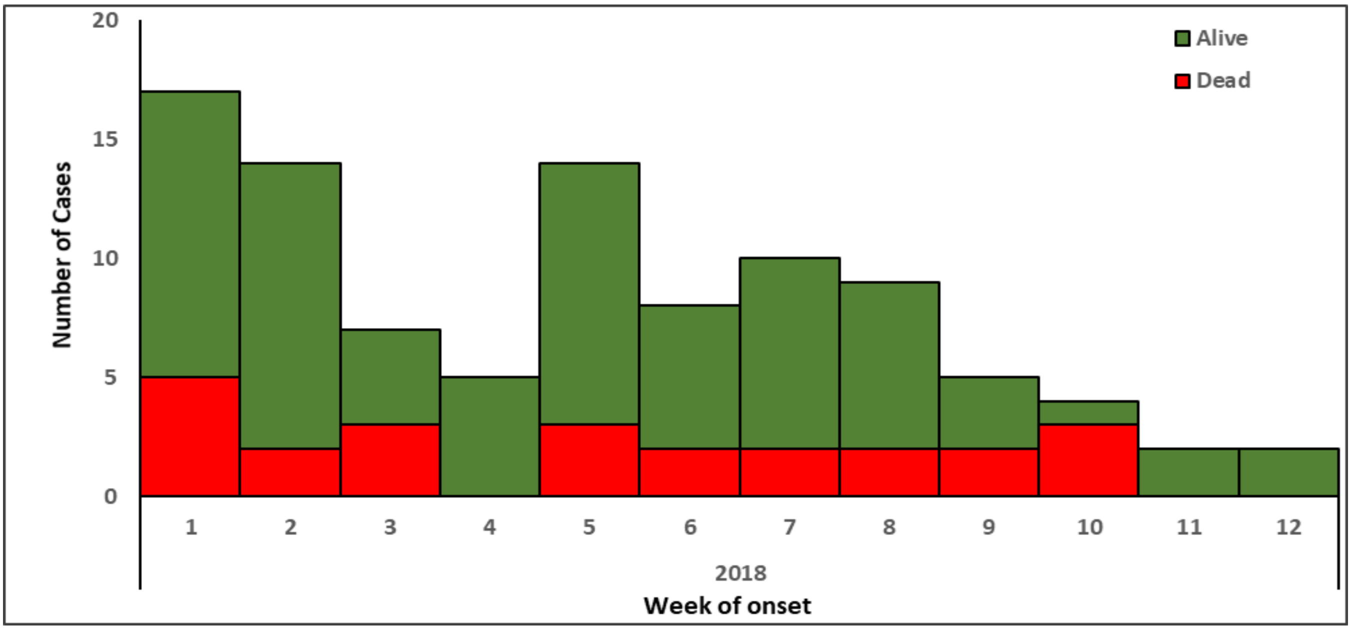

Figure 7: Epicurve of Lassa fever confirmed cases in Ondo State week 1-12, 2018

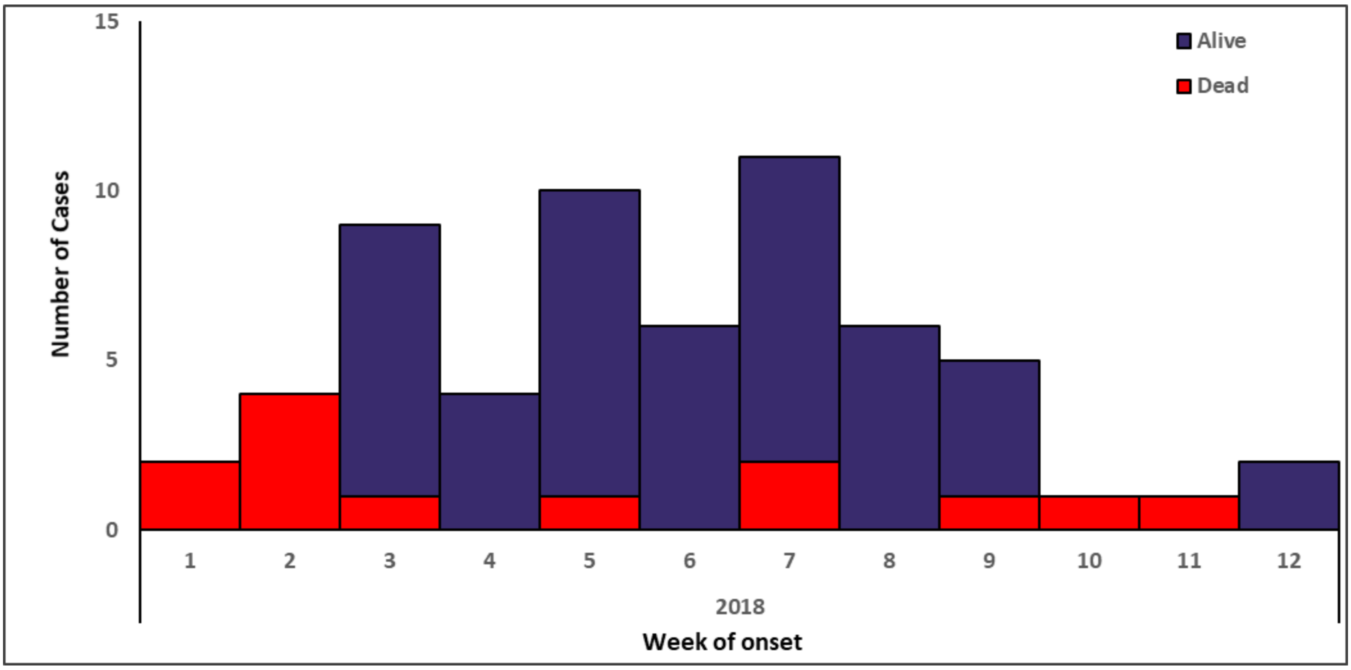

Figure 8: Epicurve of Lassa fever confirmed cases in Ebonyi State week 1-12, 2018

---

[i] *Suspected case describes any individual presenting with one or more of the following: malaise, fever, headache, sore throat, cough, nausea, vomiting, diarrhoea, myalgia, chest pain, hearing loss and either a. History of contact with excreta or urine of rodents b. History of contact with a probable or confirmed Lassa fever case within a period of 21 days of onset of symptoms OR Any person with inexplicable bleeding/hemorrhagia.*

[ii] *Any suspected case with laboratory confirmation (positive IgM antibody, PCR or virus isolation)*

[iii] *Any suspected case (see definition above) who died without collection of specimen for laboratory testing*

[iv] *"Active" means where there has been at least one confirmed case, and contacts within 21 days post exposure*

SITUATION REPORT
Nigeria Centre For Disease Control (NCDC)

www.ncdc.gov.ng PLOT 800 EBITU UKIWE STREET, JABI ABUJA, NIGERIA. TOLL FREE CALL: 0800-970000-10. E: info@ncdc.gov.ng @NCDCgov

TITLE:	2018 LASSA FEVER OUTBREAK IN NIGERIA
SERIAL NUMBER:	13
EPI-WEEK:	13
DATE:	01 April 2018

HIGHLIGHTS

- **In the reporting Week 13 (March 26- April 01, 2018) six new confirmed[ii] cases** were recorded from five States - Edo (2), Ondo (1), Bauchi (1), Plateau (1), and Abia (1) with **two new deaths** in confirmed cases from FCT (1) and Abia (1)
- From 1st January to 1st April 2018, a total of 1706 **suspected[i] cases** have been reported**. Of these, 400 were confirmed positive, 9 are probable,** 1273 are negative (not a case) and 24 are awaiting laboratory results (pending)
- Since the onset of the 2018 outbreak, there have been 142 deaths: 97 in positive-confirmed cases, 9 in probable cases and 36 in negative cases. Case Fatality Rate in confirmed cases is 24.3% -*Table 1*
- 20 states have recorded at least one confirmed case across 57 Local Government Areas (Edo, Ondo, Bauchi, Nasarawa, Ebonyi, Anambra, Benue, Kogi, Imo, Plateau, Lagos, Taraba, Delta, Osun, Rivers, FCT, Gombe, Ekiti, Kaduna and Abia). **Eight** states have exited the active phase of the outbreak while 12 States remain active[iv] - *Table 1/ Figure 1*
- In the reporting week 14, two new healthcare workers were affected with one death. **Twenty five* health care workers have been affected since the onset of the outbreak in eight states** – Ebonyi (15), Nasarawa (1), Kogi (2), Benue (1), Ondo (2) Edo (3) and Abia (1) with five deaths in Ebonyi (3) Kogi (1) and Abia (1)
- 81% of all confirmed cases are from Edo (42%) Ondo (23%) and Ebonyi (16%) states
- 30 cases are currently under treatment in treatment centres across nine states -Edo (9), Ebonyi (6), Bauchi (7), Ondo (5), Plateau (1), Osun (1) and Kogi (1)
- A total of 4274 contacts have been identified from 20 states. Of these 662 (15.0%) are currently being followed up, 3605 (84.8%) have completed 21 days follow up while 7(0.2%) were lost follow up. **27 (40%)** of the 67 contacts have tested positive from five states (Edo-12, Ondo-7, Ebonyi-3, Kogi -3 and Bauchi-1)
- WHO and NCDC has scaled up response at National and State levels
- National RRT team (NCDC staff and NFELTP residents) batch C continues response support in Ebonyi, Ondo, Edo, Bauchi and Taraba State
- National Lassa fever multi-partner multi-agency Emergency Operations Centre (EOC) continues to coordinate the response activities at all levels

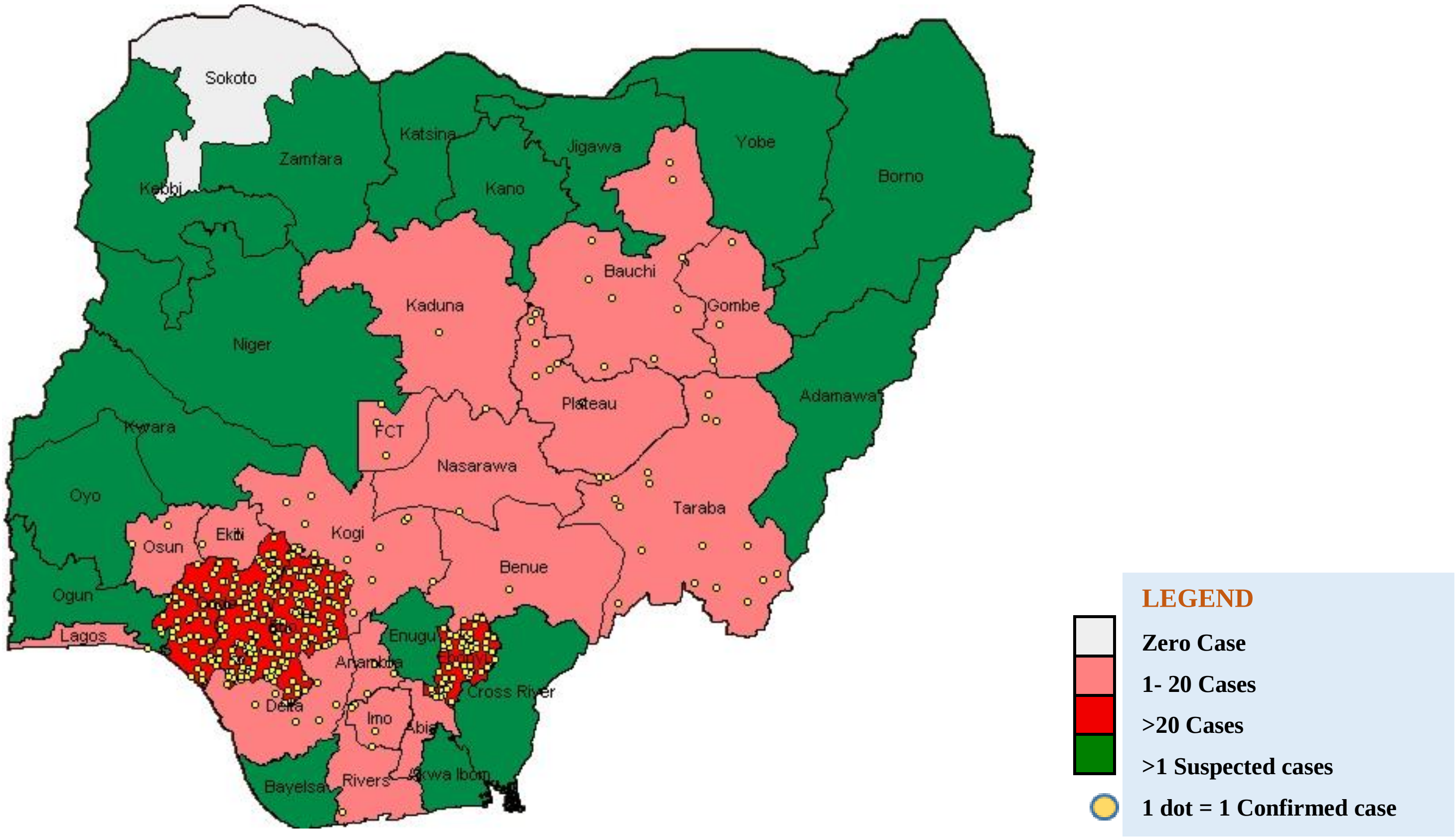

Figure 1. Distribution of Confirmed Lassa Fever cases in Nigeria as at 1st April, 2018

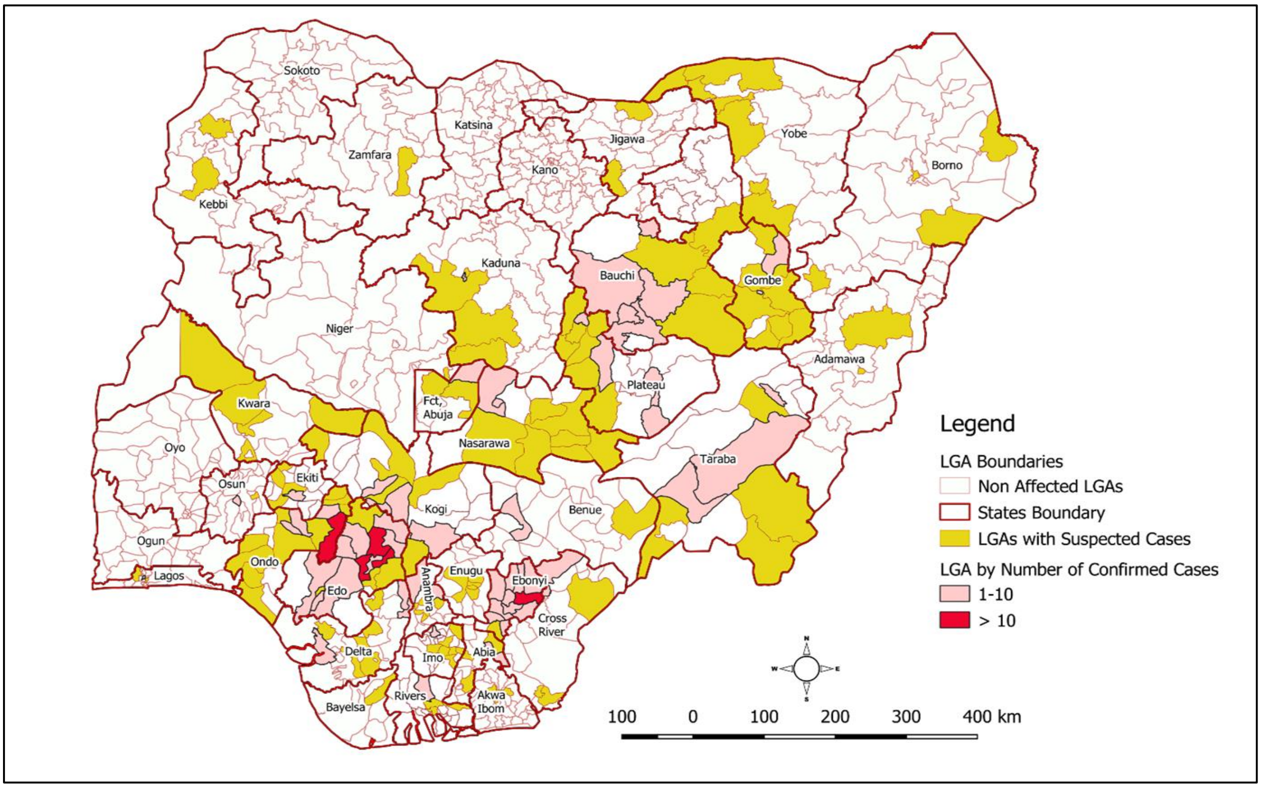

Figure 2. Distribution of Suspected and Confirmed Lassa Fever cases in Nigeria by LGA

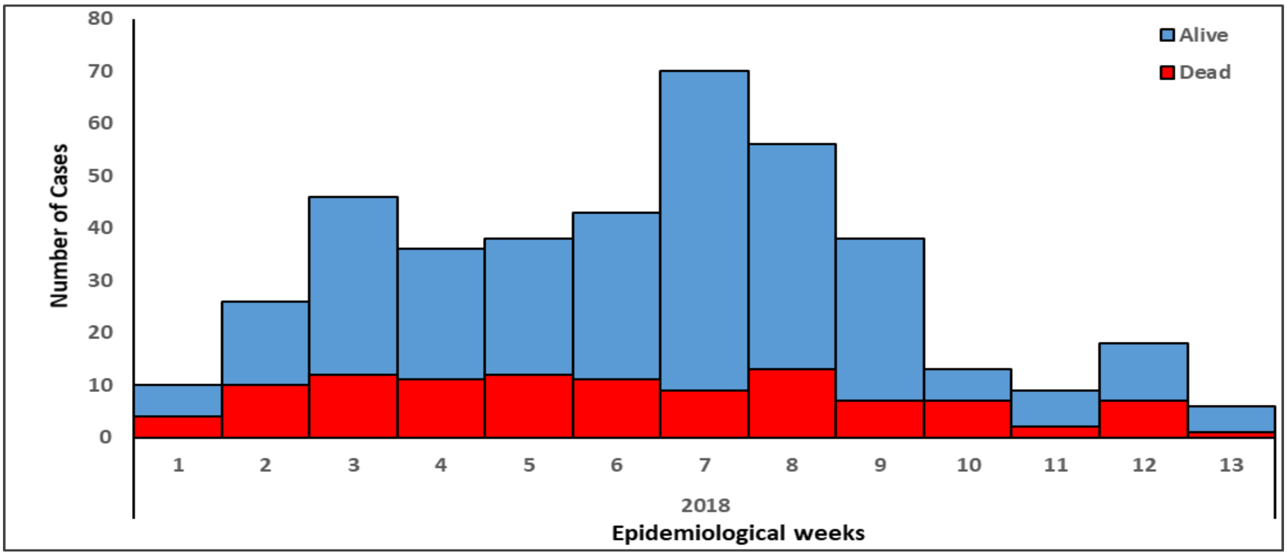

Figure 3. Confirmed (400) and Probable (9) Cases of Lassa fever by epidemiological week in Nigeria week 1- 13, 2018

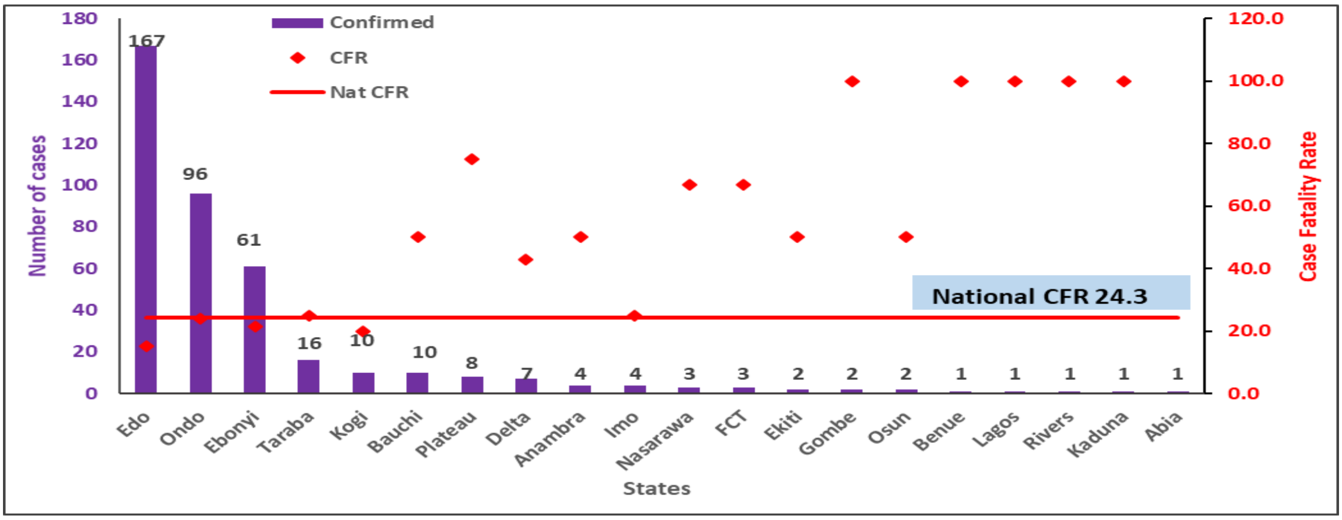

Figure 4. Confirmed Lassa fever cases in Nigeria with state specific Case Fatality Rates (CFR) as at 1st April 2018

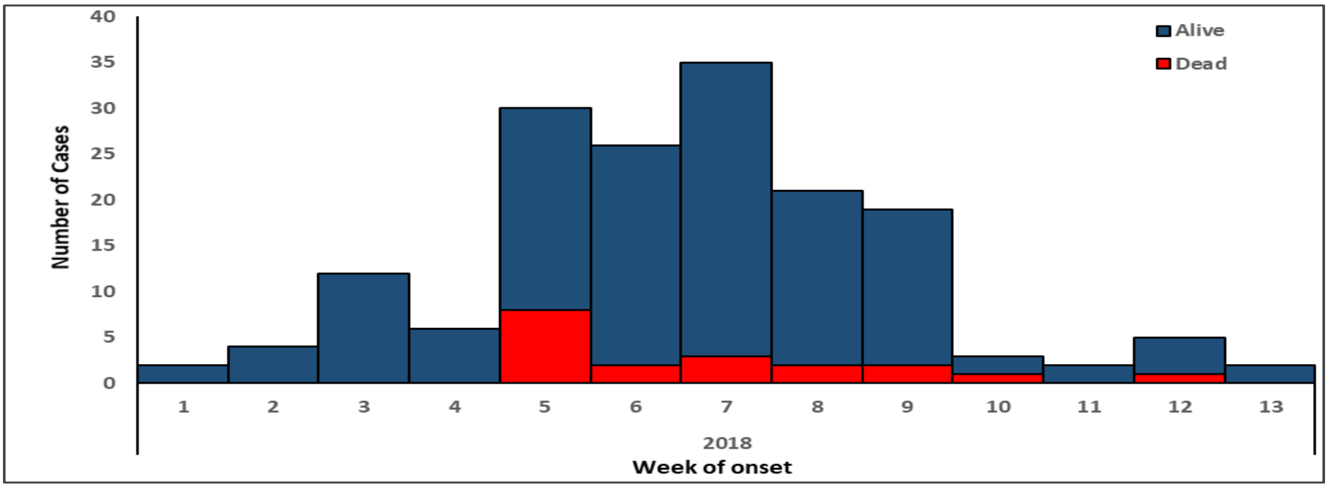

Figure 5: Epicurve of confirmed cases Lassa fever in Edo State week 1-13, 2018

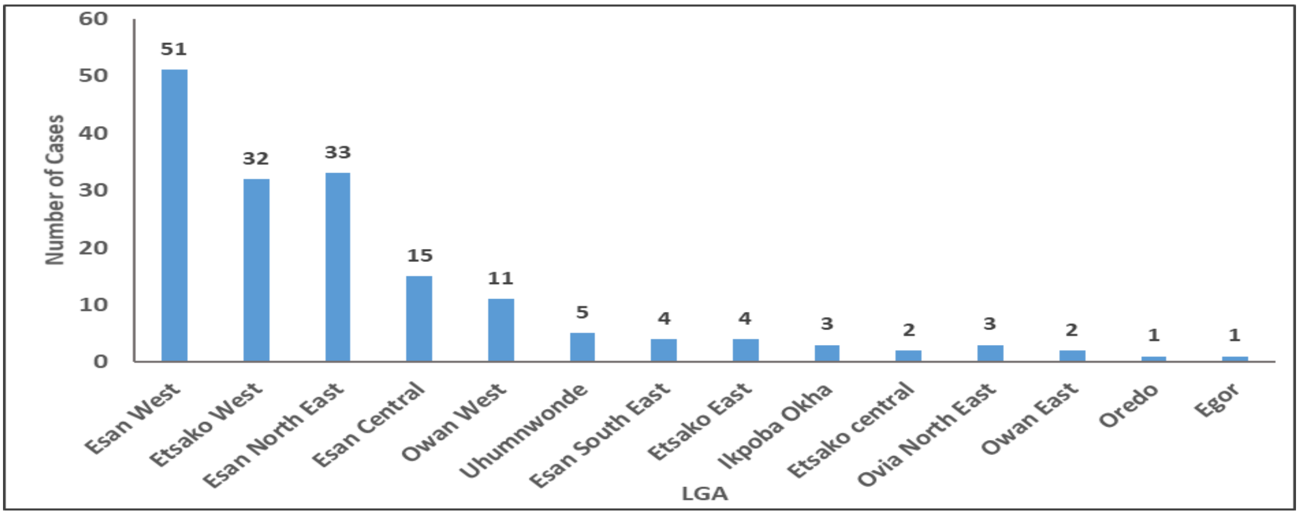

Figure 6: Distribution of Lassa fever confirmed cases in Edo State by LGA as at 1st April 2018

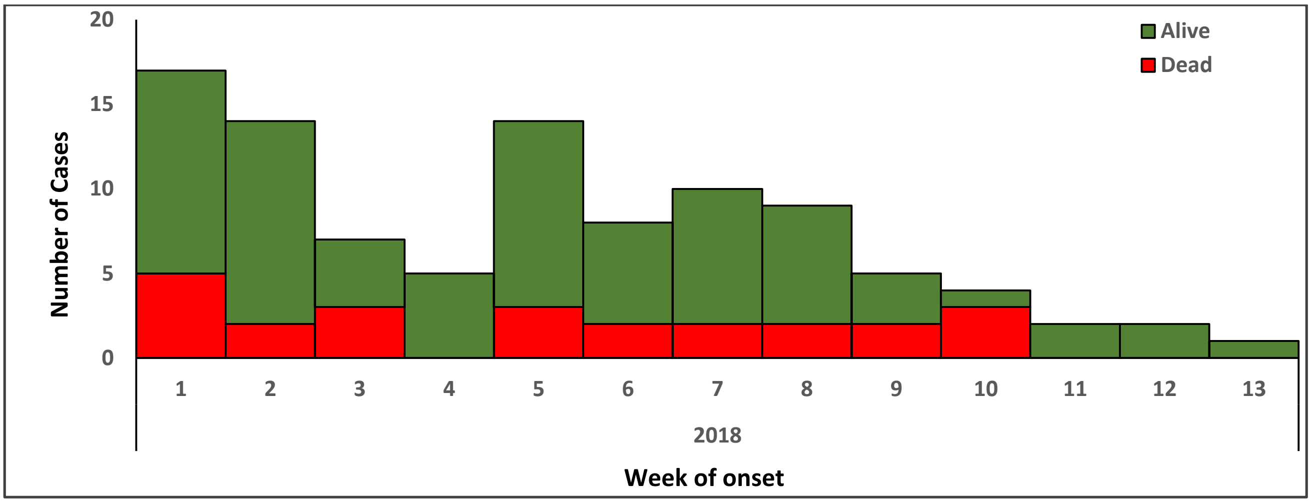

Figure 7: Epicurve of Lassa fever confirmed cases in Ondo State week 1- 13, 2018

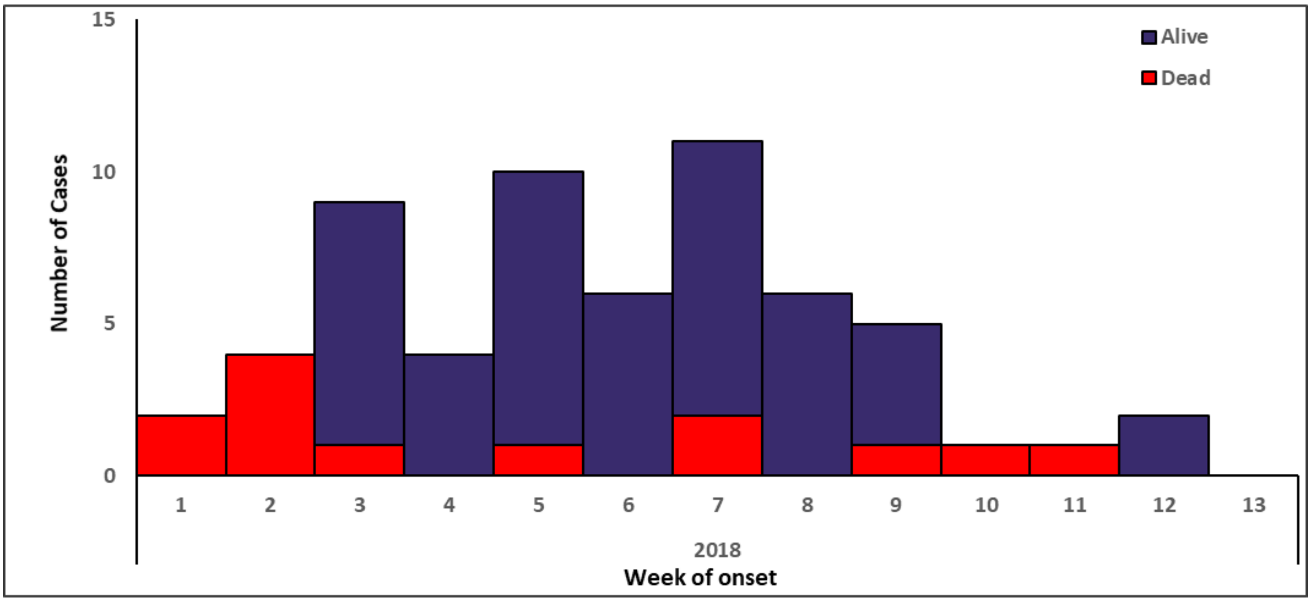

Figure 8: Epicurve of Lassa fever confirmed cases in Ebonyi State week 1-13, 2018

[i] *Suspected case describes any individual presenting with one or more of the following: malaise, fever, headache, sore throat, cough, nausea, vomiting, diarrhoea, myalgia, chest pain, hearing loss and either a. History of contact with excreta or urine of rodents b. History of contact with a probable or confirmed Lassa fever case within a period of 21 days of onset of symptoms OR Any person with inexplicable bleeding/hemorrhagia.*

[ii] *Any suspected case with laboratory confirmation (positive IgM antibody, PCR or virus isolation)*

[iii] *Any suspected case (see definition above) who died without collection of specimen for laboratory testing*

[iv] *"Active" means where there has been at least one confirmed case, and contacts within 21 days post exposure*

**Ebonyi state carried out data validation and reported backlog of 6 additional cases among Health care workers*

TITLE:	2018 LASSA FEVER OUTBREAK IN NIGERIA
SERIAL NUMBER:	14
EPI-WEEK:	14
DATE:	08 April 2018

HIGHLIGHTS

- **In the reporting Week 14 (April 02-08, 2018) eight new confirmed[ii] cases** were recorded from five States - Edo (3), Ondo (2), Ebonyi (1), Plateau (1), and Taraba (1) with **three new deaths** in confirmed cases from Ondo (1), Ebonyi (1) Taraba (1) and a backlog of an old death in a confirmed case from Kogi state.
- From 1st January to 8th April 2018, a total of 1781 **suspected[i] cases** have been reported from 20 states**. Of these, 408 were confirmed positive, 9 are probable,** 1351 are negative (not a case) and 13 are awaiting laboratory results (pending)
- Since the onset of the 2018 outbreak, there have been 101 deaths in confirmed cases, 9 in probable cases. Case Fatality Ratio in confirmed cases is 24.8%
- 20 states have recorded at least one confirmed case across 57 Local Government Areas (Edo, Ondo, Bauchi, Nasarawa, Ebonyi, Anambra, Benue, Kogi, Imo, Plateau, Lagos, Taraba, Delta, Osun, Rivers, FCT, Gombe, Ekiti, Kaduna and Abia). **Nine** states have exited the active phase of the outbreak while 11 States remain active[iv] - *Figure 1*
- In the reporting week 14, one new healthcare worker was affected in Ebonyi state with one death. **Twenty-seven* health care workers have been affected since the onset of the outbreak in seven states** –Ebonyi (16), Nasarawa (1), Kogi (2), Benue (1), Ondo (3), Edo (3) and Abia (1) with six deaths in Ebonyi (4), Kogi (1) and Abia (1)
- 81% of all confirmed cases are from Edo (42%) Ondo (23%) and Ebonyi (16%) states
- Fifteen cases are currently being managed in treatment centres across six states -Edo (4), Ebonyi (5), Ondo (4), Plateau (1), and Osun (1)
- A total of 4480 contacts have been identified from 20 states. Of these 658 (14.8%) are currently being followed up, 3815 (85%) have completed 21 days follow up while 7(0.2%) were lost follow up. 74 symptomatic contacts have been identified, of which **28 (38%)** have tested positive from five states (Edo-13, Ondo-8, Ebonyi-3, Kogi -3 and Bauchi-1)
- National RRT team (NCDC staff and NFELTP residents) batch D continues response in Abia state support
- National Lassa fever multi-partner multi-agency Emergency Operations Centre (EOC) continues to coordinate the response activities at all levels

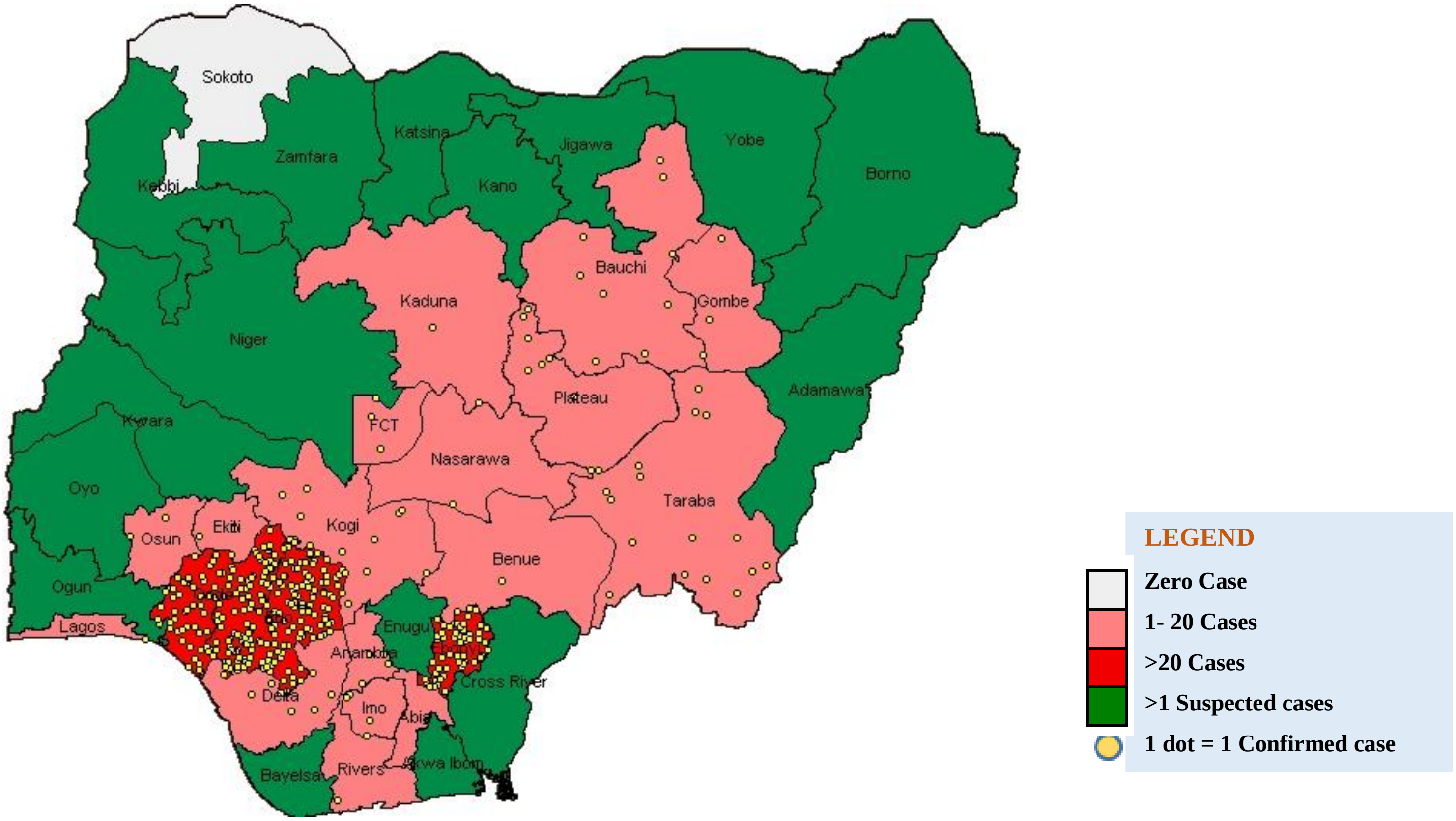

Figure 1. Distribution of Confirmed Lassa Fever cases in Nigeria as at 8th April, 2018

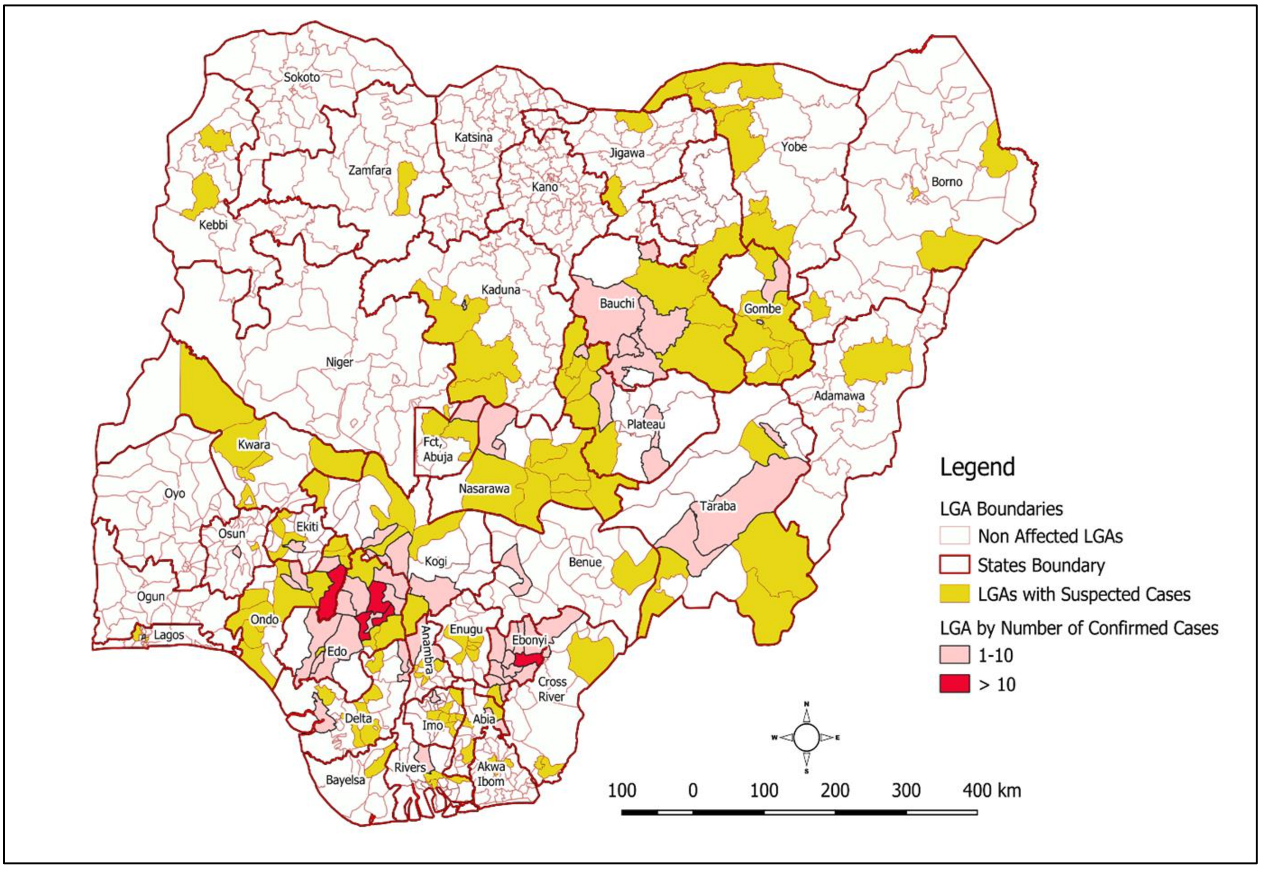

Figure 2. Distribution of Suspected and Confirmed Lassa Fever cases in Nigeria by LGA

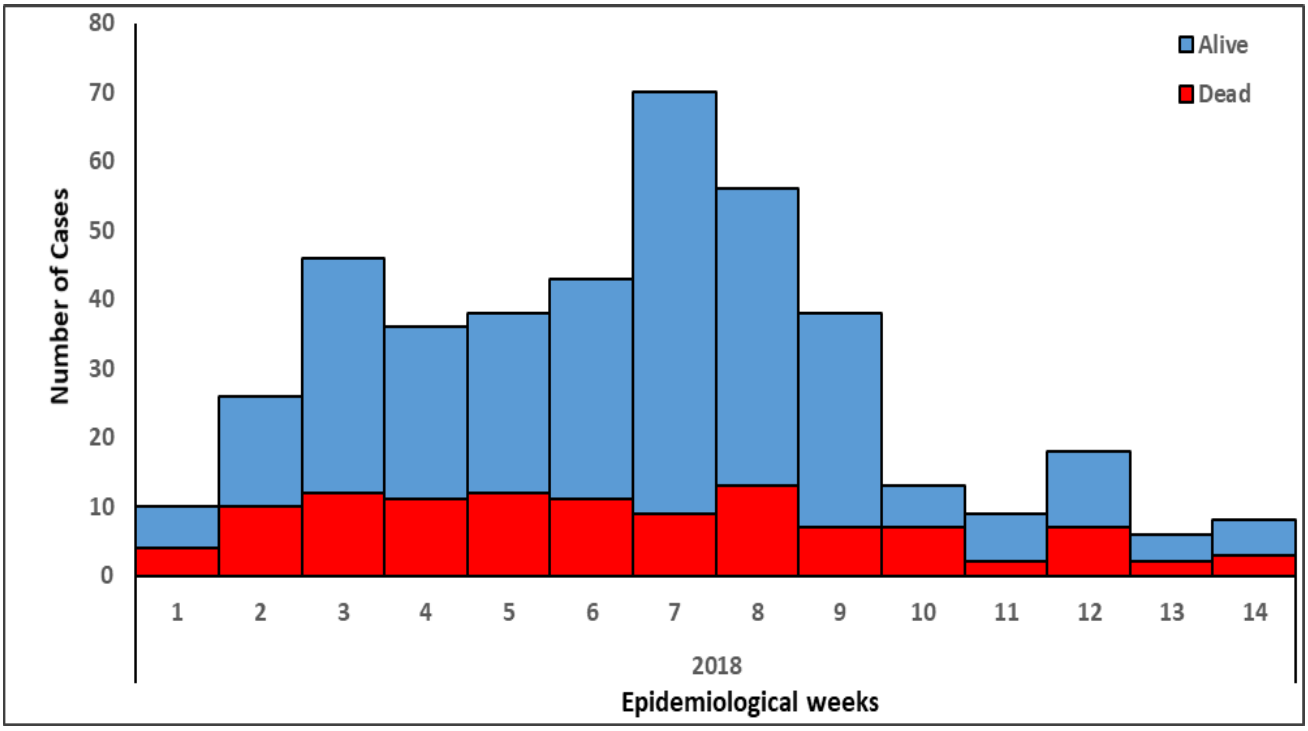

Figure 3. Confirmed (408) and Probable (9) Cases of Lassa fever by epidemiological week in Nigeria week 1- 14, 2018

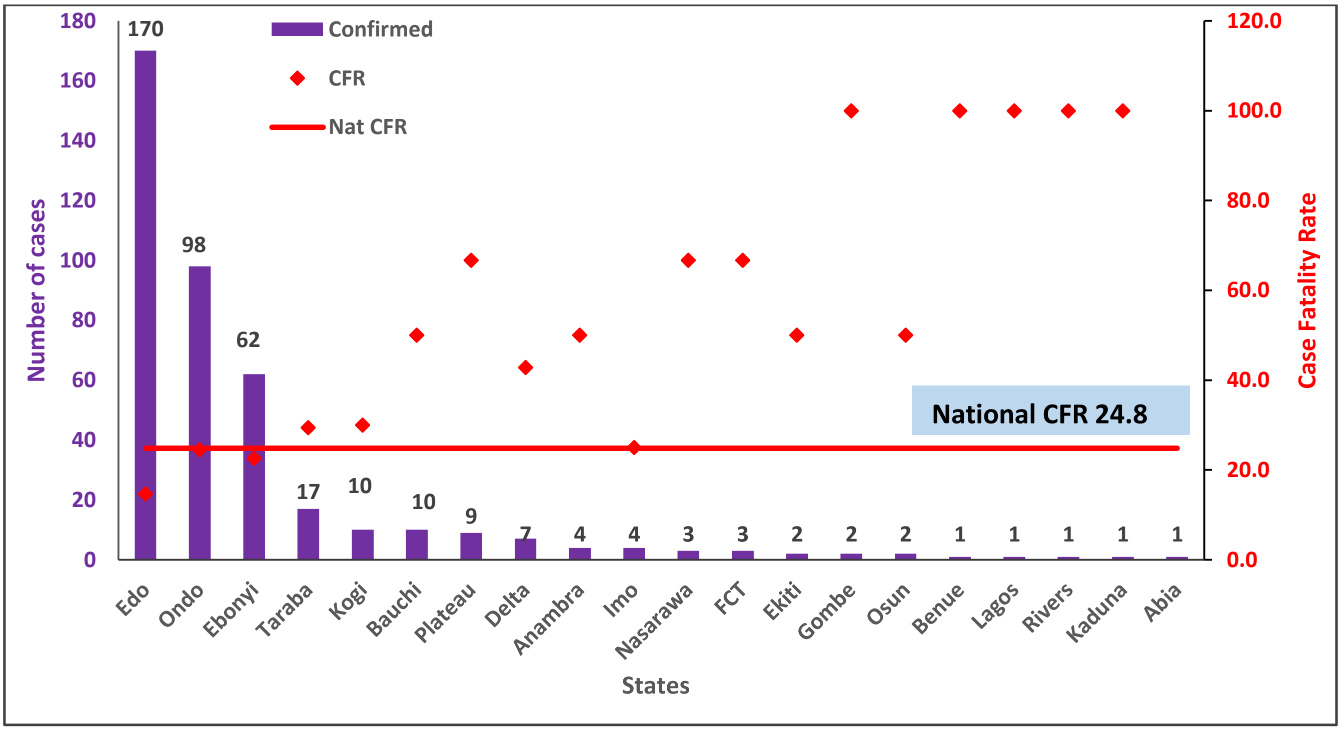

Figure 4. Confirmed Lassa fever cases in Nigeria with state specific Case Fatality Rates (CFR) as at 8th April 2018

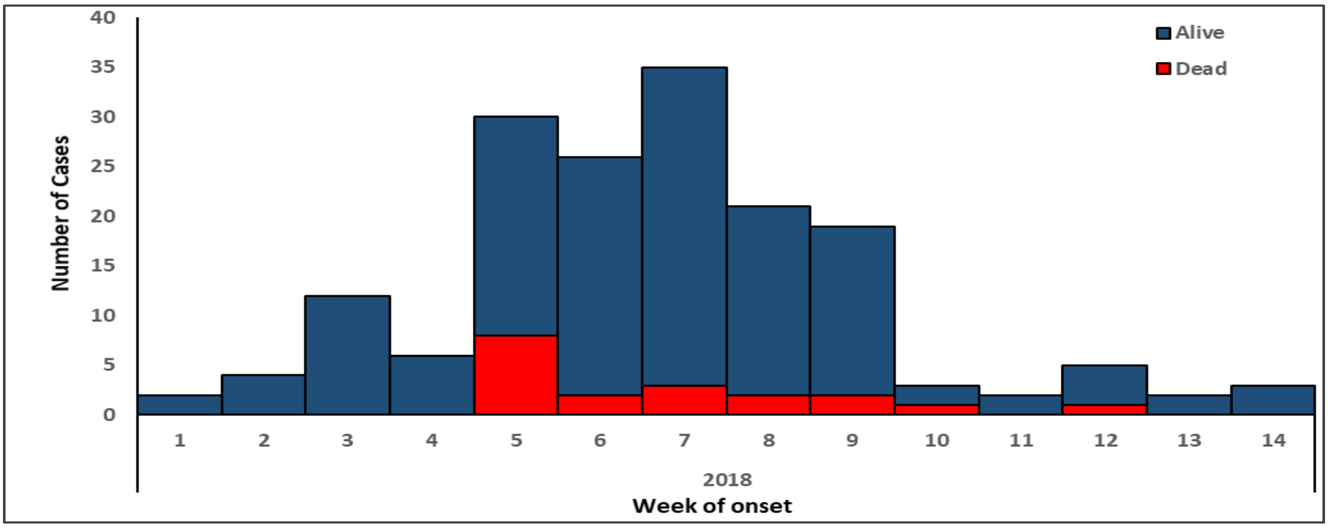

Figure 5: Epicurve of confirmed cases Lassa fever in Edo State week 1-14, 2018

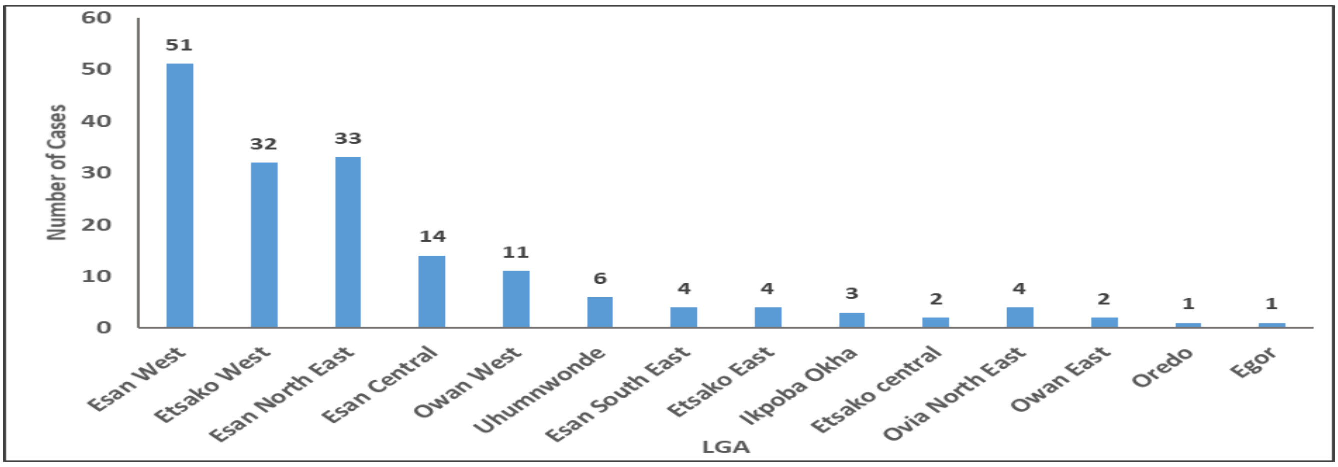

Figure 6: Distribution of Lassa fever confirmed cases in Edo State by LGA as at 8th April 2018

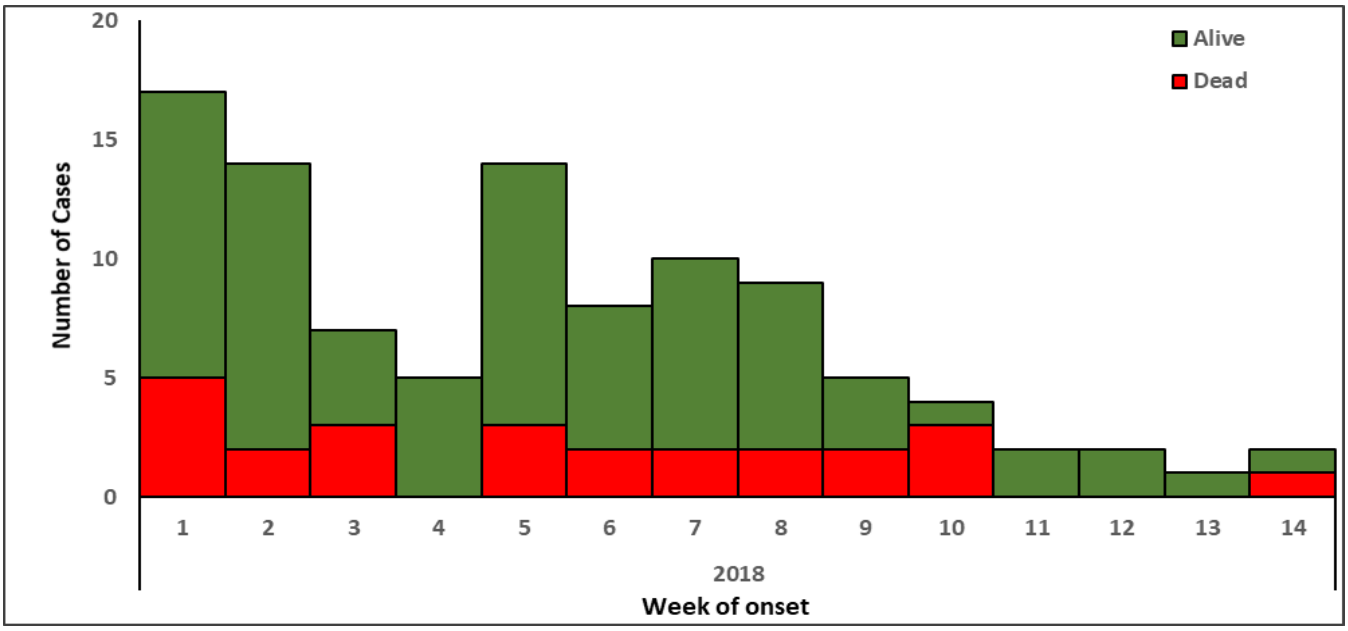

Figure 7: Epicurve of Lassa fever confirmed cases in Ondo State week 1- 14, 2018

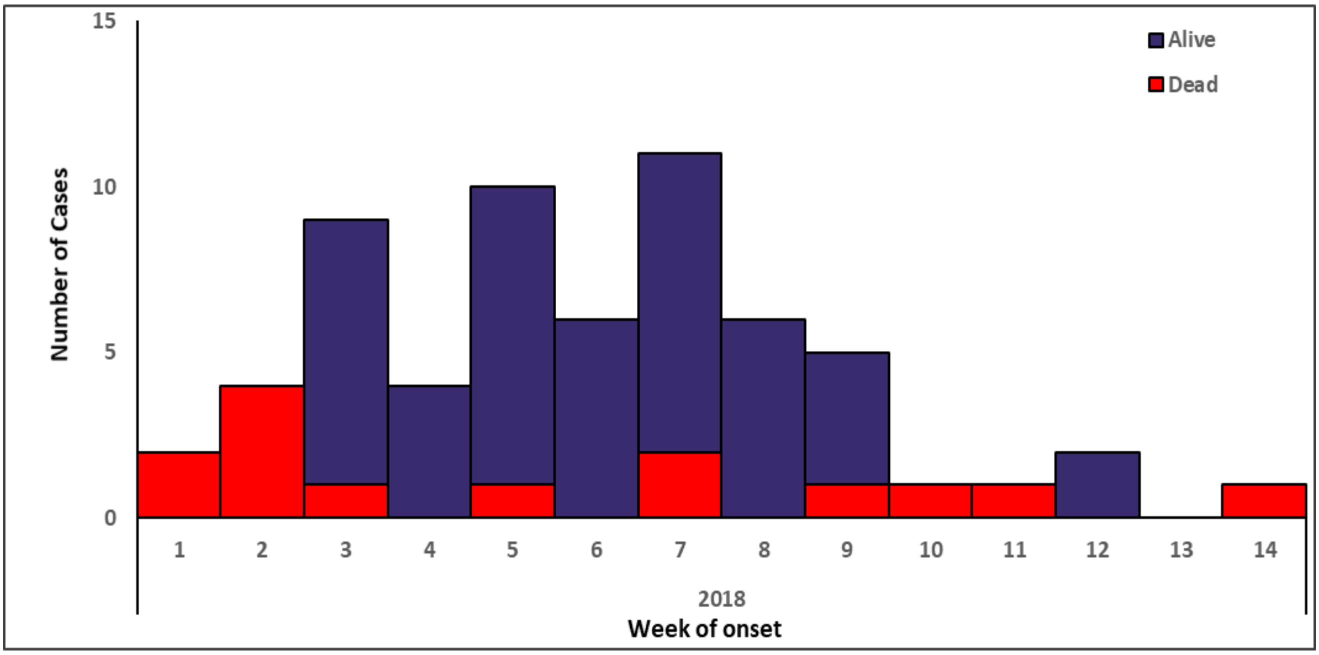

Figure 8: Epicurve of Lassa fever confirmed cases in Ebonyi State week 1-14, 2018

---

[i]*Suspected case describes any individual presenting with one or more of the following: malaise, fever, headache, sore throat, cough, nausea, vomiting, diarrhoea, myalgia, chest pain, hearing loss and either a. History of contact with excreta or urine of rodents b. History of contact with a probable or confirmed Lassa fever case within a period of 21 days of onset of symptoms OR Any person with inexplicable bleeding/hemorrhagia.*

[ii]*Any suspected case with laboratory confirmation (positive IgM antibody, PCR or virus isolation)*

[iii]*Any suspected case (see definition above) who died without collection of specimen for laboratory testing*

[iv] *"Active" means where there has been at least one confirmed case, and contacts within 21 days post exposure*

***** *Ondo state reported an old confirmed case in a Health care worker in February, though case was captured then but not as a health worker*

TITLE:	2018 LASSA FEVER OUTBREAK IN NIGERIA
SERIAL NUMBER:	15
EPI-WEEK:	15
DATE:	15 April 2018

HIGHLIGHTS

- **In the reporting Week 15 (April 09-15, 2018) five new confirmed**[il] **cases** were recorded from four States - Edo (2), Ebonyi (1), Kogi (1), and Adamawa (1) with **three new deaths** in confirmed cases from Ebonyi (1), Adamawa (1) and Kogi(1)
- **This is the lowest weekly case count since the first week of January 2018**
- From 1st January to 15th April 2018, a total of 1849 **suspected**[i] **cases** have been reported from 21 states**. Of these, 413 were confirmed positive, 9 are probable, 1422 are negative (not a case**) and 5 are awaiting laboratory results (pending)
- Since the onset of the 2018 outbreak, there have been 105 deaths in confirmed cases, 9 in probable cases. Case Fatality Ratio in confirmed cases is 25.4%
- 21 states have recorded at least one confirmed case across 70 Local Government Areas (Edo, Ondo, Bauchi, Nasarawa, Ebonyi, Anambra, Benue, Kogi, Imo, Plateau, Lagos, Taraba, Delta, Osun, Rivers, FCT, Gombe, Ekiti, Kaduna, Abia and Adamawa). **Ten** states have exited the active phase of the outbreak while eleven States remain active[iv] - *Figure 1*
- In the reporting week 15, no new healthcare worker was infected. **Twenty-seven health care workers have been affected since the onset of the outbreak in seven states** –Ebonyi (16), Nasarawa (1), Kogi (2), Benue (1), Ondo (3), Edo (3) and Abia (1) with eight deaths in Ebonyi (6), Kogi (1) and Abia (1)
- 81% of all confirmed cases are from Edo (42%) Ondo (23%) and Ebonyi (16%) states
- Eight cases are currently being managed in treatment centres across four states -Edo (4), Ebonyi (3) and Plateau (1). **The lowest since the beginning of the outbreak**
- A total of 4713 contacts have been identified from 21 states. Of these 603 (12.8%) are currently being followed up, 4152 (87%) have completed 21 days follow up while 7(0.2%) were lost follow up. 78 symptomatic contacts have been identified, of which **28 (36%)** have tested positive from five states (Edo-13, Ondo-8, Ebonyi-3, Kogi -3 and Bauchi-1)
- National RRT team (NCDC, UMB, WHO and NFELTP residents) batch D continues response in Abia state support
- National Lassa fever multi-partner multi-agency Emergency Operations Centre (EOC) continues to coordinate the response activities at all levels

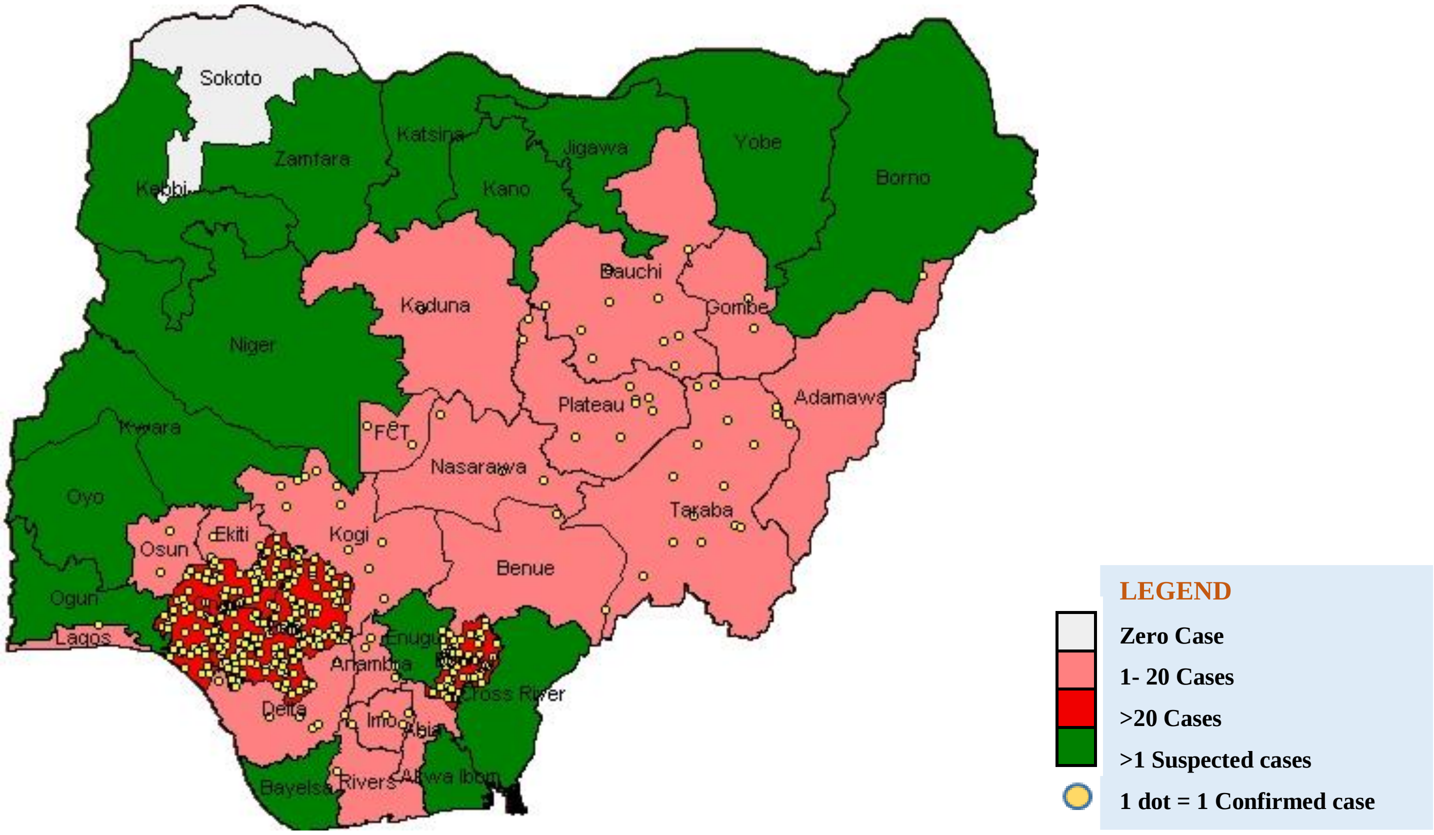

Figure 1. Distribution of Confirmed Lassa Fever cases in Nigeria as at 15th April, 2018

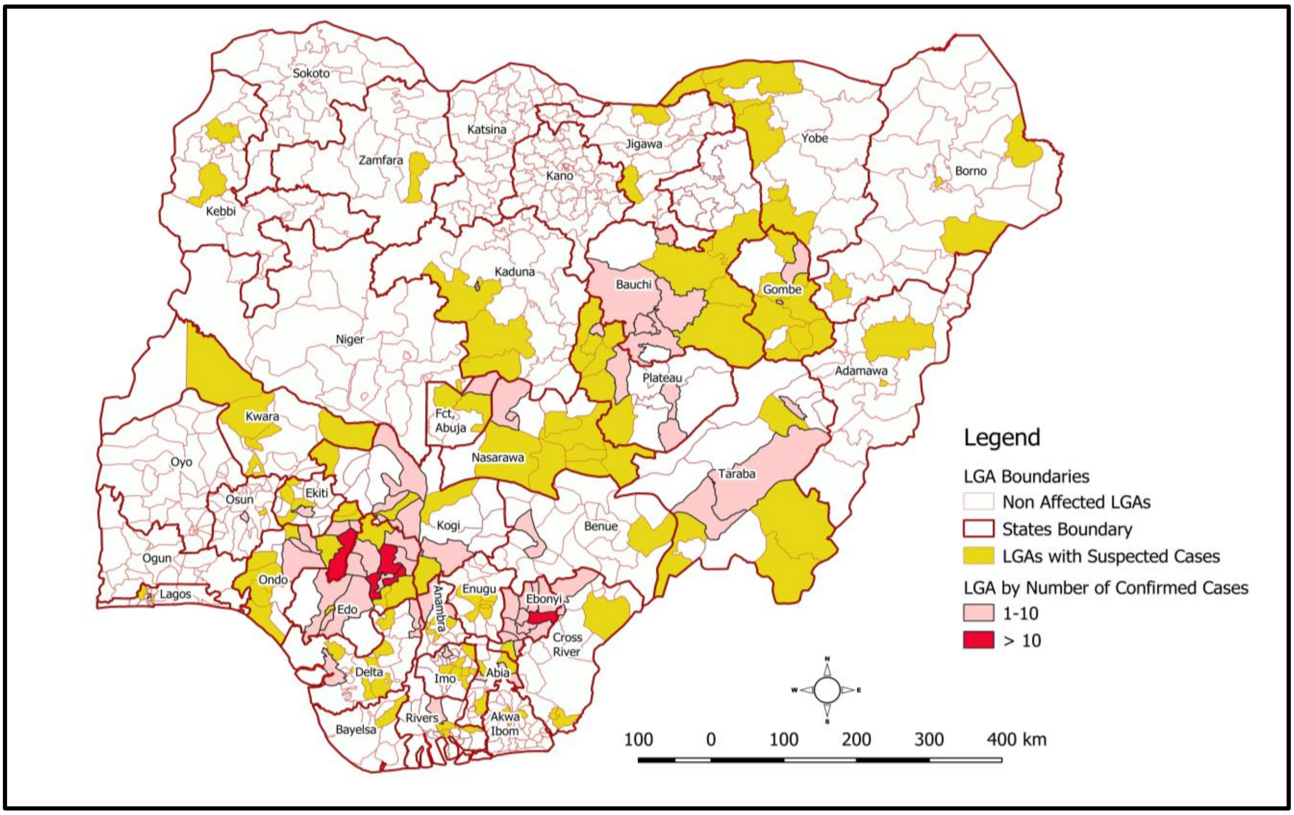

Figure 2. Distribution of Suspected and Confirmed Lassa Fever cases in Nigeria by LGA as at week 15

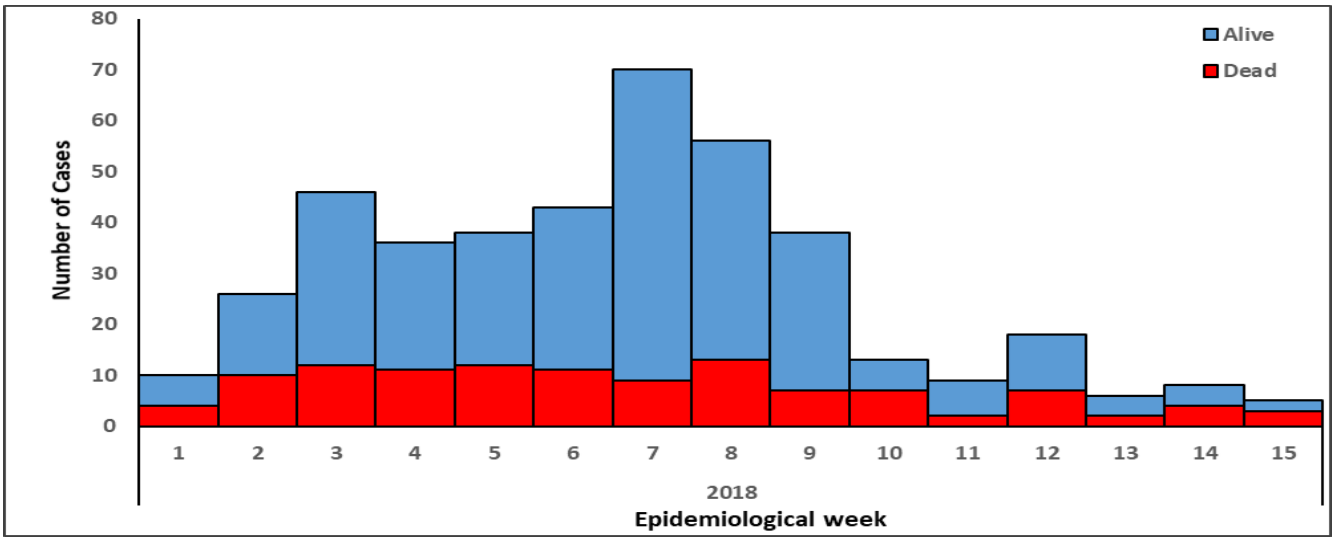

Figure 3. Confirmed (413) and Probable (9) Cases of Lassa fever by epidemiological week in Nigeria week 1- 15, 2018

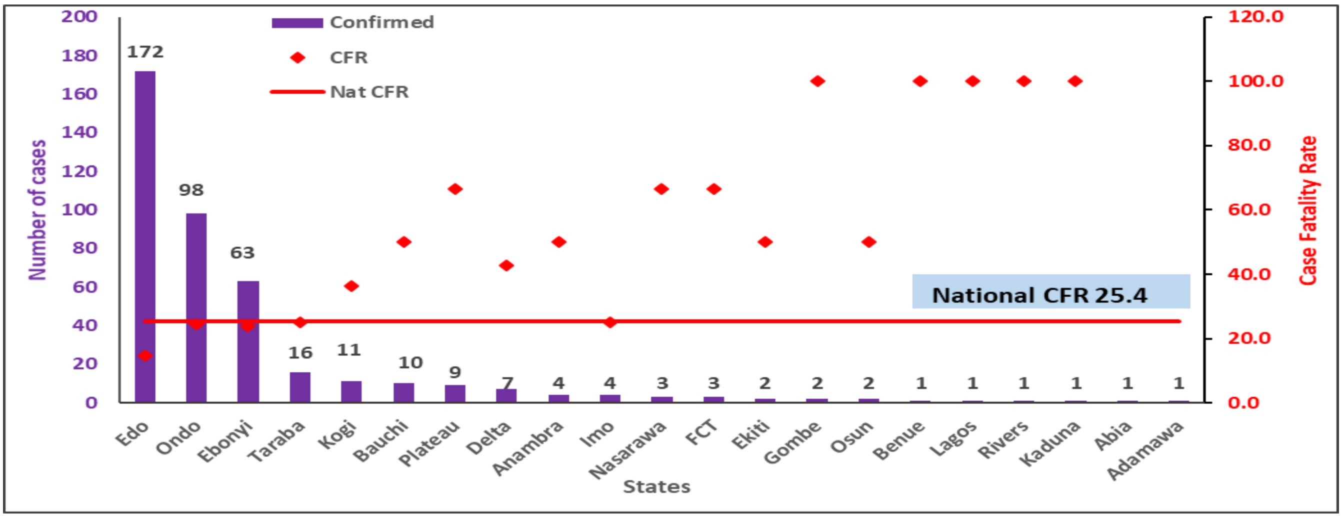

Figure 4. Confirmed Lassa fever cases in Nigeria with state specific Case Fatality Rates (CFR) as at 15th April 2018

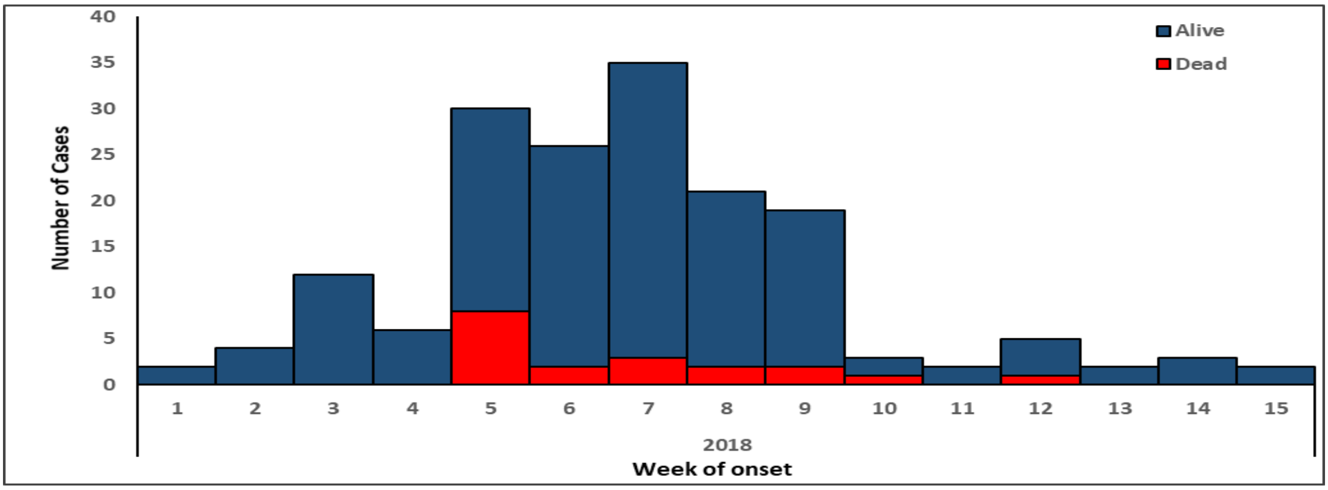

Figure 5: Epicurve of confirmed cases Lassa fever in Edo State week 1-15, 2018

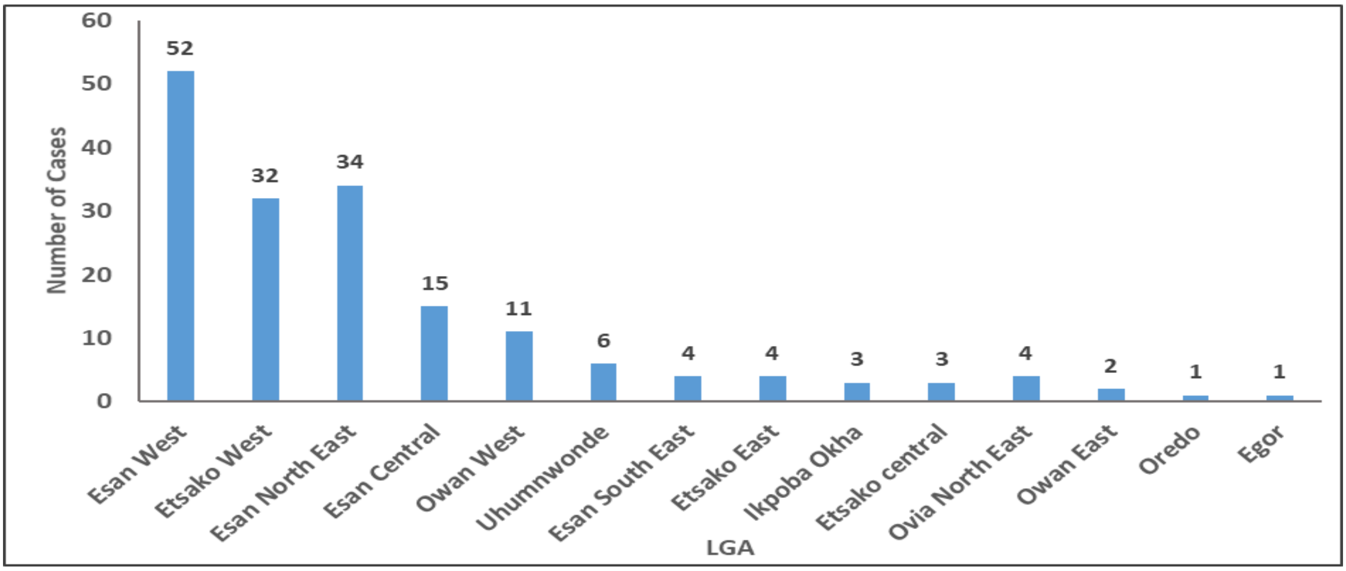

Figure 6: Distribution of Lassa fever confirmed cases in Edo State by LGA as at 15th April 2018

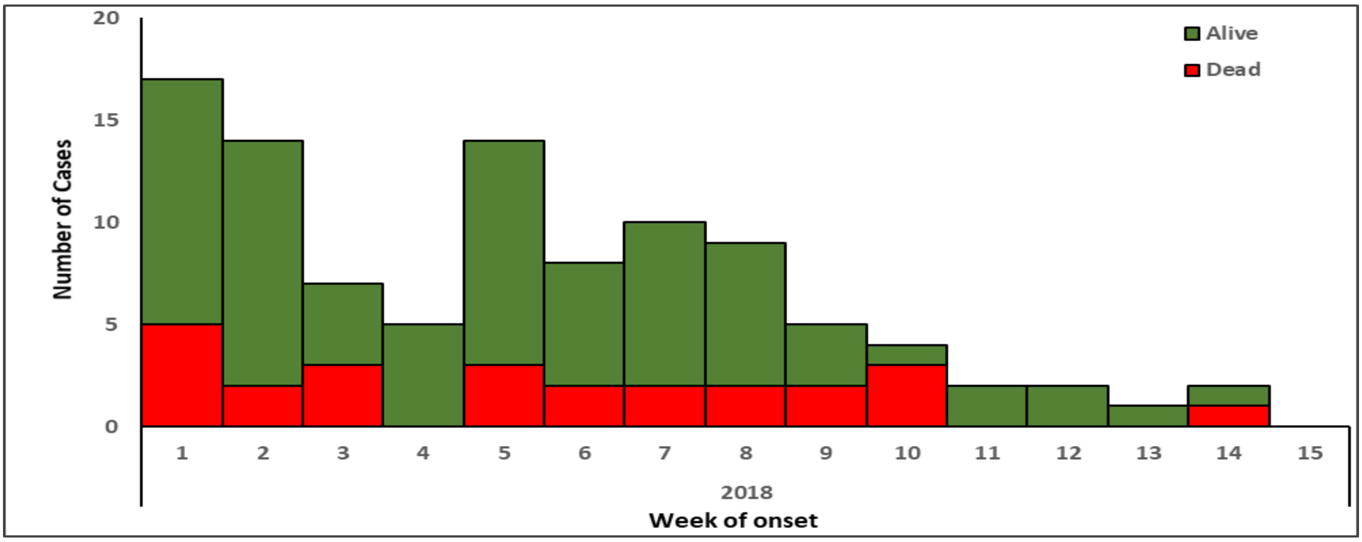

Figure 7: Epicurve of Lassa fever confirmed cases in Ondo State week 1- 15, 2018

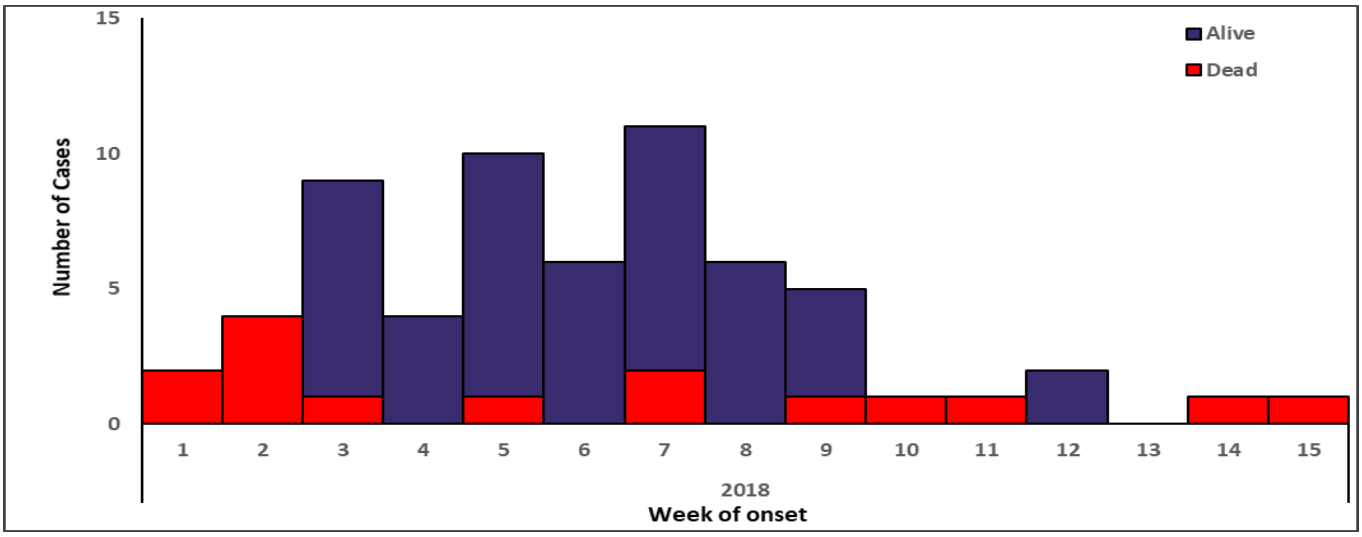

Figure 8: Epicurve of Lassa fever confirmed cases in Ebonyi State week 1-15, 2018

[i]*Suspected case describes any individual presenting with one or more of the following: malaise, fever, headache, sore throat, cough, nausea, vomiting, diarrhoea, myalgia, chest pain, hearing loss and either a. History of contact with excreta or urine of rodents b. History of contact with a probable or confirmed Lassa fever case within a period of 21 days of onset of symptoms OR Any person with inexplicable bleeding/hemorrhagia.*

[ii]*Any suspected case with laboratory confirmation (positive IgM antibody, PCR or virus isolation)*

[iii]*Any suspected case (see definition above) who died without collection of specimen for laboratory testing*

[iv] *"Active" means where there has been at least one confirmed case, and contacts within 21 days post exposure*

SITUATION REPORT
Nigeria Centre For Disease Control (NCDC)
NIGERIA CENTRE FOR DISEASE CONTROL
www.ncdc.gov.ng PLOT 800 EBITU UKIWE STREET, JABI ABUJA, NIGERIA. TOLL FREE CALL: 0800-970000-10. E: info@ncdc.gov.ng @NCDCgov

**TITLE:**	**2018 LASSA FEVER OUTBREAK IN NIGERIA**
**SERIAL NUMBER:**	**16**
**EPI-WEEK:**	**16**
**DATE:**	**22nd April 2018**

HIGHLIGHTS

- **In the reporting Week 16 (April 16-22, 2018) Edo state reported one new confirmed[ii] case** with **no new death**
- **This is the lowest weekly case count since the first week of January 2018**
- From 1st January to 22nd April 2018, a total of 1865 **suspected[i] cases** have been reported from 21 states**.** Of these, **416+ were confirmed positive, 9 are probable, 1439 are negative** (not a case) and 1 are awaiting laboratory results (pending)
- Since the onset of the 2018 outbreak, there have been 105 deaths in confirmed cases, 9 in probable cases. Case Fatality Rate in confirmed cases is 25.2%
- 21 states have recorded at least one confirmed case across 70 Local Government Areas (Edo, Ondo, Bauchi, Nasarawa, Ebonyi, Anambra, Benue, Kogi, Imo, Plateau, Lagos, Taraba, Delta, Osun, Rivers, FCT, Gombe, Ekiti, Kaduna, Abia and Adamawa). **Twelve** states have exited the active phase of the outbreak while nine States remain active[iv] - *Figure 1*
- In the reporting week 16, **one** new healthcare worker was infected in a private health facility in Edo state. **Thirty-seven* health care workers have been affected since the onset of the outbreak in eight states** –Ebonyi (16), Edo (12), Ondo (3), Kogi (2), Benue (1), Nasarawa (1), Taraba (1), and Abia (1) with eight deaths in Ebonyi (6), Kogi (1) and Abia (1)
- 81% of all confirmed cases are from Edo (42%) Ondo (23%) and Ebonyi (16%) states
- Three cases are currently being managed in treatment centres in Edo state. **The lowest since the beginning of the outbreak**
- A total of 4906 contacts have been identified from 21 states. Of these 420 (8.6%) are currently being followed up, 4476 (91.2%) have completed 21 days follow up while 7(0.2%) were lost follow up. 78 symptomatic contacts have been identified, of which **28 (36%)** have tested positive from five states (Edo-13, Ondo-8, Ebonyi-3, Kogi -3 and Bauchi-1)
- National RRT team (NCDC, WHO and NFELTP residents) batch D continues response in Plateau state support
- National Lassa fever multi-partner multi-agency Emergency Operations Centre (EOC) continues to coordinate the response activities at all levels, **however response has been de-escalated** due to continued decline of cases over the past 9 weeks

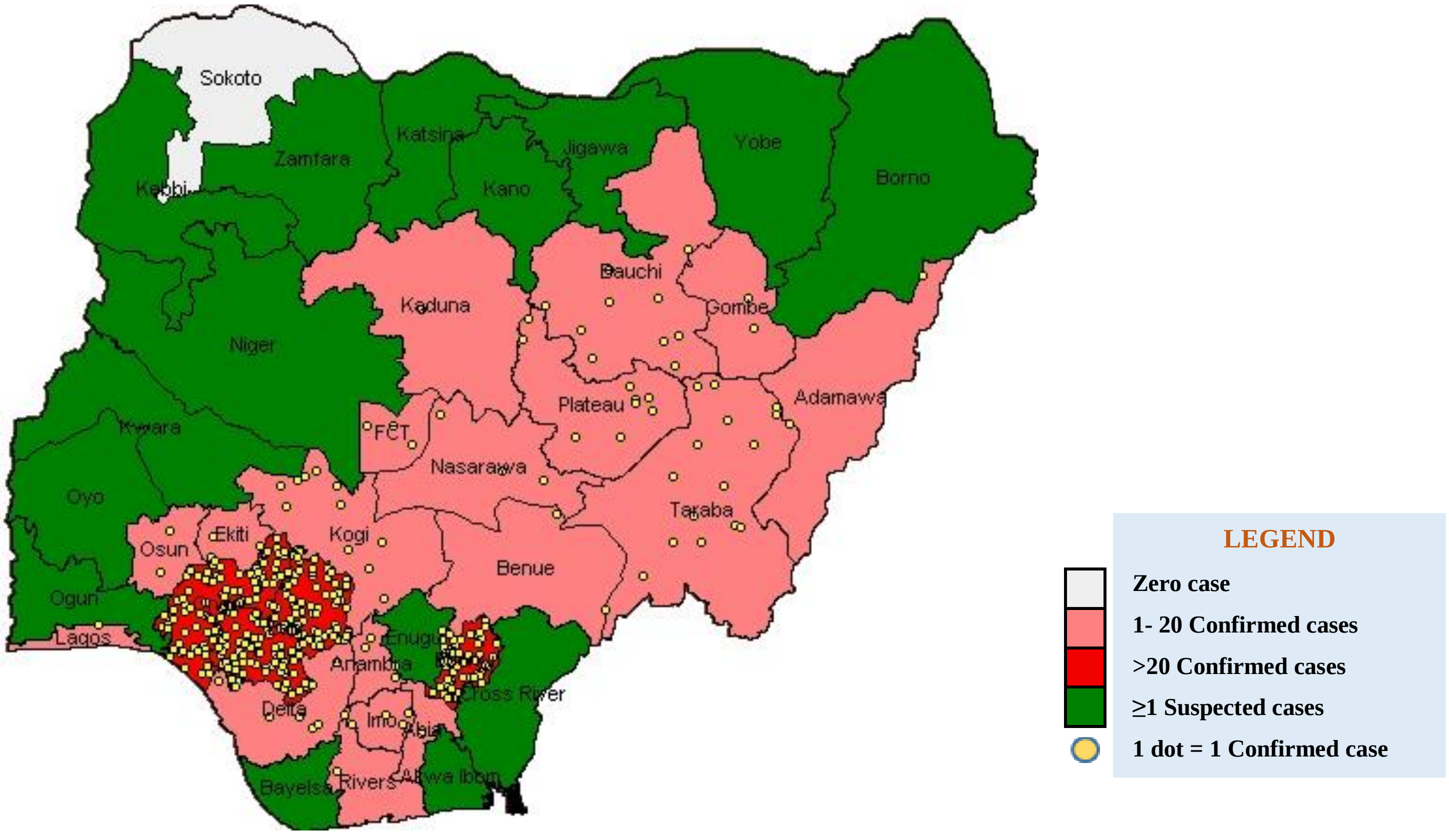

Figure 1. Distribution of Confirmed Lassa Fever cases in Nigeria as at 22nd April, 2018

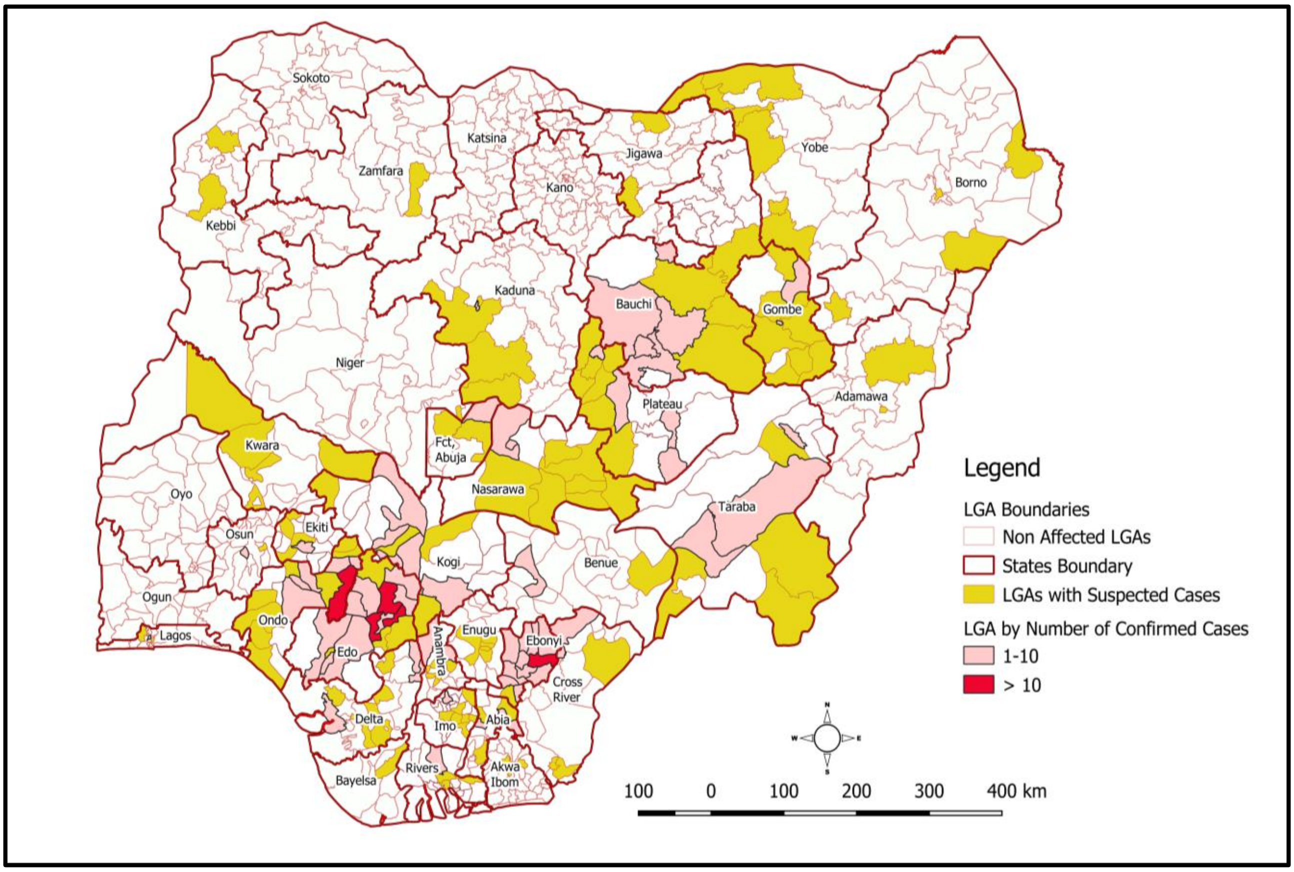

Figure 2. Distribution of Suspected and Confirmed Lassa Fever cases in Nigeria by LGA

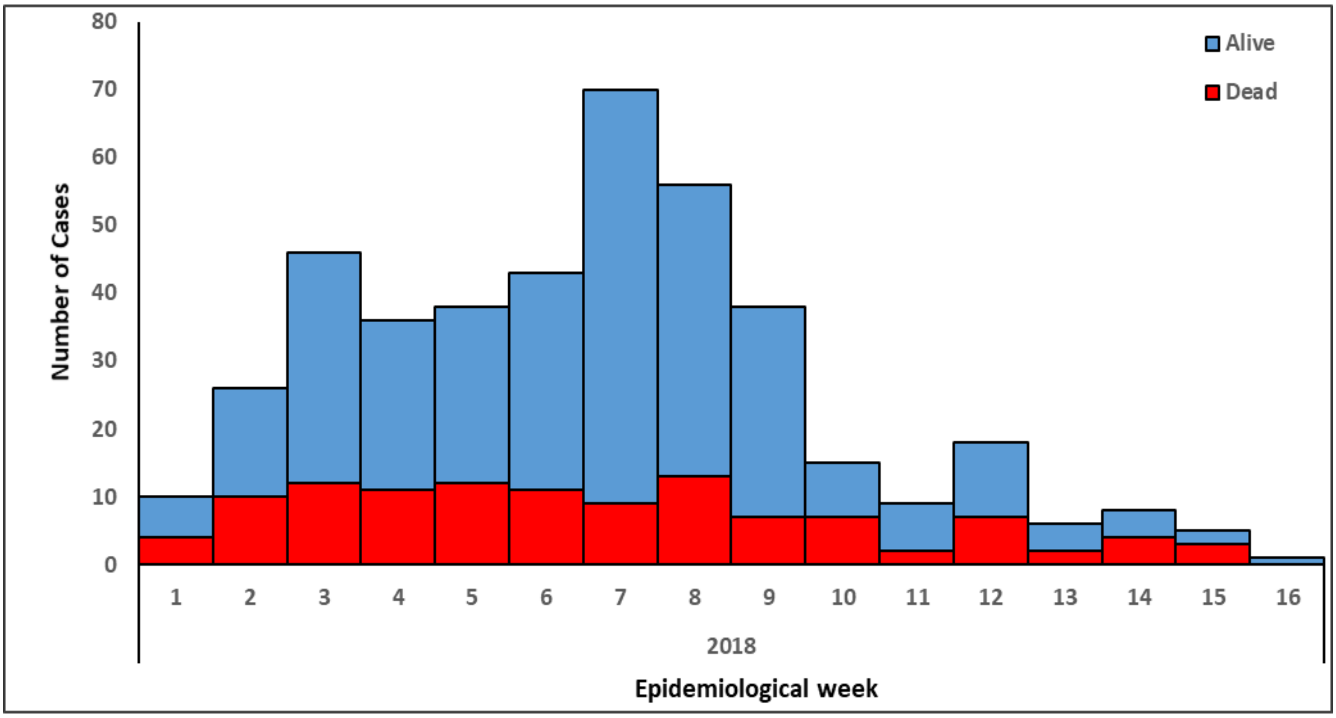

Figure 3. Confirmed (416) and Probable (9) Cases of Lassa fever by epidemiological week in Nigeria week 1- 16, 2018

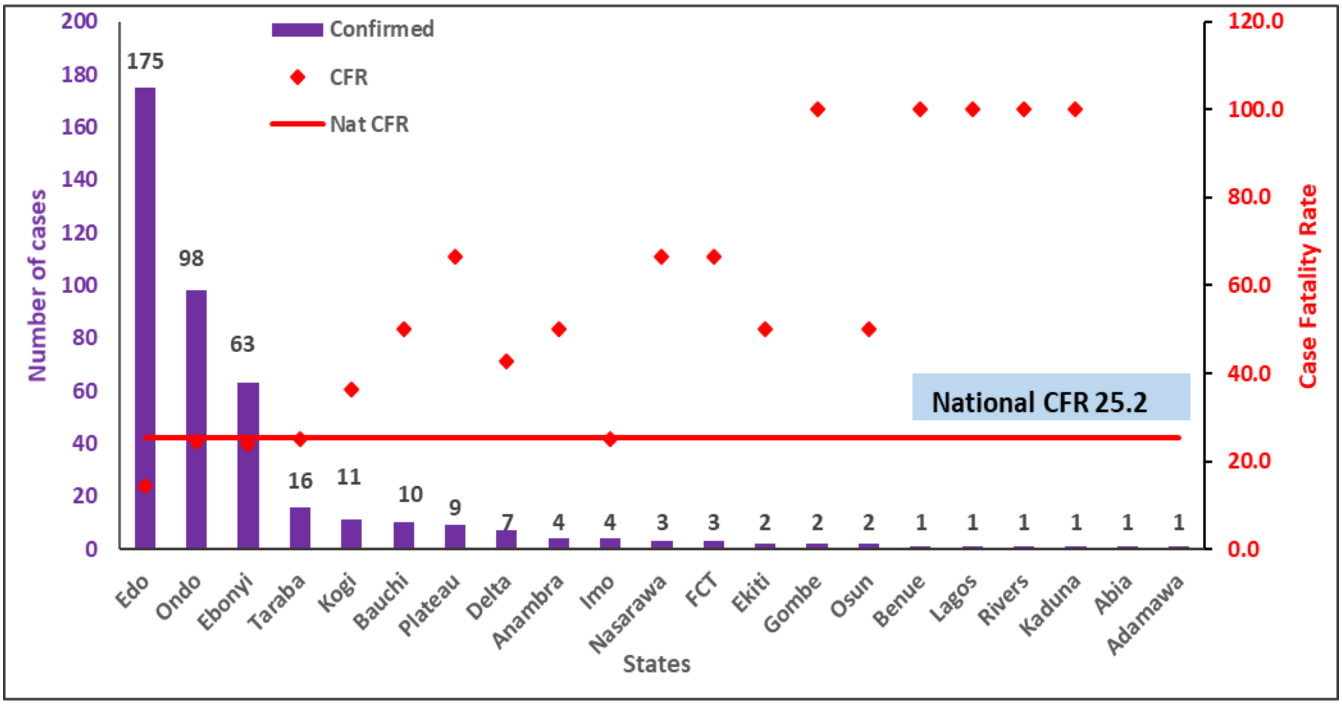

Figure 4. Confirmed Lassa fever cases in Nigeria with state specific Case Fatality Rates (CFR) as at 22nd April 2018

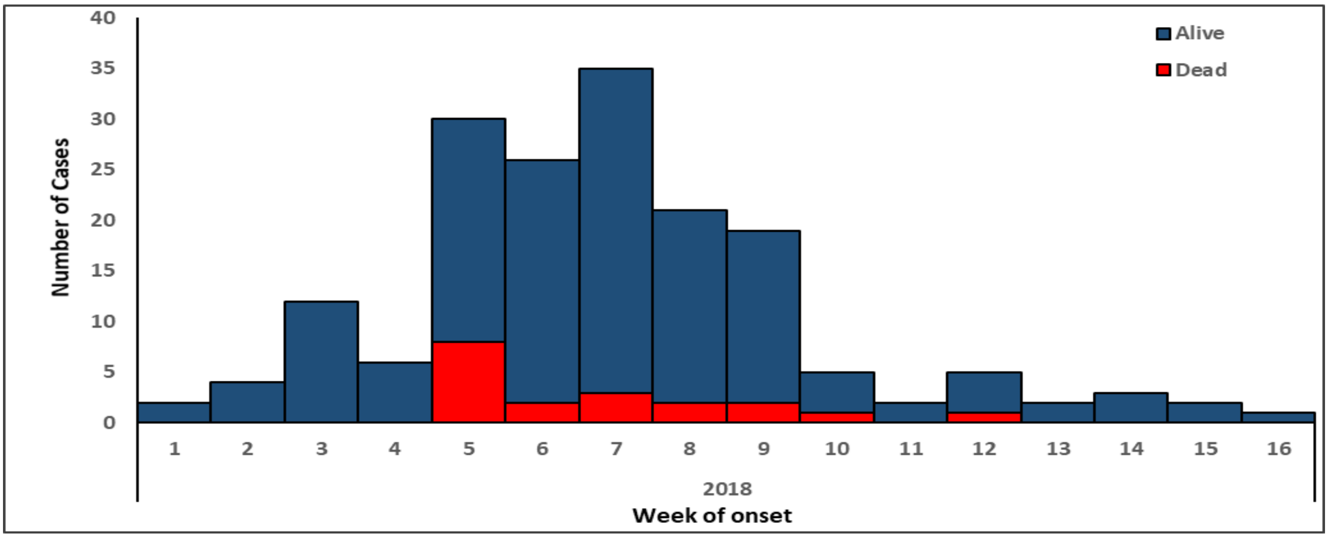

Figure 5: Epicurve of confirmed cases Lassa fever in Edo State week 1-16, 2018

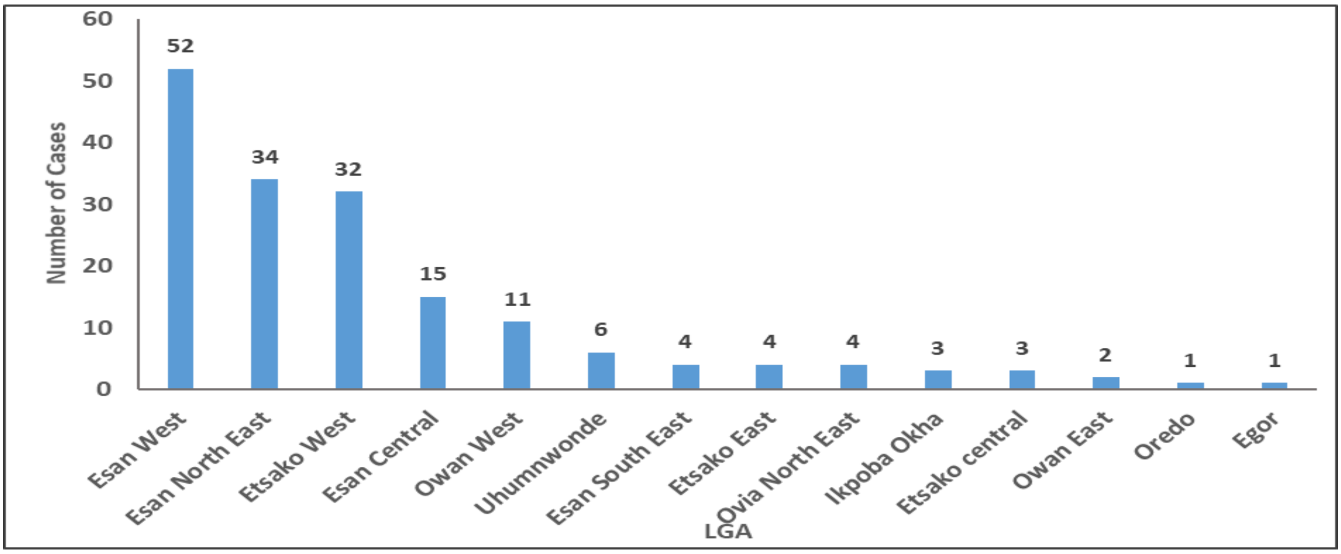

Figure 6: Distribution of Lassa fever confirmed cases in Edo State by LGA as at 22nd April 2018

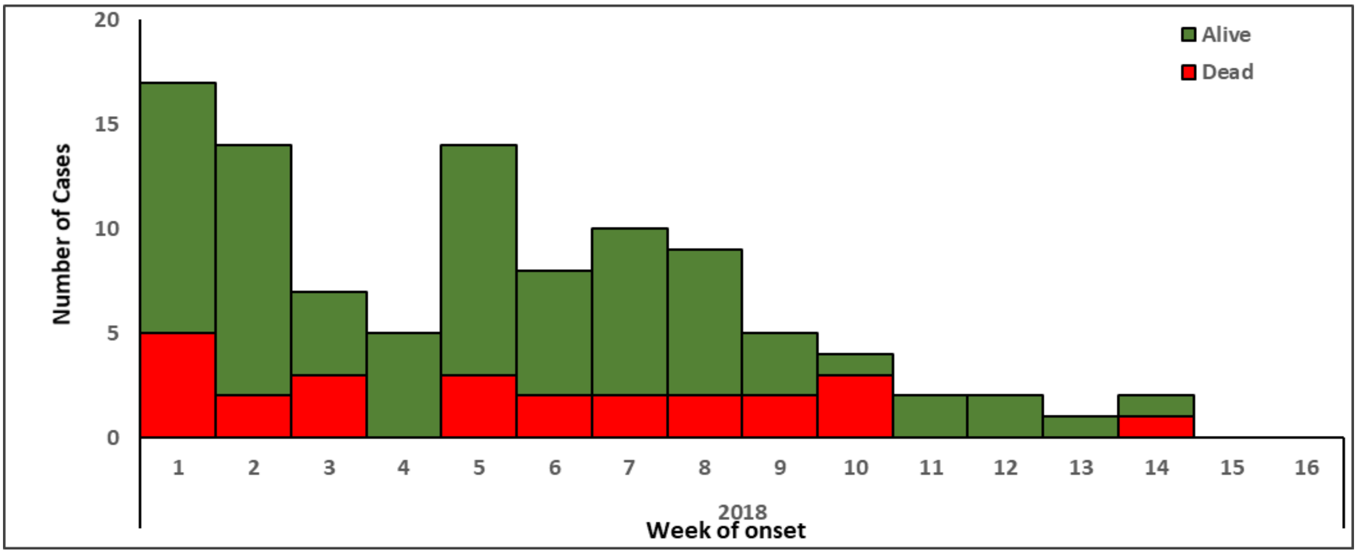

Figure 7: Epicurve of Lassa fever confirmed cases in Ondo State week 1- 16, 2018

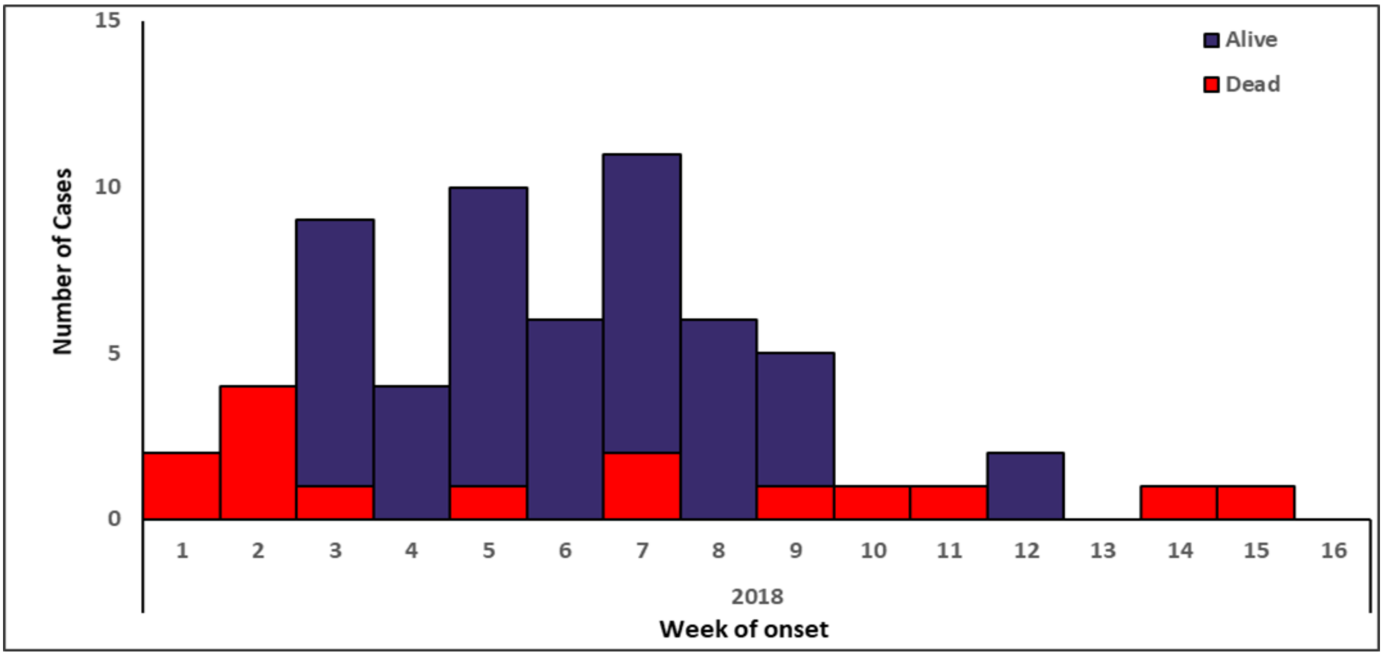

Figure 8: Epicurve of Lassa fever confirmed cases in Ebonyi State week 1-16, 2018

---

[i] *Suspected case describes any individual presenting with one or more of the following: malaise, fever, headache, sore throat, cough, nausea, vomiting, diarrhoea, myalgia, chest pain, hearing loss and either a. History of contact with excreta or urine of rodents b. History of contact with a probable or confirmed Lassa fever case within a period of 21 days of onset of symptoms OR Any person with inexplicable bleeding/hemorrhagia.*

[ii] *Any suspected case with laboratory confirmation (positive IgM antibody, PCR or virus isolation)*

iiiAny *suspected case (see definition above) who died without collection of specimen for laboratory testing*

[iv] *"Active" means where there has been at least one confirmed case, and contacts within 21 days post exposure*

+Edo state data validation revealed 2 additional retrospective cases in week 10
*** Nine retrospective health care workers infection from both public and private health facilities reported from Edo state and one from Taraba state**

SITUATION REPORT
Nigeria Centre For Disease Control (NCDC)
www.ncdc.gov.ng PLOT 800 EBITU UKIWE STREET, JABI ABUJA, NIGERIA. TOLL FREE CALL: 0800-970000-10. E: info@ncdc.gov.ng @NCDCgov

TITLE:	2018 LASSA FEVER OUTBREAK IN NIGERIA
SERIAL NUMBER:	17
EPI-WEEK:	17
DATE:	29th April 2018

HIGHLIGHTS

- **In the reporting Week 17 (April 23-29, 2018) four new confirmed[ii] cases** were reported from three states -Edo(1), Ondo(1) and Taraba (2) with **one new death** in Edo state
- From 1st January to 29th April 2018, a total of 1878 **suspected[i] cases** have been reported from 21 states**.** Of these, **420 were confirmed positive, 10* are probable, 1460 are <u>negative</u>** (not a case) and 1 is awaiting laboratory result (pending)
- Since the onset of the 2018 outbreak, there have been 106 deaths in confirmed cases, 10 in probable cases. Case Fatality Rate in confirmed cases is 25.2%
- 21 states have recorded at least one confirmed case across 70 Local Government Areas (Edo, Ondo, Bauchi, Nasarawa, Ebonyi, Anambra, Benue, Kogi, Imo, Plateau, Lagos, Taraba, Delta, Osun, Rivers, FCT, Gombe, Ekiti, Kaduna, Abia and Adamawa). **Fifteen** states have exited the active phase of the outbreak while six States remain active[iv] - *Figure 1*
- In the reporting week 17, **no** new healthcare worker was infected. **Thirty-seven health care workers have been affected since the onset of the outbreak in eight states** –Ebonyi (16), Edo (12), Ondo (3), Kogi (2), Benue (1), Nasarawa (1), Taraba (1), and Abia (1) with eight deaths in Ebonyi (6), Kogi (1) and Abia (1)
- 81% of all confirmed cases are from Edo (42%) Ondo (23%) and Ebonyi (16%) states
- Four cases are currently being managed in three treatment centres across the country
- A total of 4997 contacts have been identified from 21 states. Of these 286 (5.7%) are currently being followed up, 4704 (94.1%) have completed 21 days follow up while 7(0.2%) were lost follow up. 81 symptomatic contacts have been identified, of which **28 (36%)** have tested positive from five states (Edo-13, Ondo-8, Ebonyi-3, Kogi -3 and Bauchi-1)
- National RRT team (NCDC, WHO and NFELTP residents) batch D continues to support response in Plateau, Kogi and Adamawa states
- National Lassa fever multi-partner multi-agency Emergency Operations Centre (EOC) continues to coordinate the response activities at all levels, **however response has been de-escalated** due to continual decline of cases over the past 10 weeks

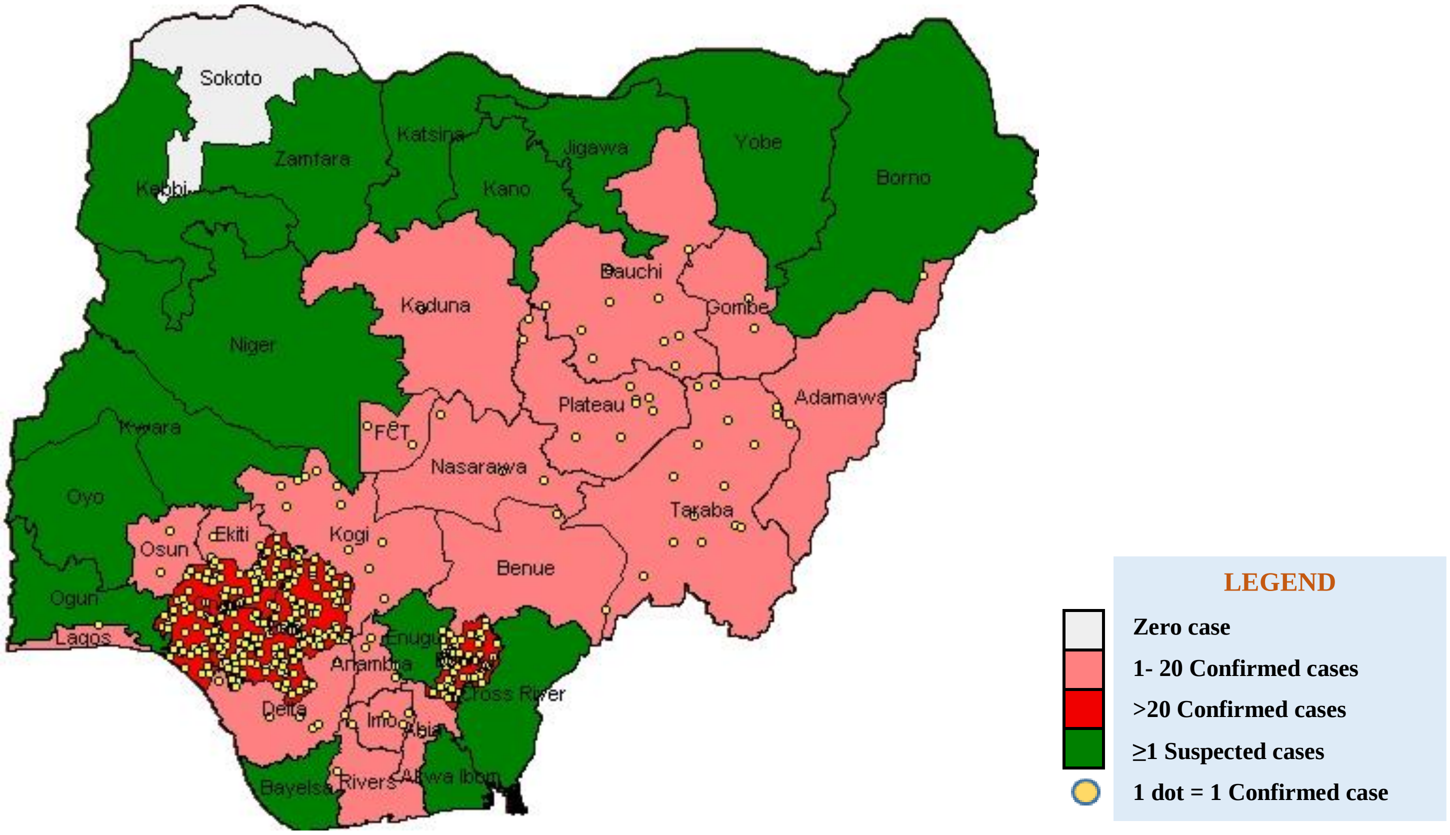

Figure 1. Distribution of Confirmed Lassa Fever cases in Nigeria as at 29th April, 2018

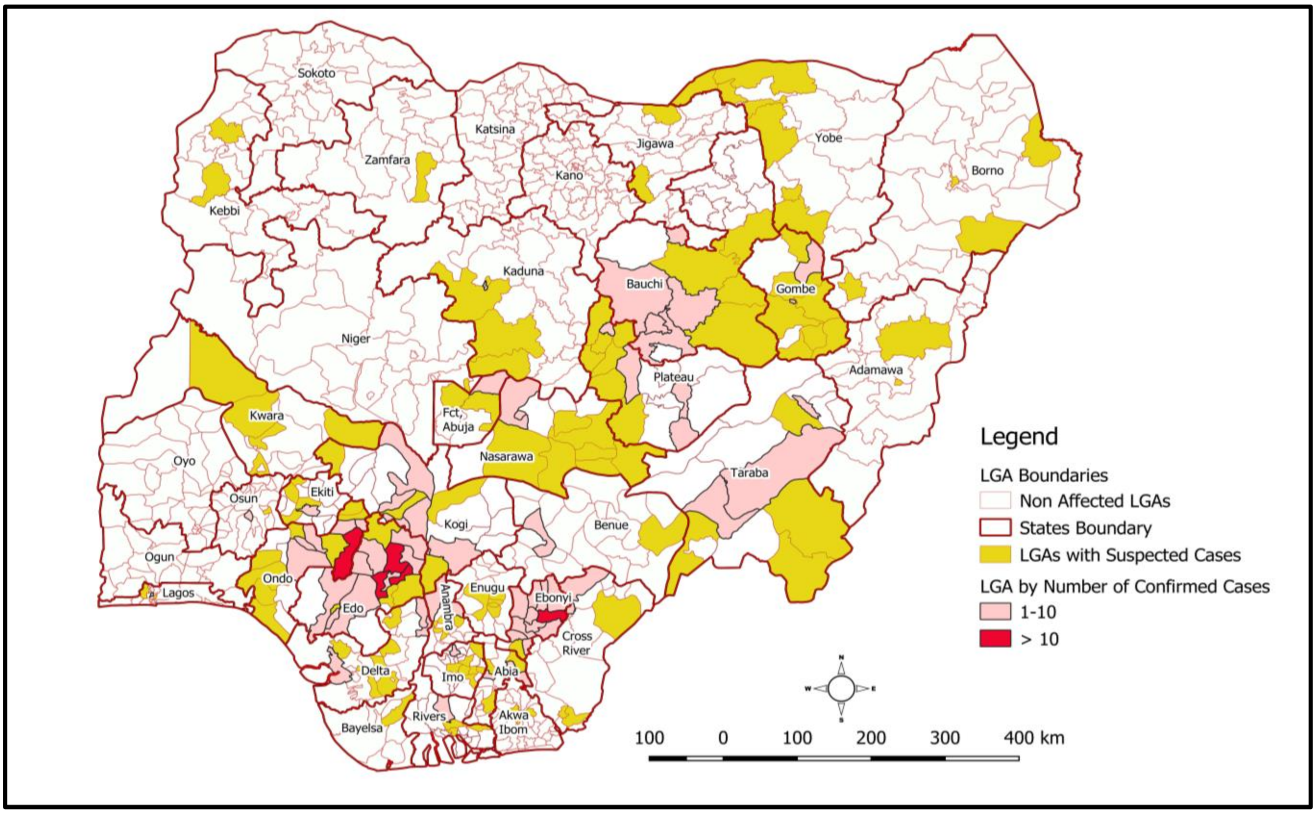

Figure 2. Distribution of Suspected and Confirmed Lassa Fever cases in Nigeria by LGA

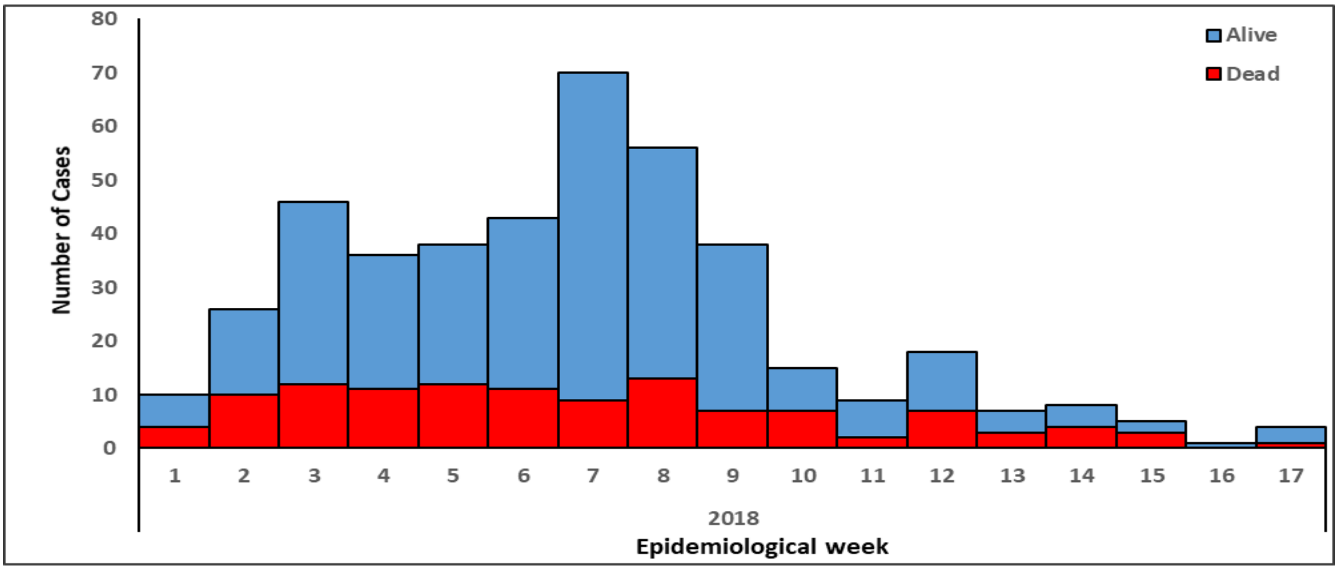

Figure 3. Confirmed (420) and Probable (10) Cases of Lassa fever by epidemiological week in Nigeria week 1- 17, 2018

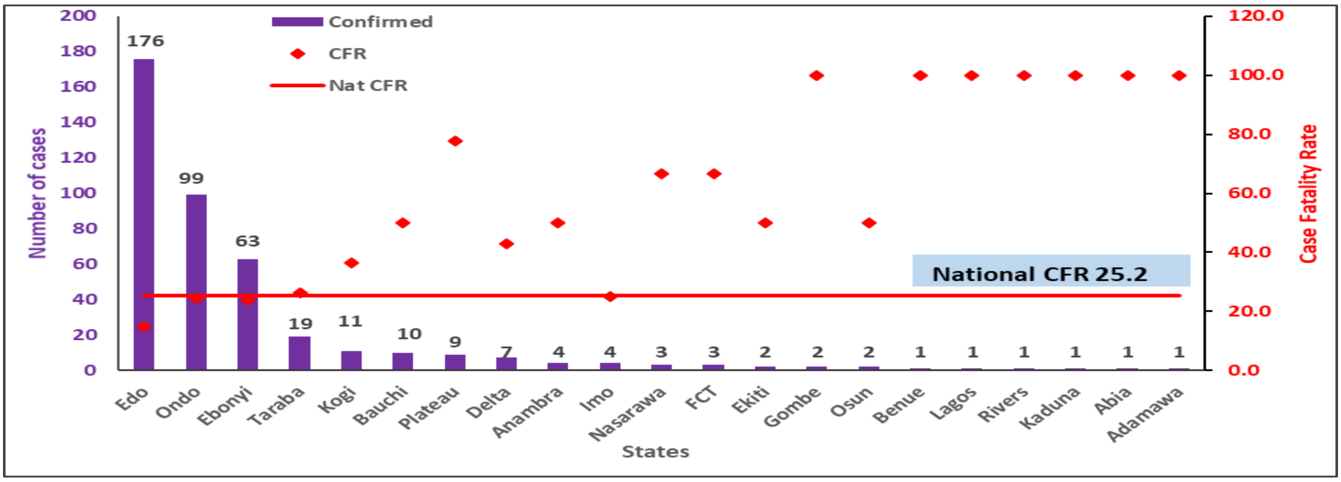

Figure 4. Confirmed Lassa fever cases in Nigeria with state specific Case Fatality Rates (CFR) as at 29th April 2018

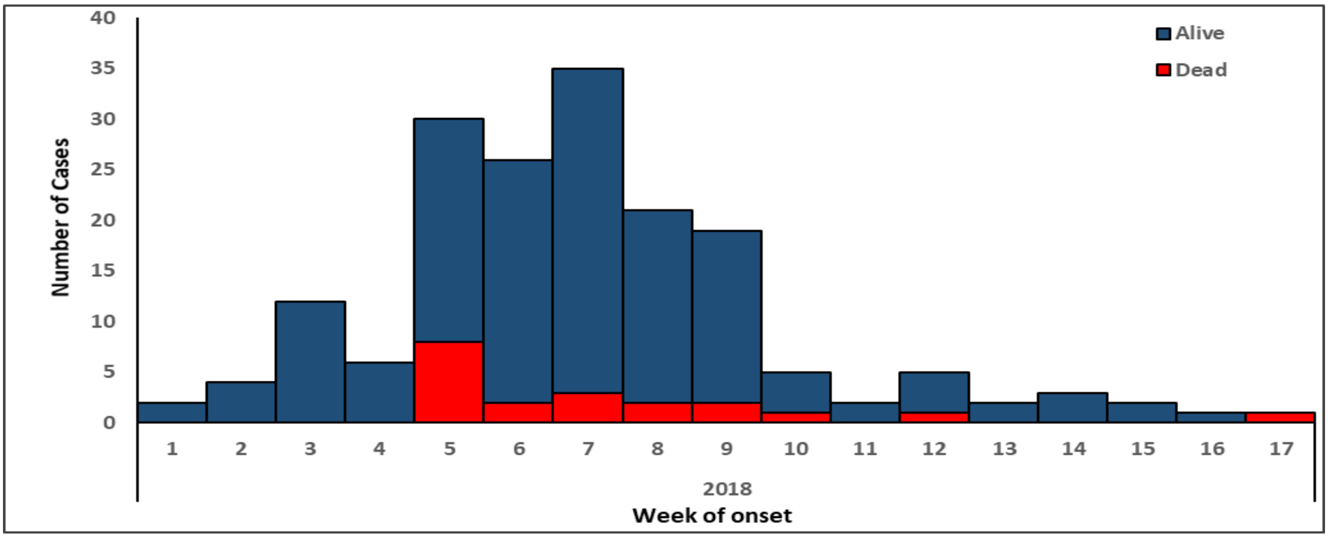

Figure 5: Epicurve of confirmed cases Lassa fever in Edo State week 1-17, 2018

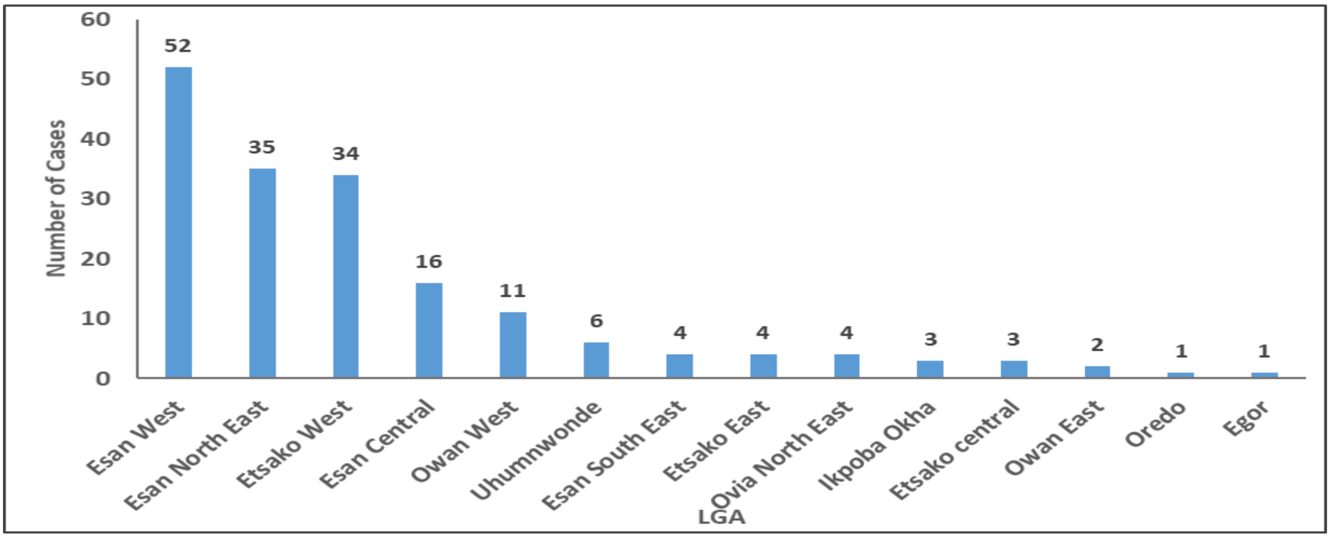

Figure 6: Distribution of Lassa fever confirmed cases in Edo State by LGA as at 29th April 2018

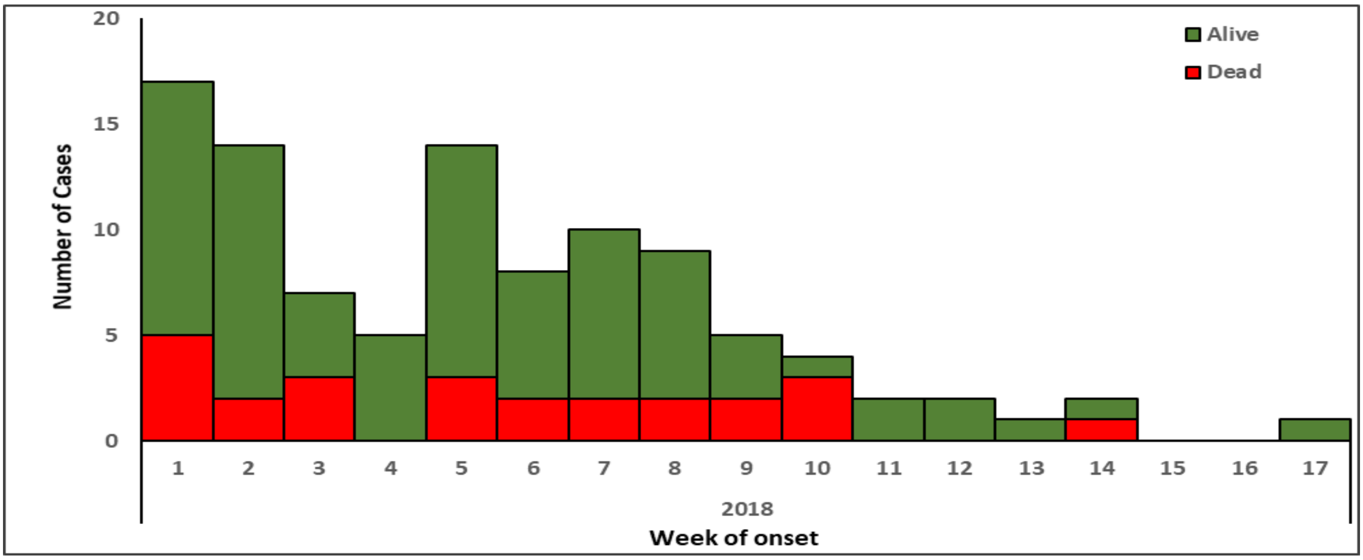

Figure 7: Epicurve of Lassa fever confirmed cases in Ondo State week 1- 17, 2018

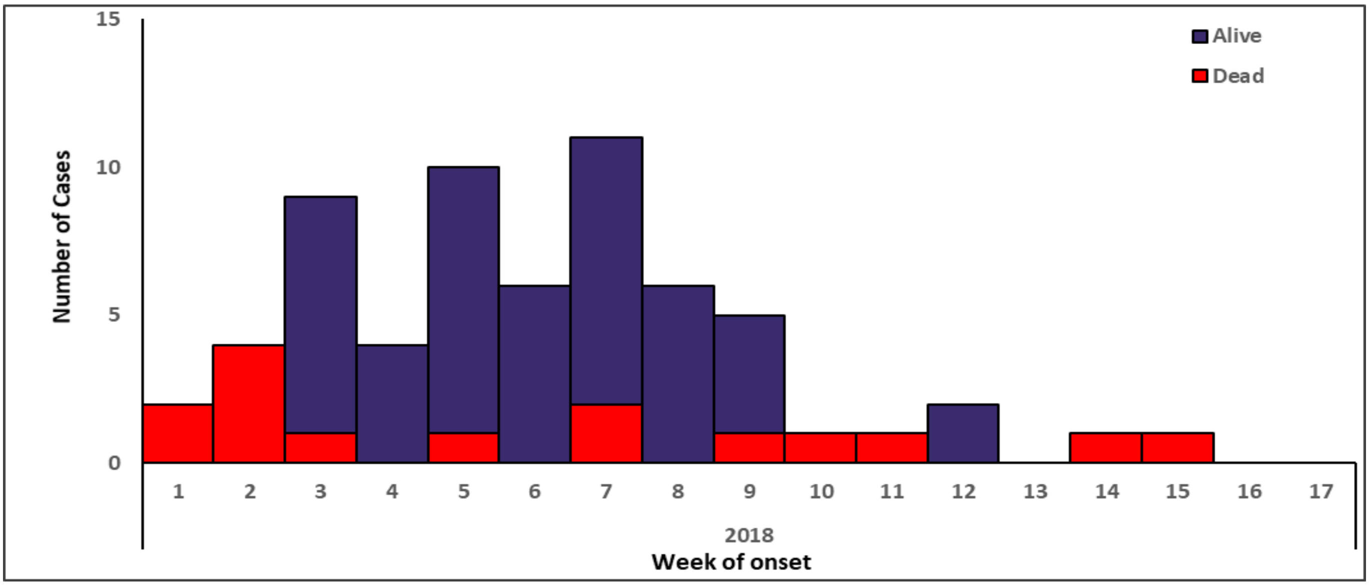

Figure 8: Epicurve of Lassa fever confirmed cases in Ebonyi State week 1-17, 2018

[i] *Suspected case describes any individual presenting with one or more of the following: malaise, fever, headache, sore throat, cough, nausea, vomiting, diarrhoea, myalgia, chest pain, hearing loss and either a. History of contact with excreta or urine of rodents b. History of contact with a probable or confirmed Lassa fever case within a period of 21 days of onset of symptoms OR Any person with inexplicable bleeding/hemorrhagia.*

[ii] *Any suspected case with laboratory confirmation (positive IgM antibody, PCR or virus isolation)*

[iii] *Any suspected case (see definition above) who died without collection of specimen for laboratory testing*

[iv] *"Active" means where there has been at least one confirmed case, and contacts within 21 days post exposure*

*** Backlog of a probable case reported from Adamawa state**

SITUATION REPORT
Nigeria Centre For Disease Control (NCDC)
www.ncdc.gov.ng PLOT 800 EBITU UKIWE STREET, JABI ABUJA, NIGERIA. TOLL FREE CALL: 0800-970000-10. E: info@ncdc.gov.ng @NCDCgov

TITLE:	2018 LASSA FEVER OUTBREAK IN NIGERIA
SERIAL NUMBER:	18
EPI-WEEK:	18
DATE:	6th May 2018

HIGHLIGHTS

- **In the reporting Week 18 (April 30-May 06, 2018) three new confirmed[ii] cases** were reported from two states -Edo(2) and Ebonyi (1) with **no new death.**
- From 1st January to 6th May 2018, a total of 1894 **suspected[i] cases** have been reported from 21 states**.** Of these, **423 were confirmed positive, 10 are probable, 1460 are negative** (not a case) and 1 is awaiting laboratory result (pending)
- Since the onset of the 2018 outbreak, there have been 106 deaths in confirmed cases, 10 in probable cases. Case Fatality Rate in confirmed cases is 25.1%
- 21 states have recorded at least one confirmed case across 70 Local Government Areas (Edo, Ondo, Bauchi, Nasarawa, Ebonyi, Anambra, Benue, Kogi, Imo, Plateau, Lagos, Taraba, Delta, Osun, Rivers, FCT, Gombe, Ekiti, Kaduna, Abia and Adamawa). **Eighteen** states have exited the active phase of the outbreak while three States remain active[iv] - *Figure 1*
- In the reporting week 18, **no** new healthcare worker was infected. **Thirty-seven health care workers have been affected since the onset of the outbreak in eight states** –Ebonyi (16), Edo (12), Ondo (3), Kogi (2), Benue (1), Nasarawa (1), Taraba (1), and Abia (1) with eight deaths in Ebonyi (6), Kogi (1) and Abia (1)
- 81% of all confirmed cases are from Edo (42%) Ondo (23%) and Ebonyi (16%) states
- Seven cases are currently being managed in four treatment centres across the country
- A total of **5012** contacts have been identified from 21 states. Of these **106** (2.1%) are currently being followed up, 4895(97.7%) have completed 21 days follow up while 11(0.2%) were lost follow up. 81 symptomatic contacts have been identified, of which **28 (36%)** have tested positive from five states (Edo-13, Ondo-8, Ebonyi-3, Kogi -3 and Bauchi-1)
- National RRT team (NCDC, WHO and NFELTP residents) batch D continues to support response in Plateau, Kogi, and Adamawa states
- National Lassa fever multi-partner multi-agency Emergency Operations Centre (EOC) continues to coordinate the response activities at all levels, **however response has been de-escalated** due to continual decline of cases over the past 11 weeks

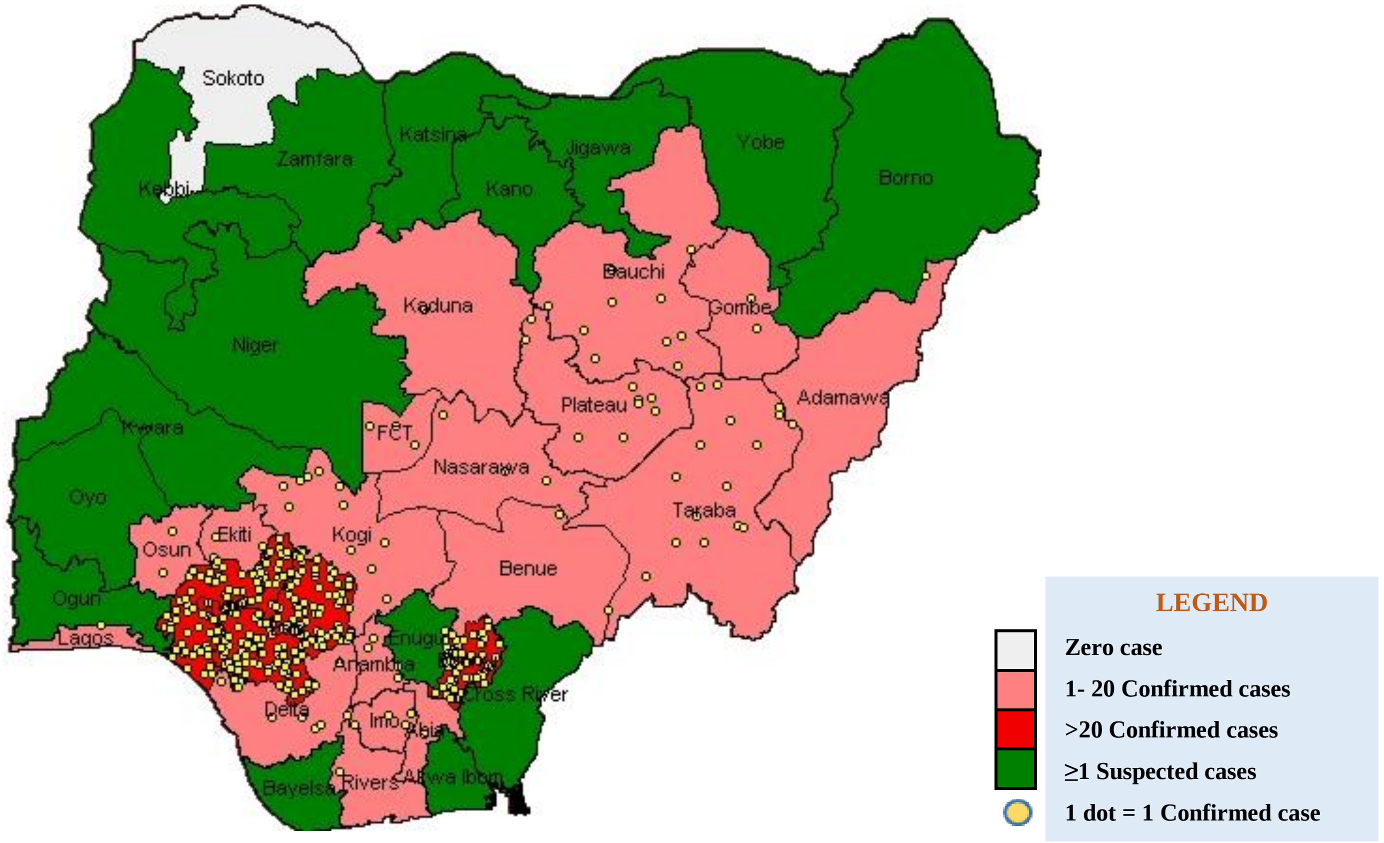

Figure 1. Distribution of Confirmed Lassa Fever cases in Nigeria as at 6th May, 2018

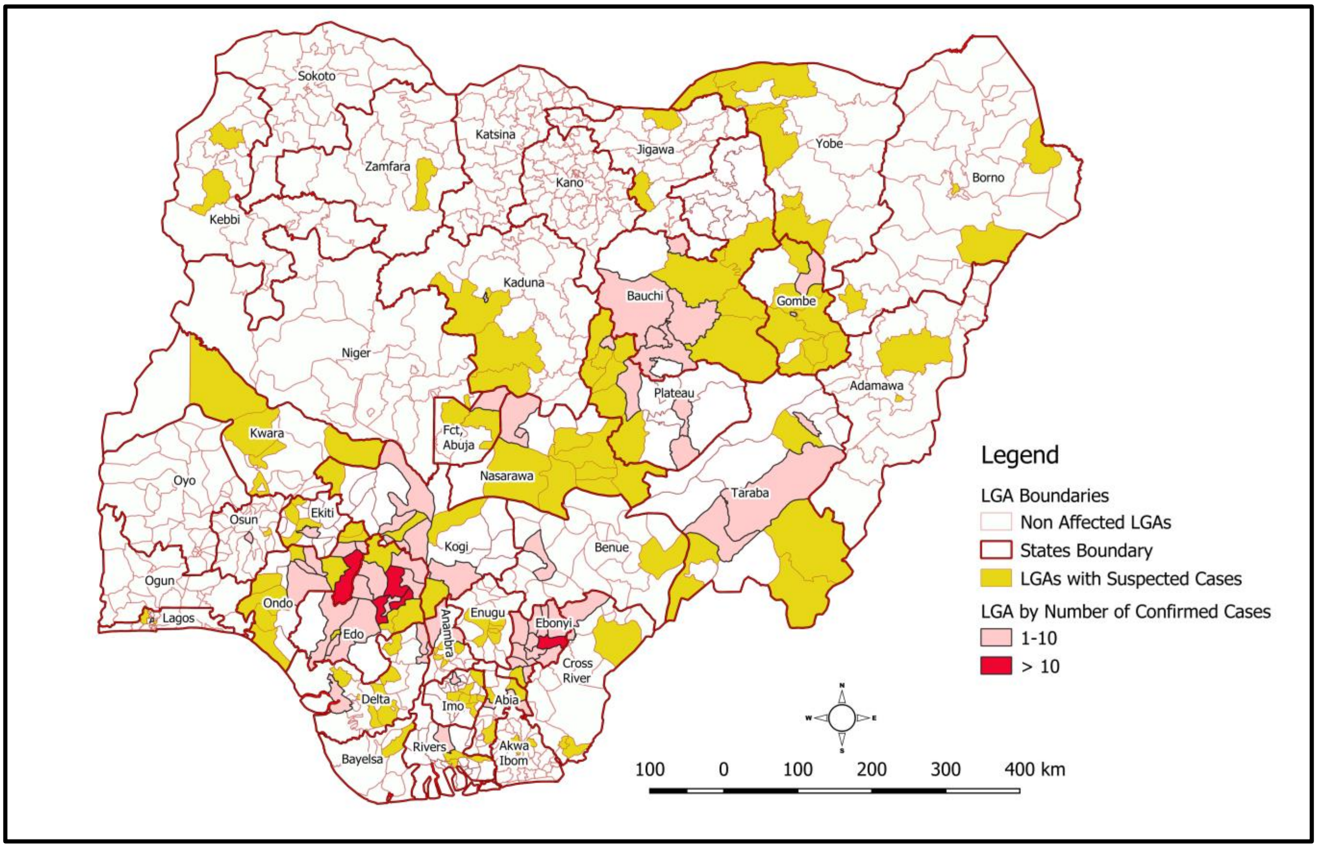

Figure 2. Distribution of Suspected and Confirmed Lassa Fever cases in Nigeria by LGA

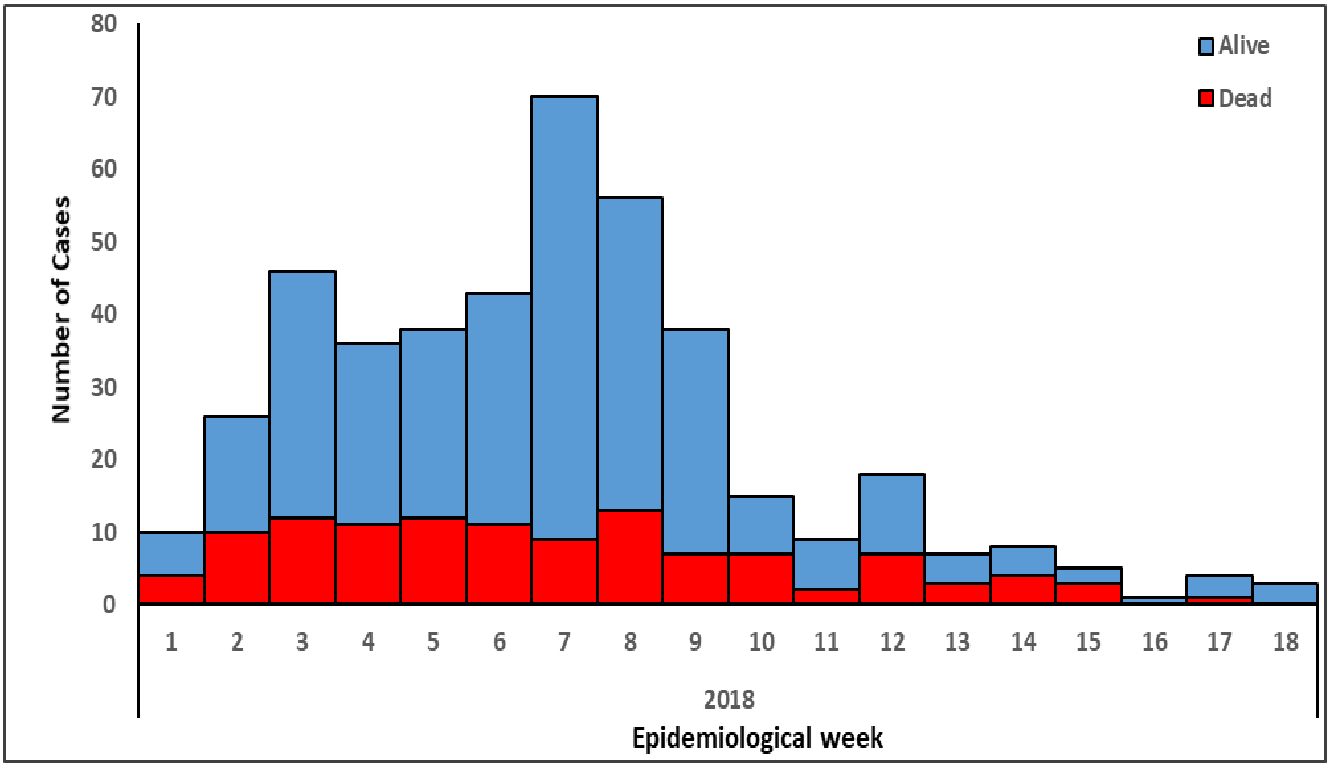

Figure 3.Epicurve of Lassa fever Confirmed (423) and Probable (10) Cases in Nigeria week 1- 18, 2018

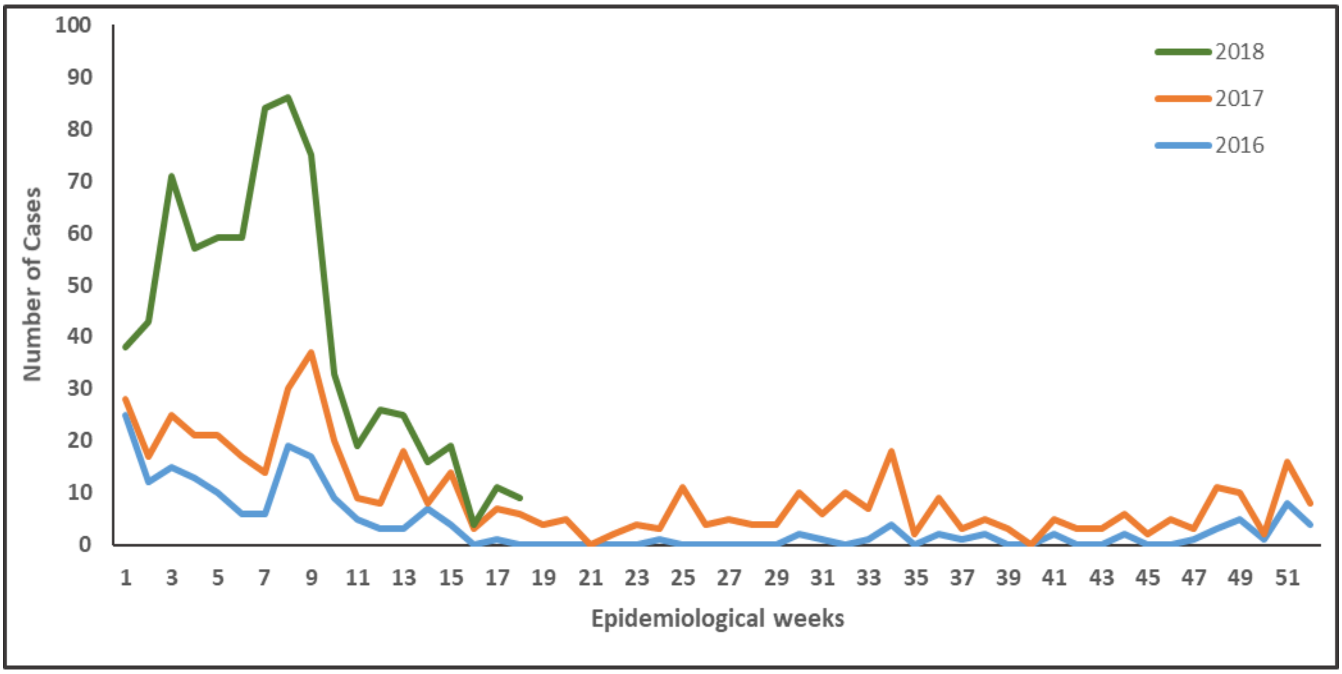

Figure 4. Trends of Lassa fever Confirmed Cases in Nigeria, 2016-2018

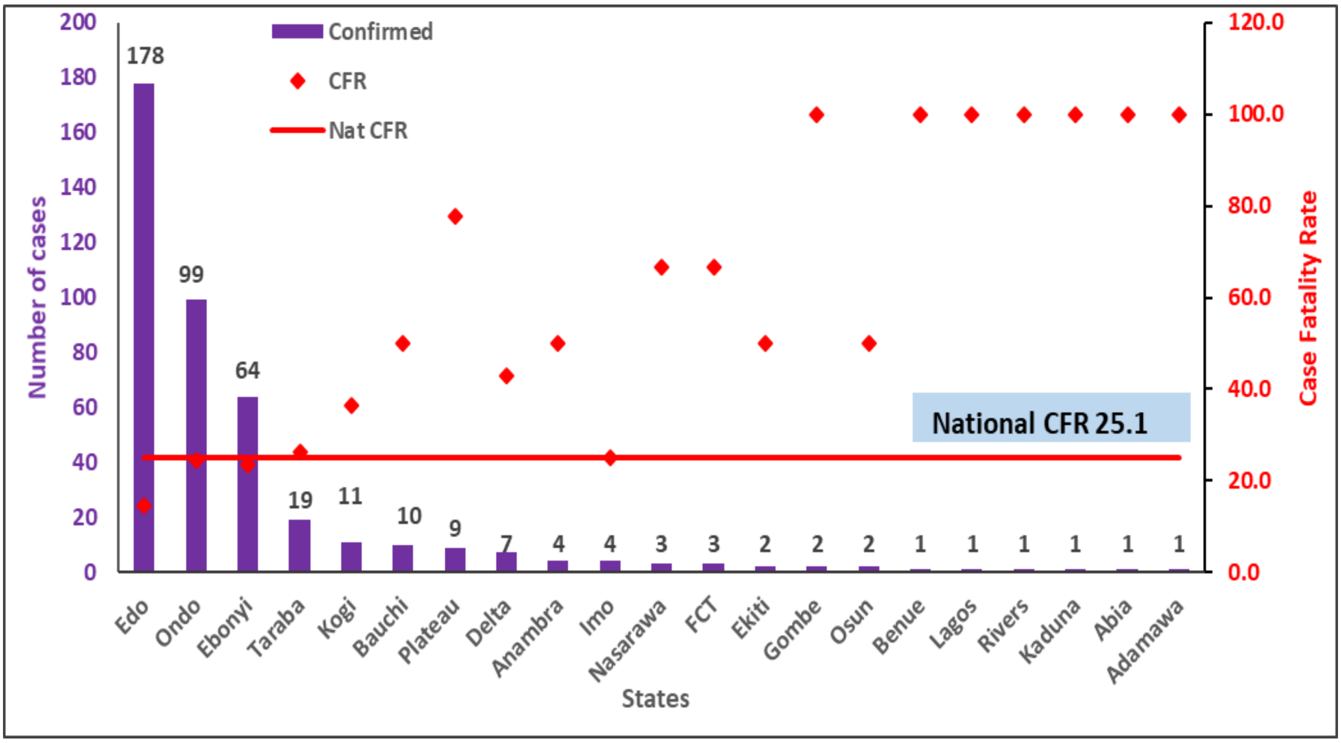

Figure 5. Confirmed Lassa fever cases in Nigeria with state specific Case Fatality Rates (CFR) as at 6th May 2018

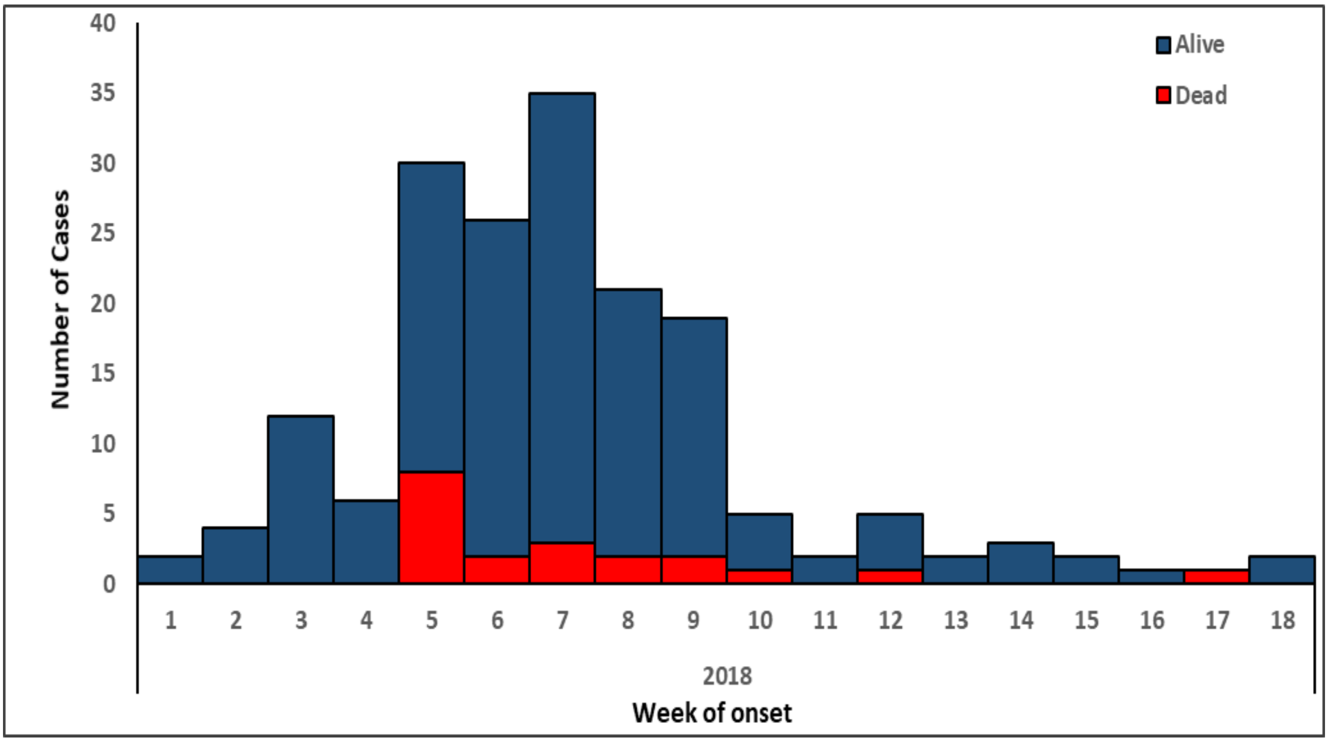

Figure 6: Epicurve of confirmed cases Lassa fever in Edo State week 1-18, 2018

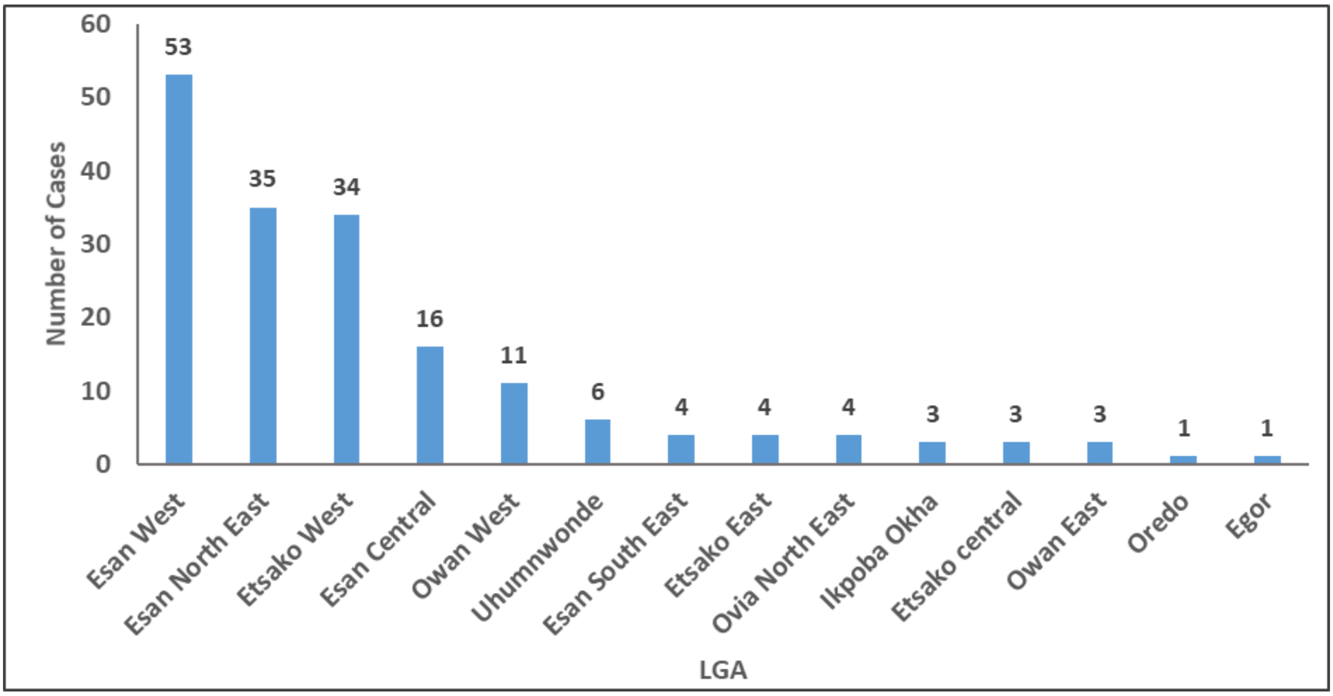

Figure 7: Distribution of Lassa fever confirmed cases in Edo State by LGA as at 6th of May 2018

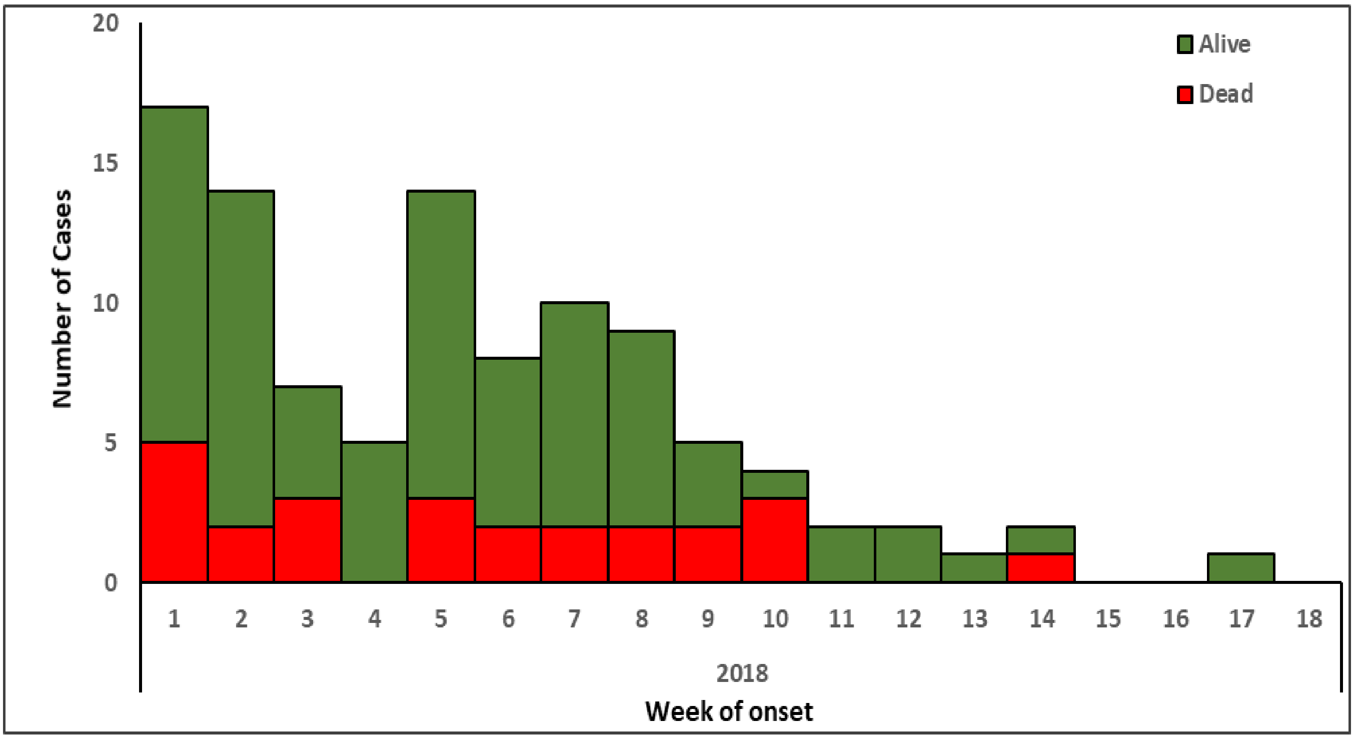

Figure 8: Epicurve of Lassa fever confirmed cases in Ondo State week 1- 18, 2018

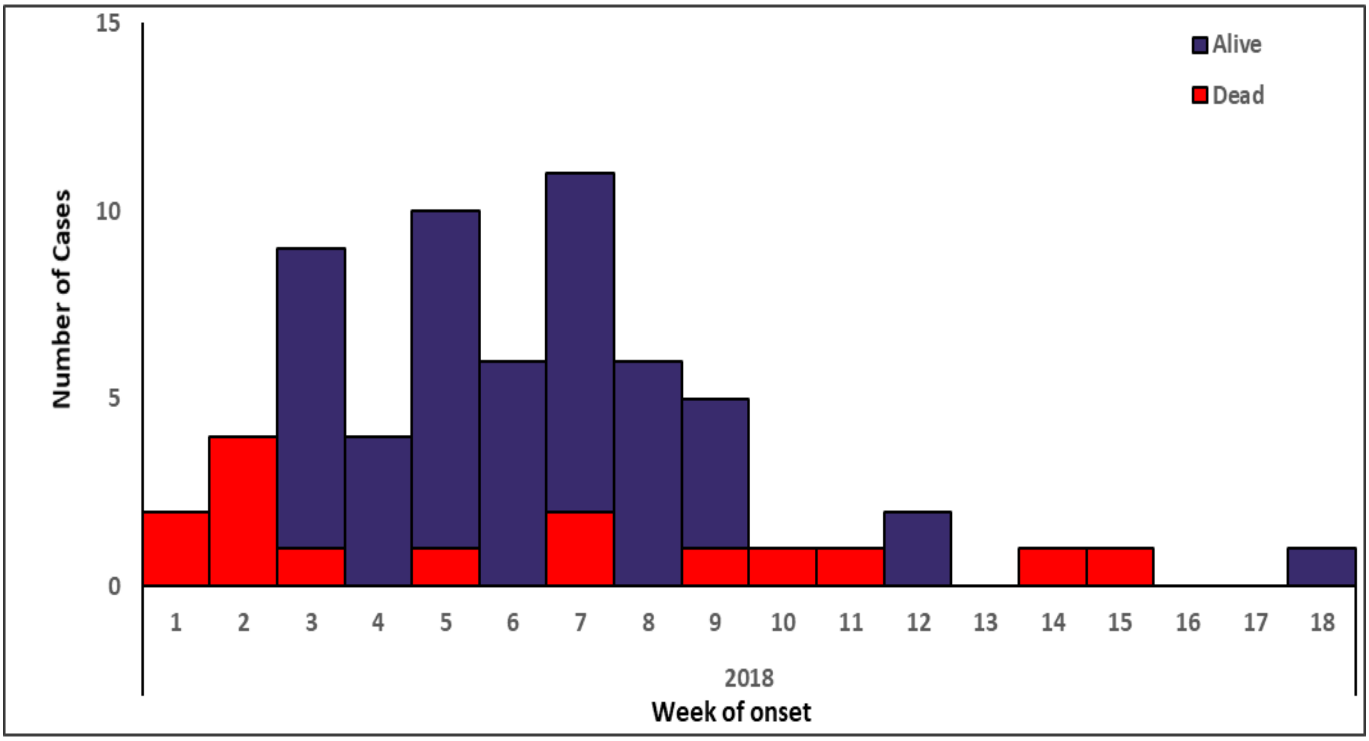

Figure 9: Epicurve of Lassa fever confirmed cases in Ebonyi State week 1-18, 2018

---

[i] *Suspected case describes any individual presenting with one or more of the following: malaise, fever, headache, sore throat, cough, nausea, vomiting, diarrhoea, myalgia, chest pain, hearing loss and either a. History of contact with excreta or urine of rodents b. History of contact with a probable or confirmed Lassa fever case within a period of 21 days of onset of symptoms OR Any person with inexplicable bleeding/hemorrhagia.*

[ii] *Any suspected case with laboratory confirmation (positive IgM antibody, PCR or virus isolation)*

[iii] *Any suspected case (see definition above) who died without collection of specimen for laboratory testing*

[iv] *"Active" means where there has been at least one confirmed case, and contacts within 21 days post exposure*

Disclaimer – ***The information contained in this document is confidential, privileged and only for the intended recipient and may not be used, published or redistributed to the public. A redacted version is available on http://ncdc.gov.ng/diseases/sitreps***
